# Single cell long read whole genome sequencing reveals somatic transposon activity in human brain

**DOI:** 10.1038/s42003-025-08805-2

**Published:** 2025-11-20

**Authors:** Michal B. Izydorczyk, Ester Kalef-Ezra, Dominic W. Horner, Xinchang Zheng, Nadine Holmes, Marco Toffoli, Zeliha Sahin, Yi Han, Heer H. Mehta, Sonja W. Scholz, Clifton L. Dalgard, Donna M. Muzny, Adam Ameur, Fritz J. Sedlazeck, Christos Proukakis

**Affiliations:** 1 Human Genome Sequencing Center, Baylor College of Medicine, Houston, TX USA; 2 Department of Clinical and Movement Neurosciences, Royal Free Campus, Queen Square Institute of Neurology, University College London, London, UK; 3 Aligning Science Across Parkinson’s (ASAP) Collaborative Research Network, Chevy Chase, MD USA; 4 DeepSeq, School of Life Sciences, University of Nottingham, Nottingham, UK; 5 Neurodegenerative Diseases Research Section, National Institute of Neurological Disorders and Stroke, Bethesda, MD USA; 6 Department of Neurology, Johns Hopkins University Medical Center, Baltimore, MD USA; 7 Department of Anatomy, Physiology & Genetics, Uniformed Services, University of the Health Sciences (USUHS), Bethesda, MD USA; 8 The American Genome Center, USUHS, Bethesda, MD USA; 9 Science for Life Laboratory, Department of Immunology, Genetics and Pathology, Uppsala University, Uppsala, Sweden; 10 Department of Computer Science, Rice University, Houston, TX USA

**Keywords:** Genome informatics, DNA sequencing, Movement disorders

## Abstract

The advent of single cell DNA sequencing revealed astonishing dynamics of genomic variability, but failed at characterizing smaller to mid size variants that on the germline level have a profound impact. In this work we discover previously uncharacterized genomic dynamics in 18 cells from three human brains utilizing single cell long-read whole genome sequencing. This provides key insights into the dynamic of the genomes of individual cells and further highlights brain specific activity of transposable elements, but requires validation in larger studies.

## Abstract (web summary)

Long read single cell whole genome sequencing after droplet multiple displacement amplification from human brain reveals somatic transposon activity.

## Introduction

Single cell whole genome amplification (WGA) enables single cell whole genome sequencing (scWGS), typically carried out using short reads at low coverage^1^, which generally only detects Mb-scale CNVs, although identification of CNVs > 50 kbp was reported^2^. In any case, many variants that would be expected, such as Alu or LINE variants are missed. These transposable elements (TE) families are the most abundant and active transposons, collectively accounting for approximately 27% of the human genome^3^ and contributing to recombination in healthy neurons^4^ and neurodegenerative diseases^5–7^. Simultaneously, the advent of long-read sequencing enables the accurate detection of Alu or other transposon-mediated mutations^8^.

Long-read scWGS (scWGS-LR) was recently reported on T-cells, after WGA using isothermal Multiple Displacement Amplification (MDA) in droplets (dMDA), to assemble one genome of a single cell. However, it came at significant cost, and with limited completeness due to chimera and restricted amplicon size^9^. Nevertheless, that opens up the field for further exploration of whether a similar approach can provide additional insights into the genomic variation of single cells, and thus shed light into mosaic mechanisms with potential impact in disease. This is highly relevant since somatic mutations may contribute to neurodegenerative disorders such as synucleinopathies, which include Parkinson’s disease and Multiple System Atrophy (MSA). MSA is a sporadic disease of low heritability^10^ with some possible genetic predispositions^11^. We have already reported somatic CNVs (gains) of the *SNCA* gene in PD and MSA^12,13^, associated with regional and single cell pathology in MSA^14^, and Mb-scale single cell CNVs genome-wide in two MSA cases^13,15^.

In this study, we apply scWGS-LR as a proof of concept to investigate the dynamics of genomic variants across individual brain samples. To enable this, we have developed tailored filtering methods and strategies to avoid known amplification biases. Furthermore, we are comparing the single cell long-read with bulk long-read and single cell short read to assess the variants we identified. Our results highlight multiple classes of variants on a single cell level, including SNVs, small InDels, and larger insertions and deletions. Particularly, we find variants containing sequences of transposable elements implicated in neurodegeneration and normal brain tissue mosaicism. Altogether, this leads to deeper insights into the variability of important genes but further highlights a notable transposon activity in the brain.

## Results and discussion

**Fig. 1:**
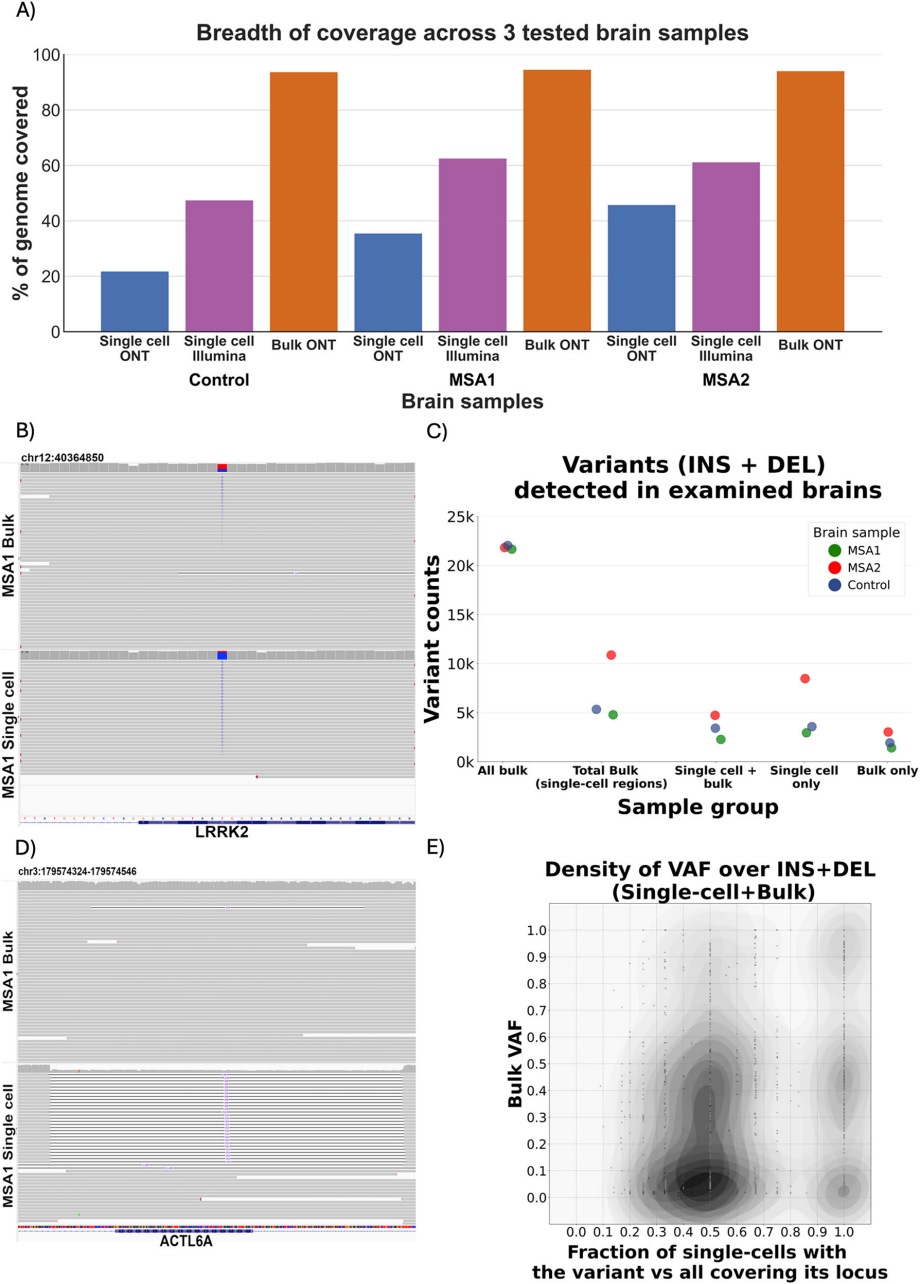
Overall metrics of genomic data obtained in scWGS experiments of studied brains. **A** Breadth of coverage for each of the analyzed cell types, including long-read ONT sequencing and short-read scWGS Illumina sequencing of the same dMDA single-cell DNA. For single cells, analyzed regions are limited to ≥5x depth of coverage, while for bulk all covered regions are included. **B** Mosaic SNV detected in long-read scWGS—aggregate of 6 single cells and bulk ONT MSA1 sample located within an exon of LRRK2—a gene important in monogenic Parkinson’s disease. **C** SVs (insertions and deletions) detected across the 3 studied brains in long-read scWGS and bulk ONT samples. The first category shows all bulk variants across the genome, while the remaining 4 categories are limited to regions covered ≥5x in single cell-samples and variants from those regions in corresponding bulk. **D** Mosaic deletion detected in aggregate of 6 long-read MSA1 ONT single cell samples, as well as in low frequency in corresponding long-read ONT bulk tissue sample. The deletion overlaps ACTL6A gene, encoding actin-related protein associated with Non-Specific Syndromic Intellectual Disability and Torticollis. **E** Ratio between variant allele frequency (VAF) in bulk and their occurrence in single cells, for structural variants detected in single cells and confirmed in corresponding bulk. High VAF population variants were removed. Most of the remaining variants are mosaic in bulk, as represented by the heatmap concentration in the lower half of the chart. Majority of the variants are found in either 50% or 100% of the single cells, due to coverage discrepancies for specific cells - most of the loci are adequately covered (5x minimum) in one or two cells (data available in Supplementary data and Table 32).

To assess genomic variation in scWGS-LR, we isolated single nuclei using CellRaft device and utilized dMDA to amplify their DNA, aiming to reduce amplification bias while maintaining a relatively long molecule length. This variation of isothermal MDA method compartmentalizes single-cell DNA fragments into individual droplets and was already shown to reduce sequencing coverage bias in blood cells^9^. We employed two different library preparations for all cells: T7 endonuclease debranching protocol, the standard method to remove displaced strands created by MDA; and the PCR rapid barcoding protocol (RBP), which creates linear molecules, albeit with limited length, on multiple ONT sequencing devices. We sequenced a total of 18 single cells across the cortex of three brains, two MSA (cingulate cortex) and one control (frontal cortex), where we previously performed Illumina scWGS on other cells using an hybrid WGA method (PicoPLEX)^13^ to enable a comparison. To avoid uneven genome coverage, we pooled and barcoded 6 single cells per ONT flow cell, obtaining 144 million reads, with up-to 5.8 million reads longer than 3 kb, and some as long as 300 kb, with the average N50 of 2.8 kb (Supplementary data, Table 1). This sequencing performance increase enables the utilization of T7 endonuclease debranching as library preparation method, retaining a wider range of read sizes, with 4.5 million reads > 3 kb for MSA2 (5.8 million for control), while RBP protocol yielded 2.7 million reads > 3 kb. Overall, this resulted in covering up-to ~46% of the human genome at 5x coverage or higher across 6 single cells with ONT sequencing. This is only slightly less than Illumina scWGS of the same dMDA cells at ~60% (Fig. 1A). Thus, overall, yielding enough long-read single cell coverage to identify small to mid-size variants. Furthermore, to enable comparisons, we sequenced each of the brain regions from the same donors using bulk ONT sequencing (metrics in Supplementary data, Table 2).

To establish our filters and sequencing strategy we utilized the Genome in a Bottle benchmark (GIAB SNV v4.2.1), performing ONT WGS of low-input HG002 sample after MDA amplification. Doing so we achieved an F-score of 93.4% (94.5% excluding low genotype-quality false-positives) for SNV/InDels (Supplementary data, Table 3). Given these encouraging results, we called variants across single cell and bulk brain samples using the same strategy (see methods, Supplementary data, Table 4). For bulk sequencing alone we obtained mean depth of coverage 53x for MSA1, 23x for MSA2, and 18x for control, and on average 4.72 million SNV/InDels, per brain (Supplementary data, Table 2). When comparing this to the single cell data, we observed similar numbers of SNV/InDels for regions that were covered >5x in both datasets as 1.66 million SNVs/InDels in bulk and 1.42 million SNVs/InDels for single cell data. Overall we observed a high overlap of 70.0% of bulk SNVs/InDels confirmed in single cell data (for example, see Supplementary Figs 1–8). As expected however, we also observed allelic dropouts on the single cell data, with 88.9% of missing SNVs/InDels being heterozygous in bulk data (Fig. 1B shows an example of mosaic SNV confirmed in bulk at VAF = 0.35). On average, over 80% of SNVs/indels in single cell samples were shared with bulk. The remaining single cell only patterns (Supplementary Figs 9–11) might be true single cell events, or very low-level clonal events missed in bulk WGS but could also represent amplification or sequencing errors (Supplementary data, Tables 4–6). To validate SNV/InDel findings, we analysed the same single cell dMDA DNA previously sequenced on short-read Illumina platform^13^. From the high confidence (genotype quality, GQ > 20) calls, 84.8% were validated in Illumina (Supplementary data, Table 4). The distribution of these validated single cell SNV calls showed a predominance of C > T (49.8%) followed by T > C (20.9%) (Supplementary data, Table 7). This suggests a mixture of true single cell events, as C > T and T > C appear to occur with approximately equal frequency in neurons^16^, and MDA errors, which are predominantly C > T^17^.

Additionally, to determine whether some high GQ single-cell only events could actually represent low level clonal mosaics, which would require higher coverage than the ONT WGS data available, we determined the presence of these in bulk Illumina high coverage WGS from the same brain region in MSA1 and MSA2 (coverage 91x and 88x respectively). On average 8.6% of variants with GQ > 20 specific to single cells in ONT experiments were confirmed in corresponding Illumina bulk, with substitution patterns for these 25.2% C > T and 27.6% T > C (Supplementary data, Table 7). This pattern implies that the high GQ single cell ONT SNV confirmed in bulk Illumina do not show the signatures of MDA errors, which are predominantly C > T^118^, and represent true somatic clonal events. Overall, we could show that we are able to identify SNVs/InDels in a single cell, some of them being mosaic (Supplementary Figs 1–8) or absent in bulk (Supplementary Figs 9–11).

On average 34,560 shared between single cells and bulk, 14,898 bulk-only, and 7,940 single-cell specific SNVs/indels, overlapped with exons (Supplementary data, Table 8). The single cell exonic calls were significantly depleted compared to genomic distribution in randomized permutation test (5/5 tests, p < 0.05, no significance for variants specific to ONT-LR with high GQ, Supplementary data, Table 9**)**. We found that loci of 4 genes (exons) previously linked to neurodegenerative diseases contained more than 2 SNVs/InDels, found only in single cells (Supplementary data, Table 10). One gene of interest was *LRRK2*, which is the commonest cause of monogenic Parkinson’s disease and a risk factor for sporadic cases^18–21^, and has a role in progression of progressive supranuclear palsy (PSP)^22^. This exhibited 85 exonic small variants (less than 50 bp long), with 65 in MSA brain samples (31 found only in single cells). *LRRK2* is the commonest cause of monogenic Parkinson’s disease^18–21^.

Next, we assessed SV calling on scWGS-LR data. This is complicated as potential chimera from the MDA amplification can lead to false positives^23^. To identify this, we benchmarked our approach with the well established HG002 and the GIAB benchmark. Overall we reached 87.8% F-score for genome-wide SV (GIAB SV v0.6^24^) (see Supplementary data, Tables 11–13). Even when assessing the more stringent CMRG (Challenging Medically Relevant Genes) benchmark, we achieved an F-score for SV of 89.5%^25^, thus showing a low rate of chimera after our filtering impacting SV results. Using the same approach on brain samples, we discovered an average of 12,514 insertions and 9331 deletions in bulk samples (Supplementary data, Table 2). For regions where bulk and single cell long-read have >5x coverage we identified an average of 2414 insertions and 1965 deletions in bulk samples compared to 2250 insertions and 2821 deletions for single cell samples (Fig. 1C). We found that 78.5% of single cell insertions and 50.9% of deletions can be recalled in corresponding bulk samples (Fig. 1D) across the experiments, including the previously reported mosaic deletion between a reference Alu and a germline Alu insertion^26^. After removing common population variants, most of the remaining are mosaic in bulk and can be found in about 50% of the studied single cells, with a smaller proportion of variants found in all of them (Fig. 1E). This is also affected by the fact that, on average, most reads at a given locus come from only two individual cells. The interesting result from this figure is that it highlights the fact that multiple single cell SVs are indeed identifiable via low VAF SV in bulk tissue. Nevertheless, the SVs identified specifically in scWGS-LR once again underscore the diversity of brain tissue. Out of the discovered bulk only variants ~80% (on average, 73.1% insertions and 87.3% deletions) were heterozygous, consistent with allelic dropout (Supplementary data, Tables 14 and 15). Interestingly, in contrast to small InDels (Supplementary data, Table 16) we found an excess of single-cell-only deletions; structural variants discovered in single cells-only averaged 525 insertions and 1433 deletions. Visual inspection confirms the presence of these variants in tested single cell samples and absent in bulk (For examples of deletions, refer to Supplementary Figs 12–35). For validation purposes, we reviewed short-read paired-end scWGS data after PicoPLEX WGA from the same brains and regions. Despite the limited resolution of short reads, 6.37% of deletions present in single cells and bulk long-read data were also found in short-read PicoPLEX data and confirmed by visual review. Among the long-read single-cell only deletions, although 2.3% of MSA1 and 0.92% of MSA2 were supported in at least one PicoPLEX cell, none were confirmed visually (Supplementary data, Table 17). Comparing variant patterns detected in brains to clonally expanded CD8 + T-cells, studied by Hård et al.^9^, we found that single-cell only deletion/insertion ratio is on average 5.73x higher in tested brains. In contrast, the bulk sequencing of our brains shows the expected imbalance towards the insertions^27–29^; genome-wide ratio of DEL/INS in bulk MSA and control brain is 0.75 (Supplementary data, Table 2). Moreover, on average 3.47% of single cell deletions confirmed in bulk can be found in exonic sequences, while 4.94% of all bulk deletions overlapped exons (Supplementary data, Table 18). The difference was smaller in case of insertions, as 2.50% of single cell insertions confirmed in bulk and 2.40% of all bulk insertions were located within exons (Supplementary data, Table 19). For most single-cell-only variants, we observed significant depletion in exons compared to genomic exon distribution (permutation test, *p* < 0.05–3/5 tests involving single-cell-only insertions (Supplementary data, Tables 20, 21) and 5/5 tests involving single-cell-only deletions indicate significant depletion in exons; Supplementary data, Tables 22, 23). Across the examined variants, we found several deletions overlapping genes, albeit within their intronic sequences, which can potentially affect various neurological processes (Supplementary data, Table 24).

**Fig. 2:**
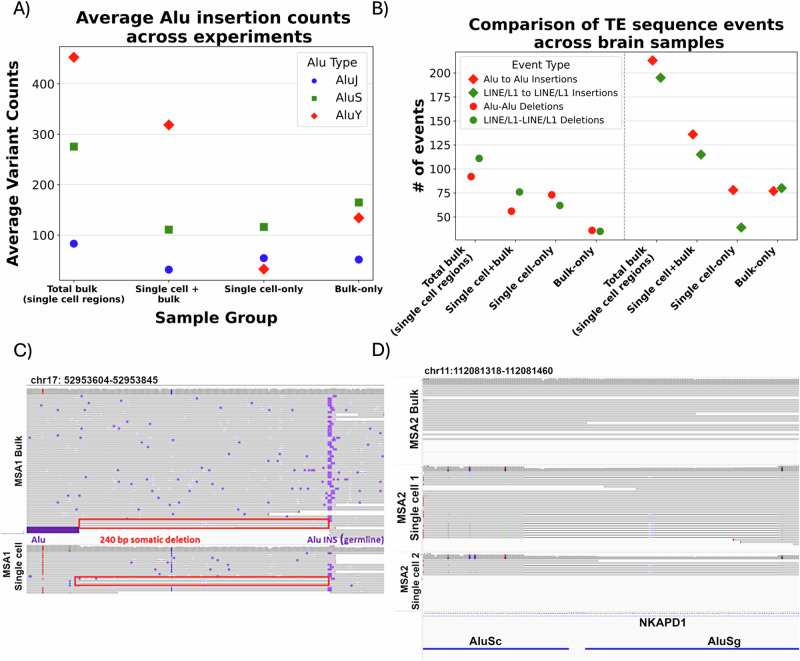
Common transposable element families can influence structural variation by inserting or deleting from the genome, especially via NAHR-mediated deletions. **A** Subfamilies of SINE/Alu insertions, detected in the single cell samples. The majority of insertions found in bulk or single cells and confirmed in bulk belonged to AluY family, however insertions private to single cells predominantly belonged to AluS subfamily. **B** Comparison of detected recombinational deletion events and TEs inserting into loci occupied by the same TE type in the reference genome. We find more SINE/Alu elements to insert within similar loci, presumably due to their abundance and ongoing transpositional activity. However, recombinational activity is higher for LINE/L1 in all samples found in bulk, with slightly less events private to single cells, where SINE/Alu is more active. **C** Confirmed germline insertion of Alu element, which overlaps a breakpoint of subsequent somatic deletion; its other breakpoint overlaps reference Alu element, which then causes recombinational deletion - presented reads are bulk ONT MSA and an aggregate of data from 6 single cells. **D** Recombinational deletion detected in MSA2 single cell sample and absent in bulk. The deletion present in samples of both library preps of single cells (aggregates of 6 single cells each; T7 in the middle and RBP at the bottom) overlaps with AluSc and AluSg TEs on its breakpoints, suggesting that it occurred due to their recombination. It is within NKAPD1 gene, involved in delayed development syndrome, ataxia and hypotonia.

Transposable elements have been previously implicated in neurogenesis and neurodegeneration, as they have a propensity to facilitate Non-Allelic Homologous Recombination (NAHR) events leading to deletions in neurons^30^, and can also create de novo somatic insertions if active. Nevertheless, their occurrence has only been studied in single cell data from the brain with short reads^31,32^. To investigate transposon activity in the neuronal tissue, we annotated members of two most abundant human TEs, LINE/L1 and SINE/Alu, within the detected SV. On average, across the 2 MSA brains and 1 control, we found 332 bulk insertions and 378 bulk deletions to contain LINE/L1 sequences. Collectively, in the corresponding single cell samples of these brains, we captured 261 insertions and 686 deletions that represent LINE/L1 fragments (for detailed breakdown of the reported TEs see Supplementary text section 1). We only managed to recover truncated fragments of LINE/L1 insertions due to limited read length. Nevertheless, we identified many full-length SINE/Alu members (Supplementary data, Table 25). Single cell Alu insertions confirmed in bulk were mostly represented by the evolutionarily youngest AluY family, on average found in 382 variants’ sequences (69% of Alu-containing insertions found both in bulk and single cells, on average). Nevertheless, for single cell-only, the majority of Alu insertions (134, 55% of total single cell-only Alu-containing insertions, on average) were AluS (Fig. 2A). We speculate that this is due to their abundance (~5:1 ratio of AluS to AluY in reference genome^33^ combined with recombinational activity, which surpasses the propensity of other SINE/Alu members to proliferate in the genome (Supplementary data, Table 26).

Further, we were interested in investigating locations in the reference genome where TE-related SVs were found. Initially, we searched for the presence of reference TEs at the locations of detected insertions (Fig. 2B), as existing germline LINE1 sequences have been said to attract novel somatic TEs within the same family^34^ Analysis of TE insertion locations in our brain samples showed 28.7% of single cell Alu insertions confirmed in bulk and 51.1% single cell-specific SINE/Alu insertions occurred within Alu reference elements (for LINE/L1, 67.9% single cell confirmed in bulk and 30.5% of single cell-specific occurred within reference LINE/L1, respectively). This represents an average enrichment of 3.26-fold of Alu and LINE1 insertion into a similar reference sequence, rather than randomly in the genome (assuming 17% of the reference genome is LINE1 and 11% is SINE/Alu), consistent with the data showing that reference elements attract novel SINE/Alu and LINE/L1 insertions (Supplementary data, Table 14). Conversely, of the insertions within SINE/Alu and LINE/L1 in reference, 46.9% of single cell-specific events contained SINE/Alu sequence (79.6% of single cell confirmed in bulk), while for single cell-specific LINE/L1 it was 46.5% (39.5% for LINE/L1 confirmed in bulk).

To gain insight into the nature of observed deletions we examined their sequences, specifically searching for pairs of transposable elements belonging to the same families/types and overlapping deletions’ breakpoints, thus suggestive of being able to facilitate recombinational events. Our previous findings already hinted at NAHR mediated repeat recombinants events^35^ between two SINE/Alu elements (Fig. 2C). As before we could identify these events as mosaic in bulk but also identifiable in single cell data sets. Across three brains, we detected an average of 56 deletions with SINE/Alu pairs and 76 deletions with LINE/L1 pairs at their breakpoints. These deletions are present in single-cell sequencing and mosaic in the corresponding bulk samples. Thus, maybe not surprisingly, on average across 3 tested brains we also identified NAHR mediated repeat recombinants (Fig. 2D) present in single cells only with 73 for SINE/Alu (17.6% of single cell-specific Alu deletions; see Supplementary Figs 36–38) and 62 for LINE/L1 (12.9% of single cell-specific LINE/L1 deletions; see Supplementary Figs 39–41). To validate the reported associations between transposable elements and detected variants within single cells we performed a permutation test over deletions; we found that in 17 out of 20 single-cell comparisons, these were significantly transposon-associated (see methods, Supplementary data, Tables 27–30). This overall highlights the dynamic nature of transposons and their importance to be studied from bulk down to single cell data sets.

To summarize, we demonstrated the utility of long-read single cell whole genome sequencing using Oxford nanopore to study SNV, InDel, and SV mutations across 18 single cells derived from 3 brains. To enable this, multiple innovations were necessary, including an improved filtering pipeline to mediate the effects of MDA amplification, such as chimera formation and single base errors. Furthermore, uneven amplification led to locus dropout and limited coverage, which was mitigated by pooling together single cells in each experiment. To overcome amplification limitations and obtain wide coverage, we employed two ONT long-read scWGS approaches, utilizing different library preparation methods, T7 debranching, which has been used for MDA before, and a PCR-based method, which allows lower input and removes the problem of side-chain formation. We show that the ONT PromethION sequencing device surpasses MinION in produced DNA yield (65 million vs 19 million reads per 6 single cells; Supplementary data, Table 1). This sequencing performance increase enables the utilization of T7 endonuclease debranching as a library preparation method, retaining a wider range of read sizes (for details, see long-read ONT analysis of human brain samples methods section, Supplementary Figs 42–47). As shown on Supplementary Figs 48-65 the increased DNA yield, particularly for longer reads, recovers insertions up to 5 kb and longer, in contrast to MinION device with RBP protocol limited to 1 kb insertions. Therefore, while less portable and more costly, the increased yields of the PromethION device enable more thorough analysis of variants in single cells, particularly targeting longer insertions and offering significantly better breadth of coverage (see Supplementary data, Tables 2 and 4 and Supplementary Figs 66–69). However, improvements to dMDA amplification method aiming to decrease chimeric read formation and locus dropout would help to mitigate limitations of current scWGS-LR studies. This would greatly benefit both long-read approaches by providing a more robust signal, reducing the necessity of strict post-processing, and making it more easily reproducible across a variety of samples.

Collectively, the approaches proposed here contribute to comprehensive characterisation of the mutational landscape in single cells from the brain, reveal insights into somatic SV and their relation to transposons at the single cell level, providing single-cell long-read support for previous work which reported that NAHR can occur between Alu and LINE1 elements, leading to deletions^30^ and that LINE1 insertions preferentially occur within reference LINE1 elements^34^. We highlighted multiple insights across repeat recombinants in the brain, which are identifiable as low VAF in bulk but easier to identify in single-cell sequencing. These mutations are regularly overlooked in standard short-read single-cell sequencing, given its focus on large CNVs. These mutations can impact exons that are getting deleted in a fraction of cells, although they are generally depleted in exonic locations. While this study lacks the power to conclude something for MSA, and indeed the conclusions need to be seen as preliminary due to the small sample size and lack of diverse cell type analysis, it highlights the pitfalls of short read sequencing, which would miss of these mosaic or single cell SV. Overall, we found that there is indeed an increased level of deletions in brain single cell data compared to T-cell single cell data (5.73-fold increase) that was previously published^9^. Further studies need to investigate if this is due to the limited regeneration capacity of neurons. Nevertheless, these findings can be significant given previous reports for the potential mosaic or single cell mutational impact on neurological diseases^12,14,15,17^.

## Methods

### Sequencing

#### Single cell sequencing

We used previously generated dMDA amplicons from single nuclei from two MSA samples (cingulate cortex) and one control (frontal cortex), *n* = 6 cells from each, which had already undergone short-read low-coverage WGS^13,34^. These were derived from fresh frozen *post-mortem* brain samples provided by the Queen Square Brain Bank, London, UK. All donors had given informed consent for the use of their brain in research and ethics approval was provided tissue bank by the UK National Research Ethics Service (07/MRE09/72). All ethical regulations relevant to human research participants were followed. More precisely, nuclei were extracted from 30 to 50 mg frozen brain tissues, counterstained with 1 μg/ml DAPI for 20 min on ice, seeded onto a 10,000-raft CytoSort array (Cell Microsystems) and allowed to settle at least overnight at 4 °C. Then, single nuclei with a neuronal appearance (large diameter and presence of low condensed chromatin) were selected and isolated using CellRaft system (Cell Microsystems) system mounted on a Nikon Eclipse TE300 inverted microscope coupled to a CCD camera (KERN optics). The genome of each nucleus was amplified using dMDA kit and XdropTM instrument according to the manufacturer protocol (Samplix), but with an addition of a heat lysis step (95 °C for 3 min followed by 10 min cool down at RT) after alkaline lysis of the nuclei. All dMDA amplicons were assessed using Qubit dsDNA BR kit (Thermo Fisher Scientific) and TapeStation (Agilent), and stored at −20 °C prior to library preparation.

The dMDA products were purified using Ampure XP beads, using a 0.8x bead to sample ratio, and underwent library preparation using two different methods. RBP (rapid barcoding PCR^35^; or T7 debranching^36^. For RBP, 5 ng of purified DNA was used with the ONT rapid barcoding PCR kit (Oxford Nanopore Technologies, SQK-RBP004) following the manufacturer’s instructions. Libraries were pooled to a final molarity around 100 fmol in 10 μl, assuming an average library size around 2 kb. Pools were sequenced on a MinION MK1b (Oxford Nanopore Technologies, MIN-101B) using an R9.4.1 flow cell (Oxford Nanopore Technologies, FLO-MIN106D). For T7, 500 ng DNA input was used with 1 unit T7 endonuclease (NEB) in 30 μl and incubated at 37 °C for 1 h, followed by bead purification. All recovered DNA was used for library preparation according to the “sequencing gDNA – Native Barcoding Kit 24 V14; Version: NBE_9169_v114_revQ_15Sep2022” protocol, followed by native barcode (SQK-NBD114.96) and sequencing adapter ligation. The MSA1 pool was sequenced on a MinION MK1b R9.4.1 flow cell (FLO-MIN106D), but, due to the low yield, the MSA2 and control pools underwent PromethION sequencing using a R10.4.1 M flow cell for each (FLO-PRO114M) at the Nottingham University DeepSeq core, with a nuclease flush and reload to maximize yield. Note that, due to the high DNA input required, the MSA1 T7 sequencing could not be repeated. For bulk DNA analysis, we extracted DNA using the Qiagen MagAttract kit as per manufacturer protocol, using 20 mg starting material. MSA1 long-read WGS data were available previously^26^, and MSA2 and control ONT long-read samples were processed at the Baylor College of Medicine (BCM) core using the same protocol as MSA1. Sample MSA2 had an average size of 8.7 kb and did not require shearing. The control sample had an average size of 52.6 kb and was sheared using g-tubes (Covaris). Three µg of DNA was added to the g-tube and centrifuged at 3800 rpm (4-passes) to achieve an average sheared size of 15–20 kb.

#### Bulk sequencing

Library preparation and sequencing for bulk ONT sequencing of MSA2 and control brain cortex were performed at the BCM core; data from MSA1 were already available^35^. For the three tissue samples, the genomic DNA was extracted and quantified using the Lunatic UV/Vis spectrophotometer (Unchained Labs), with fragment size analysis performed on the Agilent Femtopulse. The DNA was diluted to 60 ng/µL in nuclease-free water to a final volume of 50 µL. For shearing, 3 µg of diluted DNA was processed using Covaris g-tubes (Covaris 520079), with each sample passed four times through the tube orifice via centrifugation at 3,800 rpm. Following shearing, the DNA was purified using AMPure XP beads (Beckman Coulter) and size-selected on the PippinHT instrument (Sage Science) using the 6–10 kb High-Pass definition with a minimum size threshold of 10 kb. The PippinHT cassette was loaded and eluted according to the manufacturer’s instructions, after which the eluted sample underwent another AMPure XP bead purification and was resuspended in 50 µL of nuclease-free water. To assess fragment size, 1 µL of the eluate was analyzed on the Agilent 2100 Bioanalyzer using the DNA 12000 chip, yielding an expected average size of 15–20 kb. For libraries targeting larger inserts (25–30 kb), modifications were implemented: 7.5 µg of genomic DNA at 80 ng/µL was sheared using g-tubes with only two passes at 3800 rpm, and size selection was performed on the PippinHT using the 15–20 kb High-Pass definition with a 20 kb threshold. Subsequent steps remained identical. Subsequently, for both library types, 48 µL of the purified fragments were used as input for the ONT SQK-LSK114 library preparation. End repair/damage repair and adapter ligation were performed per the manufacturer’s protocol. Post-ligation, libraries were purified with 1X AMPure XP beads, washed with Long Fragment Buffer, and eluted in 27 µL of ONT elution buffer. Quantification was performed using the Qubit dsDNA Broad Range assay (Thermo Fisher Scientific). Libraries were sequenced on R10.4.1 flow cells (FLO-PRO114M, ONT) using a PromethION 24 device with default settings. Approximately 150–200 ng of library (15–20 kb average insert size) was loaded per flow cell. Sequencing was conducted for 80 hours in MinKnow v22.10.7, with on-board basecalling using the super-accurate (SUP) model. If flow cell blockage occurred, a nuclease wash was performed, followed by reloading of 150–200 ng of library.

### Processing of HG002 sample

Data were aligned to GRCh37/38 reference genomes using minimap2 v2.22 (RRID:SCR_018550; https://github.com/lh3/minimap2)^37^ and converted to binary format using Samtools v1.19.2 (RRID:SCR_002105; https://github.com/samtools/)^38^. Mosdepth v0.3.3 (RRID:SCR_018929; https://github.com/brentp/mosdepth)^39^ was used to determine the per-base coverage for each sample. Clair3 v1.0.0 (RRID:SCR_026063; https://github.com/HKU-BAL/Clair3)^40^ was used to obtain SNV calls for each sample. Calls were split into substitutions and small In/Dels, which were further filtered for those encompassing 5 or more reads of coverage using BEDTools v2.30.0 (RRID:SCR_006646; https://github.com/arq5x/bedtools2)^41^, with at least 3 reads supporting each call and “PASS” in the VCF filter field. Results were compared to HG002 GiaB reference SNV calls using RTGtools v3.12.1 (https://github.com/RealTimeGenomics/rtg-tools)^42^. The high genotype quality “false-positive” calls were filtered using Bcftools, filtering for GQ ≥ 20. The SV calls were compared to HG002 ONT sequencing calls obtained from Oxford Nanopore Technologies, by running Sniffles v.2.2 (RRID:SCR_017619; https://github.com/fritzsedlazeck/Sniffles)^26^ with available reference genomes (GRCh37 and GRCh38 respectively). To remove calls presumably resulting from chimeric reads, we ignored all duplications and inversions, and applied similar chimera filtering as to single-cell samples (**see** Supplementary text Section 2, Fig. 70). Resulting SV calls were filtered using PASS filter in the *vcf* file; sequences were selected for those longer than 50 bp, supported by 3 or more reads in locations covered by at least 5 reads. The detected structural variants were compared to a HG002 GiaB SV dataset using Truvari benchmark v4.0.0 (RRID unavailable, https://github.com/ACEnglish/truvari)^43^. Custom scripts were used to annotate and summarize results. Results were traced to their original cells, and the proportion of single-cell and multi-cell variants absent in bulk tissue was calculated.

### Long-read ONT analysis of human brain samples

The single-cell and corresponding bulk samples were analyzed similarly to HG002 sample. Reads were aligned to GRCh38/Hg38 reference genome using minimap2 and converted to binary format using Samtools (software versions same as above). Initial sequencing metrics were assessed with Cramino v0.13.1 (RRID unavailable, https://github.com/wdecoster/cramino)^44^ for read length distribution, see Supplementary Figs 42-47). Binary files were merged within the same experiment and tissue type with samtools. To reduce chimera signal, initial reads were filtered using methodology described in Supplementary text Section. 2. Mosdepth v0.3.3 (RRID:SCR_018929; https://github.com/brentp/mosdepth)^39^ was used to determine the per-base coverage for each sample.

In this study, we utilized two different library preparations for all cells: T7 debranching, the standard method to remove displaced strands created by MDA; and the PCR rapid barcoding protocol (RBP), which should eliminate all side chains by creating linear molecules, at the possible risk of smaller read lengths impacting SV detection.

As T7 debranching performed on a MinION device for MSA1 yielded only 2.64 Gb, T7 debranching for MSA2 and control benefited from the high sequencing throughput of the PromethION device (Supplementary data, Table 1). Additionally, we used RBP protocol, which led to high yield from MinION - over 7.4 * 10^10^ bases on (~62.5 million reads) and 10.6 * 10^10^ bases on PromethION T7 (~81.5 million reads) across the 6 cells of each brain. The average read N50 was 2.79 kb for both MinION RBP and Promethion T7 library samples, with minor differences across individual experiments, although the T7 provided a wider range of read sizes, with 4.5 million reads > 3 kb (MSA2; ~250 k reads for MSA1 T7, 5.9 million for control), and some as long as 300 kb (Supplementary Figs 42-47). Due to low yield of the MSA1 T7 MinION and the lack of additional material for T7 resequencing, it was excluded from further comparisons. Merging single-cell data from the same brain region produced more uniform coverage in each sample, while retaining the ability to trace individual variants to cells they originate from. In MSA2 and control, where we have T7 PromethION and RBP MinION, the PromethION as expected provided increased coverage to ≥5x depth (in MSA2 MinION RBP—36.4% and PromethION T7 —45.7%, and in control 15.0% and 21.7%, respectively. For MSA1 MinION RBP produced 35.4% of the genome). In contrast, Illumina MDA sequencing run of MSA1 covers 61.1% of the reference genome, MSA2 covers 62.5% while control brain covers 47.4% at ≥5x (total Illumina ≥1x coverage: 81.8% for MSA1, 86.3% for MSA2, 72.6% for control brain). Overall, the PromethION device combined with T7 debranching delivers largest yield of reads with high, sustained N50 of over 2.8 kb, covering up to 46% of the Human genome with high-quality assembly from single-cell data, and we recommend it as it captures the most signal within the tested pool of single cells.

### Detection of variants across analyzed brain samples

Alignments (both ONT and Illumina) were analysed for SNVs using Clair3 v1.0.0 (RRID unavailable; https://github.com/HKU-BAL/Clair3)^40^ was used to obtain SNV calls for each of the samples. SNV calls were merged with GLNexus, version 1.4.1 (RRID unavailable, https://github.com/dnanexus-rnd/GLnexus)^45^. Calls were split into substitutions and small In/Dels, which were further filtered for those encompassing 5 or more reads of coverage, with at least 3 reads supporting each call and “PASS” in the VCF filter field. The high genotype quality ONT-only single-cell calls were filtered using Bcftools, filtering for GQ ≥ 20. Single-cell results were compared with their bulk counterparts using custom scripts. Results were also compared to Illumina single-cell, as well as bulk SNV calls using RTGtools v3.12.1 (https://github.com/RealTimeGenomics/rtg-tools)^42^.

Next, alignments were analysed for SVs using Sniffles v2.2^26^. Initial SV calls were summarized with Python scripts to characterize signal from all types of SVs (insertions, deletions, duplications, inversions, and breakends/translocations). To avoid chimeras, we filtered inversions and duplications^46^ (summary for these can be found in Supplementary data, Table 31), as well as applied other filters described in Supplementary text, Section 2 (For sizes of initially called inversions and duplications, refer to Supplementary Figs 48–53).

All SV filtering steps were collated into a single script that obtained support and coverage information from the VCF tags. The individual single-cell samples from RBP MinION and T7 PromethION experiments were summarized with that script, and ratios of INS/DEL were compared between single-cell samples. Variants detected in CD8+ T-cell^9^ were analysed with a custom set of scripts, reflecting the steps applied to MSA brain samples. SVs were split into insertions and deletions, and for each merged experiment, they were compared with results from corresponding bulk samples. Based on the resulting numbers, we determined the variants private to single cells, variants shared between single cell samples, and corresponding bulk as well as bulk-only variants (limited to regions of ≥5x coverage recovered from corresponding single cells). We calculated the proportions of bulk-only mosaic variants and variants with 0/1 bulk-only genotype with custom scripts (for variant size distribution refer to Supplementary Figs 54–65**, for coverage see** Supplementary Figs 66–69). To cross-compare the detected variants with short-read Illumina scWGS samples, we used Illumina Manta v1.6.0 (RRID: SCR_022997, https://github.com/Illumina/manta)^47^ on data from each of the 3 brains (MSA1: 15 cells, MSA2: 12, control: 7). Using SVtyper v0.7.1 (RRID unavailable, https://github.com/hall-lab/svtyper)^48^, we determined how many LR-scWGS reported variants overlapped with SR-scWGS samples.

### Annotation of brain variants

Sequences of detected insertions and deletions were extracted from the VCF file and corresponding reference genome with custom scripts into FASTA format. RepeatMasker v4.1.5 (RRID:SCR_012954, https://www.repeatmasker.org/)^49^ was used to annotate the recovered variant sequences for presence of retrotransposons. TE annotation of brain samples was analyzed using custom Python scripts to define the variants containing most abundant and active transposon families, SINE/Alu and LINE/L1. The purpose of these was to parse RepeatMasker output and select results containing target TEs, track variant counts, and provide statistics on variants/elements as an output. The scripts are available via github repository, linked below. To collapse results into TE elements and determine the completeness of detected transposons, a “One code to find them all” v1.0 tool was used (RRID unavailable, https://doua.prabi.fr/software/one-code-to-find-them-all)^50^, followed by custom scripts to summarize results. Furthermore, we analyzed the proportion of TE insertions into preexisting repeated elements of the same family. SINE/Alu elements’ subfamily was determined from the annotation, as well as the actual number of fragmented elements/elements taking more than 80% of the entire insertion. Next, we detected the proportion of deletions containing SINE/Alu or LINE elements and deletions in which the same type of transposable element can be found on the opposite breakpoints of the variant. The distribution of deletions containing SINE/LINE TEs was used as template for permutation test; BEDTools shuffle algorithm was utilized to simulate single-cell deletion distribution across the reference Hg38 genome, which was compared to brain samples results’ with *Z*-test. RepeatMasker results were filtered for Alu subfamilies using custom scripts.

To determine the propensity of variants for mutating known genes, we used AnnotSV version 3.4.2 (RRID unavailable, https://www.lbgi.fr/AnnotSV/)^51^ to find the overlap between INS/DEL and gene locations, particularly with a list of genes involved with neurodegeneration compiled from literature. Furthermore, we used bcftools intersect version 1.19 (RRID:SCR_002105; https://github.com/samtools/bcftools)^38^ to find the overlap between variants (SVs and SNVs/InDels) and known exon locations extracted from Gencode v45 (RRID:SCR_014966; https://ftp.ebi.ac.uk/pub/databases/gencode/Gencode_human/)^52^. Similarly to TEs, we performed permutation tests using locations of deletions and insertions, as well as single-cell SNVs/InDels, in order to determine the correlation with reference exons with *Z*-test. We determined a number of genes related with MSA in the literature, and used scripts to filter the AnnotSV/Gencode results for genes affected by SVs and related to MSA. To determine population frequency of the variants detected in single cells and bulk simultaneously, we used stix-suite 1.0.1 (RRID unavailable, https://github.com/ryanlayer/stix?tab=readme-ov-file#stix-suite^53^; the processed population data used to generate Fig. 1e is provided in Supplementary data, Table 32). Visual inspection of detected variants as well as overlap with genomic annotations was performed using IGV version 2.18.2 (RRID: SCR_011793, http://www.broadinstitute.org/igv/)^54^.

### Statistics and reproducibility

All statistical analyses and data processing were performed using established tools, as detailed in the Methods. Variant calling pipelines included filtering thresholds for coverage and quality to ensure reproducibility. For SNVs and small InDels, only variants with coverage ≥5 reads and ≥3 supporting reads, and that passed the VCF “PASS” filter, were retained. Genotype quality filtering (GQ ≥ 20) was applied to remove likely false positives. For structural variants (SVs), only variants ≥50 bp supported by ≥3 reads and located in regions covered by at least 5 reads were considered. To control for chimeric artifacts, inversions and duplications were excluded from most analyses, and additional filtering was applied as described in Supplementary text, Section 2.

Custom Python scripts were used to summarize results and annotate overlap with reference data, including RepeatMasker annotations, gene and exon features, and known population SVs. For statistical assessment of insertion and deletion enrichment within genomic features, *Z*-tests were applied to compare observed values to distributions generated from 10,000 permutations using BEDTools’ shuffle function. Significance was defined as *p* ≤ 0.05.

Sample sizes are as follows: single-cell sequencing was performed on *n* = 6 cells per studied brain (two MSA cases and one control), with technical variation introduced via two library preparations per individual (T7 and RBP), with equivalent bulk DNA sequenced for each studied brain. All sequencing datasets were quality-controlled using standardized tools (e.g., mosdepth, cramino, minimap2). Distinct single nuclei were isolated and processed independently for each donor and for both library preparation methods, and are treated as independent single-cell observations.

All analyses were repeated for each brain individually. Intermediate files (VCF, BAM) were tracked, and reproducibility of variant sets was assessed by comparison across individual cells, between single-cell and bulk, and against established reference datasets (e.g., HG002 GiaB).

### Reporting summary

Further information on research design is available in the Nature Portfolio Reporting Summary linked to this article.

## Supplementary information

Supplementary information

Description of additional supplementary file

Supplementary data

Reporting summary

## Data Availability

All single-cell ONT data, and bulk ONT data for MSA2 and control, are available at the European Genome-Phenome Archive (EGA) (EGAS50000001156). MSA1 bulk ONT data were previously deposited in the Sequence Read Archive (SRA) bioproject ID PRJNA985263. Illumina bulk WGS from MSA brains have been deposited in dbGaP (accession #: phs001963.v3.p1) under the genome IDs MSA00915 (for MSA1_CingCx) and MSA00911 (for MSA2_CingCx). GIAB HG002 benchmark data links: https://www.nist.gov/programs-projects/genome-bottlehttps://ftp-trace.ncbi.nlm.nih.gov/ReferenceSamples/giab/release/AshkenazimTrio/HG002_NA24385_son/latest/https://ftp-trace.ncbi.nlm.nih.gov/ReferenceSamples/giab/release/AshkenazimTrio/HG002_NA24385_son/CMRG_v1.00/ Numerical source data for analyses and calculations performed in this study can be found in Supplementary data 1 file. The data, code, protocols, and key lab materials used and generated in this study are listed in a Key Resource Table alongside their persistent identifiers in Supplementary data, Table 33.

## Code availability

For detailed methodology, including instructions for every step of the analysis and all custom code, please refer to the GitHub repository (https://github.com/michalizydo/scONT; 10.5281/zenodo.14194549)^55^.
