# Supplementary material for: Single cell long read whole genome sequencing reveals somatic transposon activity in human brain
Source: Commun Biol. 2025 Nov 20;8:1627. doi: 10.1038/s42003-025-08805-2 (PMC12635067; doi:10.1038/s42003-025-08805-2)
Supplement: Supplementary file 1 — Supplementary information [file 42003_2025_8805_MOESM1_ESM.pdf]

Supplementary Information

Single cell long read whole genome sequencing reveals somatic transposon activity in human brain

Michal B Izydorczyk, Ester Kalef-Ezra, Dominic W Horner, Xinchang Zheng, Nadine Holmes, Marco Toffoli, Zeliha Sahin, Yi Han, Heer H Mehta, Sonja W Scholz, Clifton L Dalgard, Donna M Muzny, Adam Ameur, Fritz J Sedlazeck, Christos Proukakis

Supplementary Figures

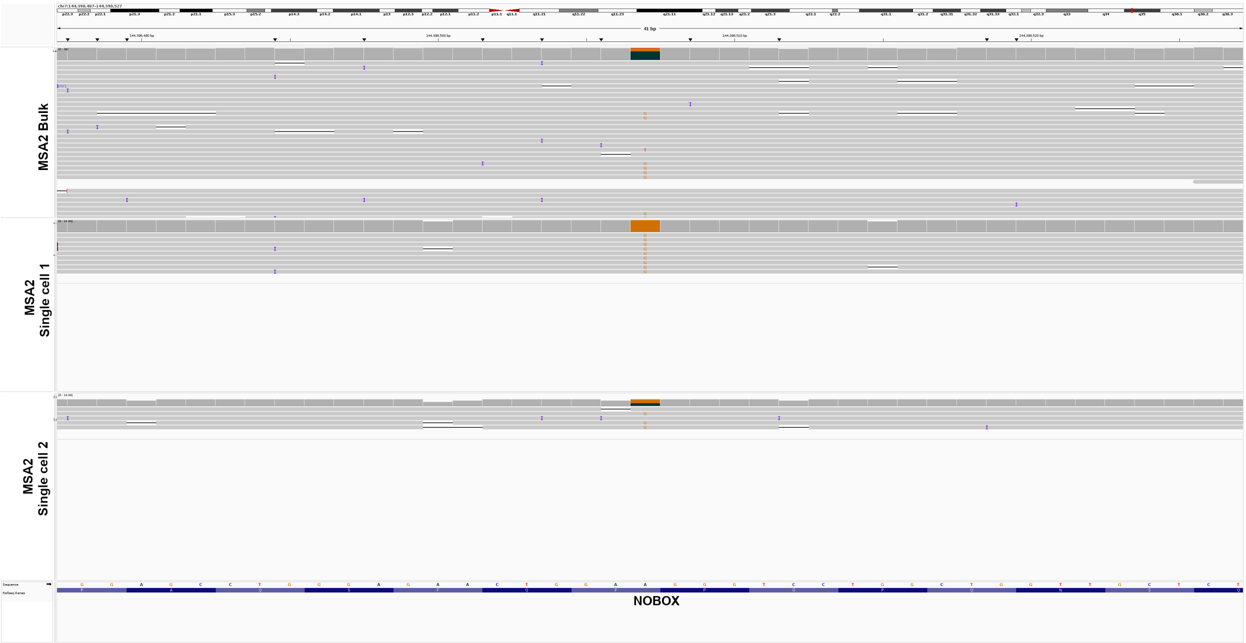

Supplementary Figure 1: Example of mosaic SNV detected in MSA2 bulk brain and corresponding single cells. For both single-cell samples MAPQ=60. The first single cell sample represent aggregated reads from 6 single cells sequenced with T7 and the second 6 single cells sequenced with RBP.

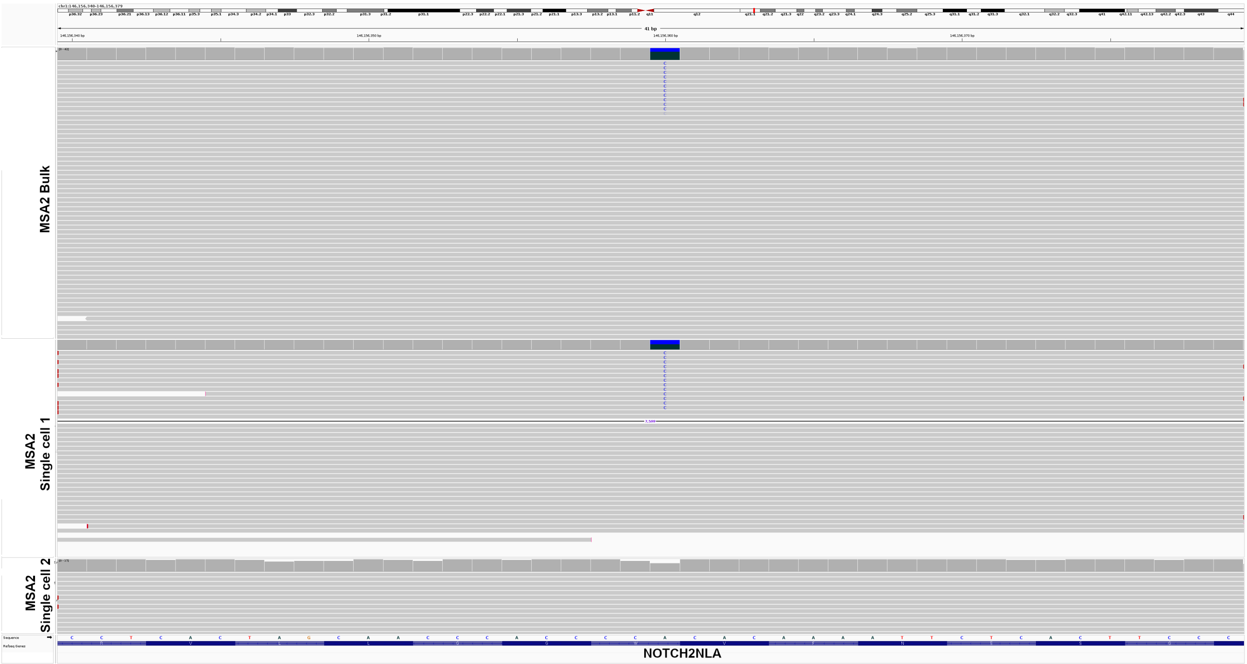

Supplementary Figure 2: Example of mosaic SNV detected in MSA2 bulk brain and corresponding single cells. MAPQ=32.9/23.6 [PromethION/MinION]. The first single cell sample represent aggregated reads from 6 single cells sequenced with T7 and the second 6 single cells sequenced with RBP.

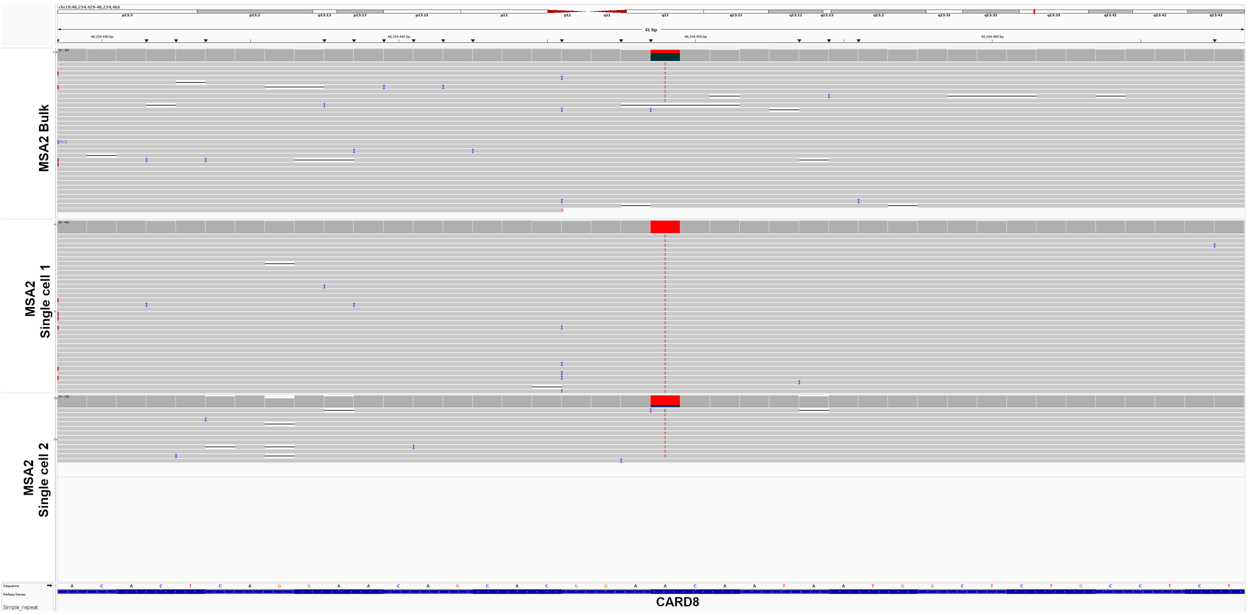

Supplementary Figure 3: Example of mosaic SNV detected in MSA2 bulk brain and corresponding single cells. For both single-cell samples MAPQ=60. The first single cell sample represent aggregated reads from 6 single cells sequenced with T7 and the second 6 single cells sequenced with RBP.

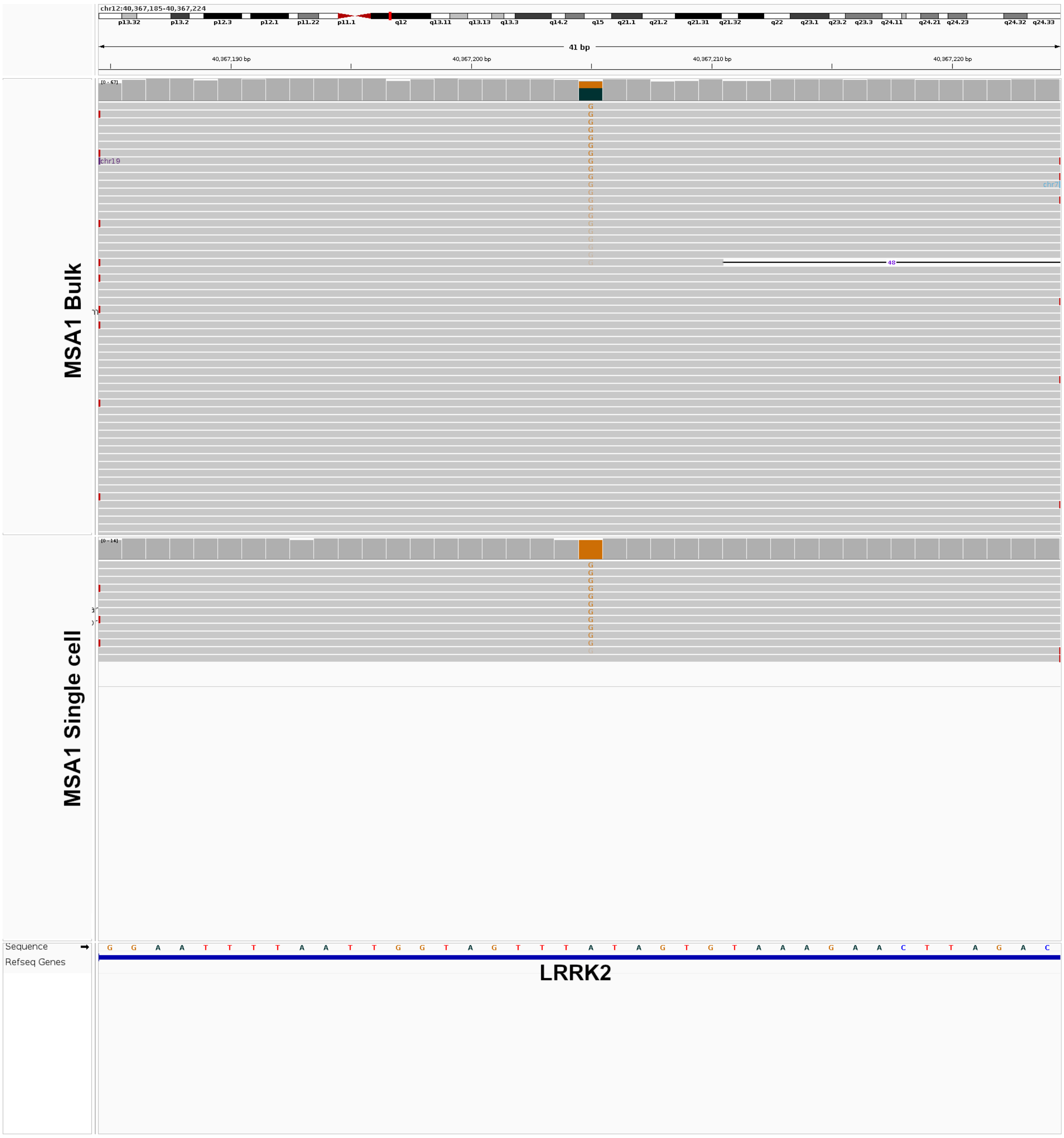

Supplementary Figure 4: Example of mosaic SNV detected in MSA1 bulk brain and corresponding single cells. For both single-cell samples MAPQ=60. The single cell sample represent aggregated reads from 6 single cells sequenced with RBP.

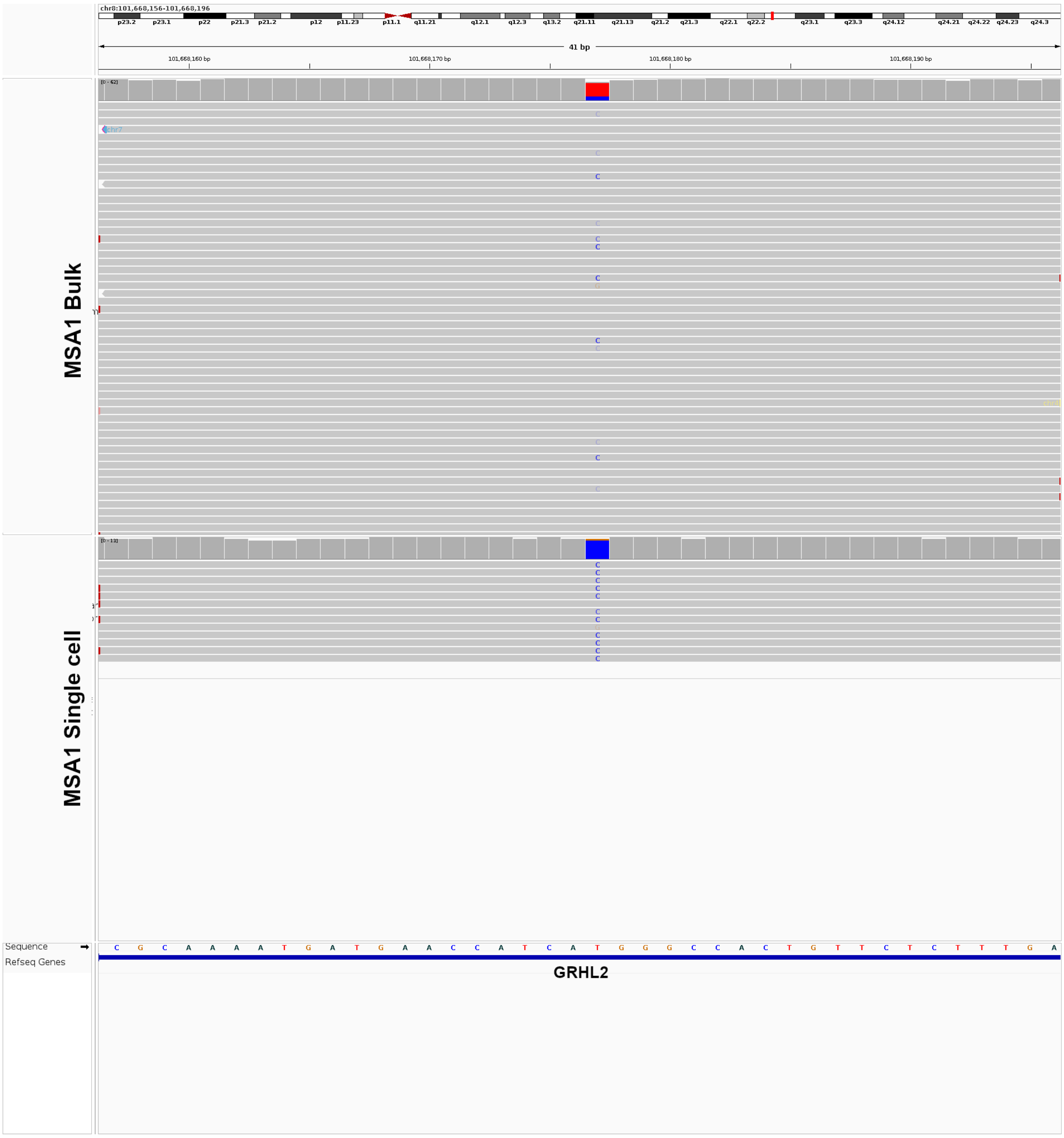

Supplementary Figure 5: Example of mosaic SNV detected in MSA1 bulk brain and corresponding single cells. For both single-cell samples MAPQ=60. The single cell sample represent aggregated reads from 6 single cells sequenced with RBP.

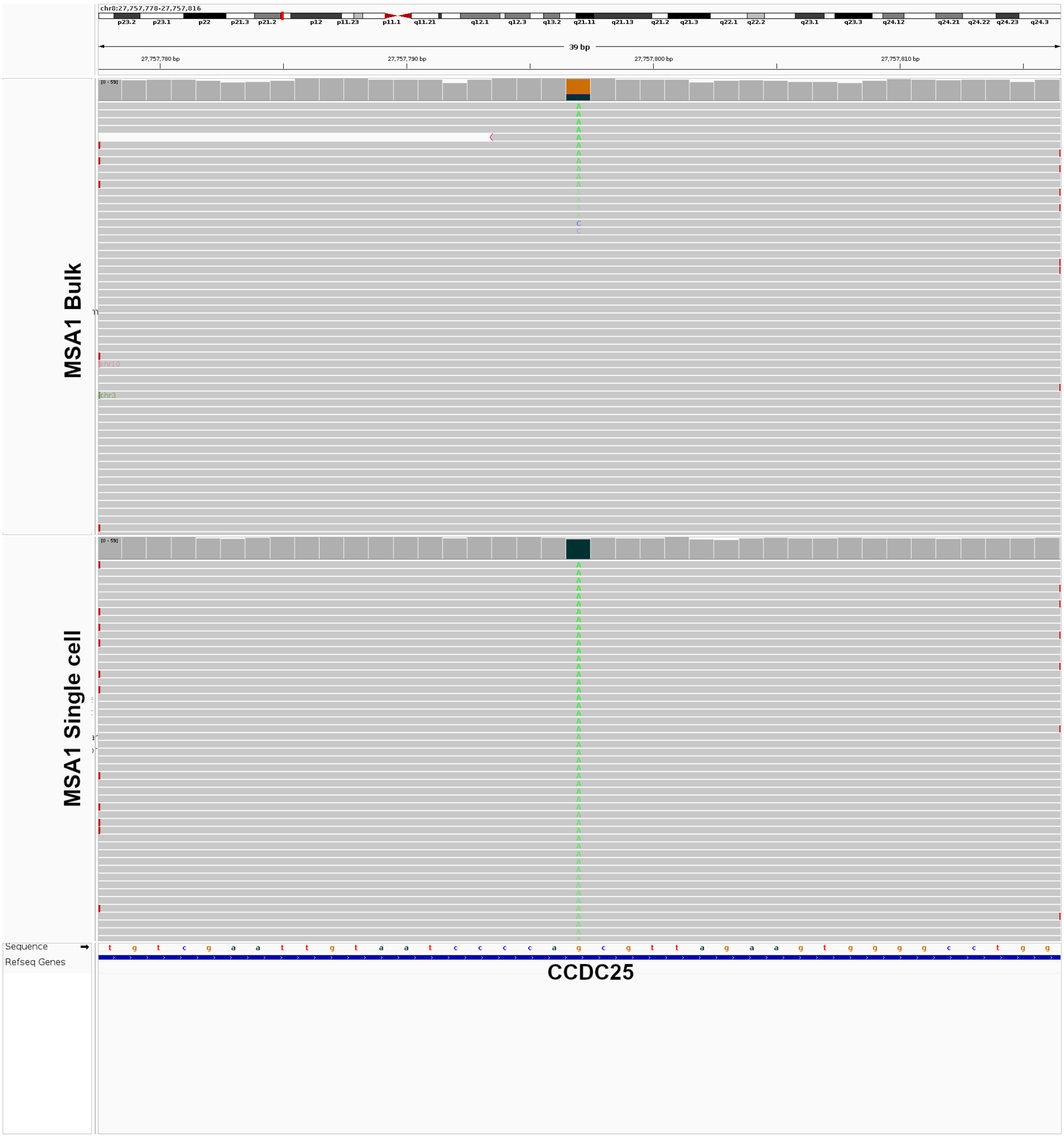

Supplementary Figure 6: Example of a mosaic SNV detected in MSA1 bulk brain and corresponding single cells. For both single-cell samples MAPQ=60. The single cell sample represent aggregated reads from 6 single cells sequenced with RBP.

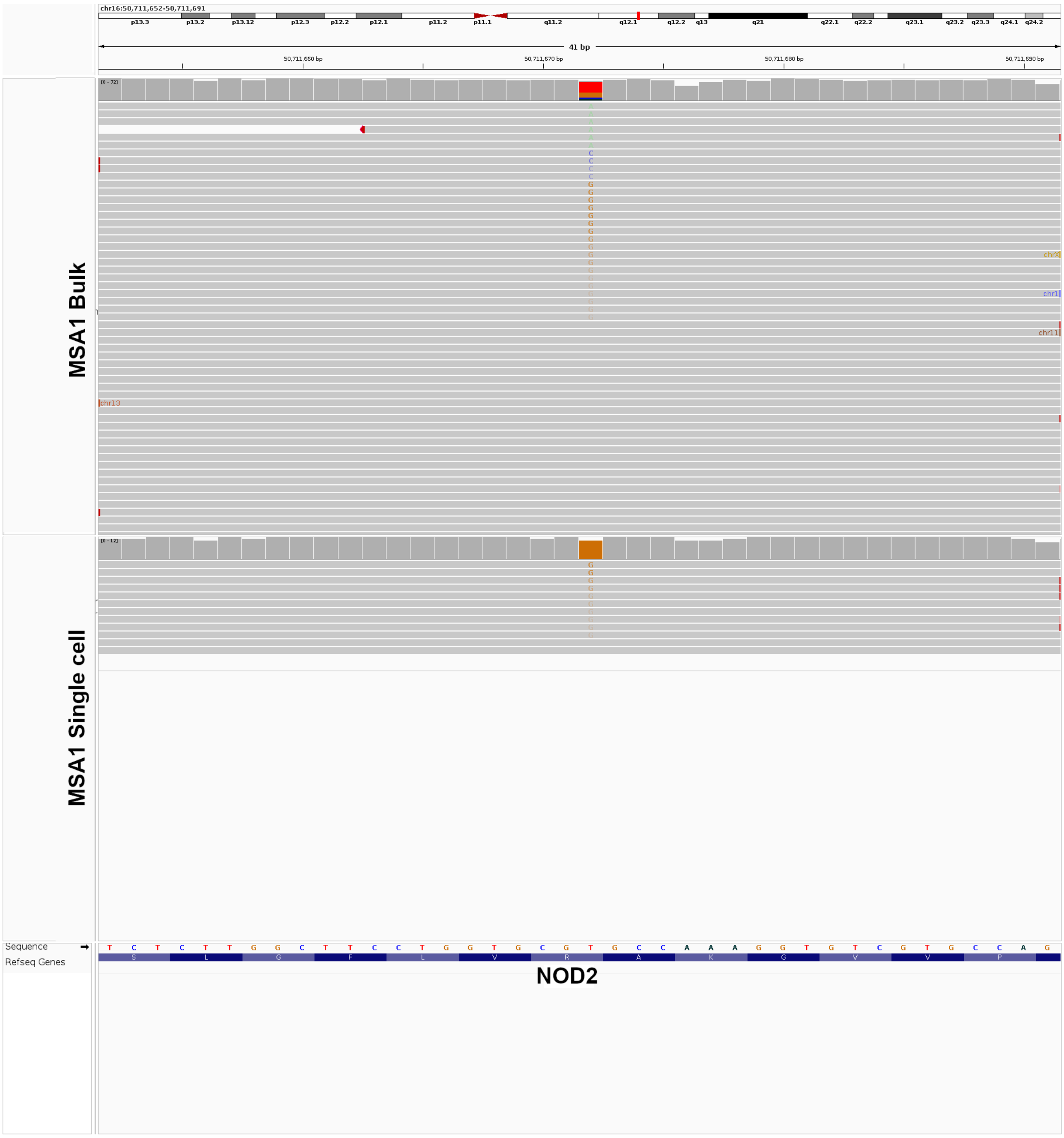

Supplementary Figure 7: Example of a mosaic SNV detected in MSA1 bulk brain and corresponding single cells. For both single-cell samples MAPQ=60. The single cell sample represent aggregated reads from 6 single cells sequenced with RBP

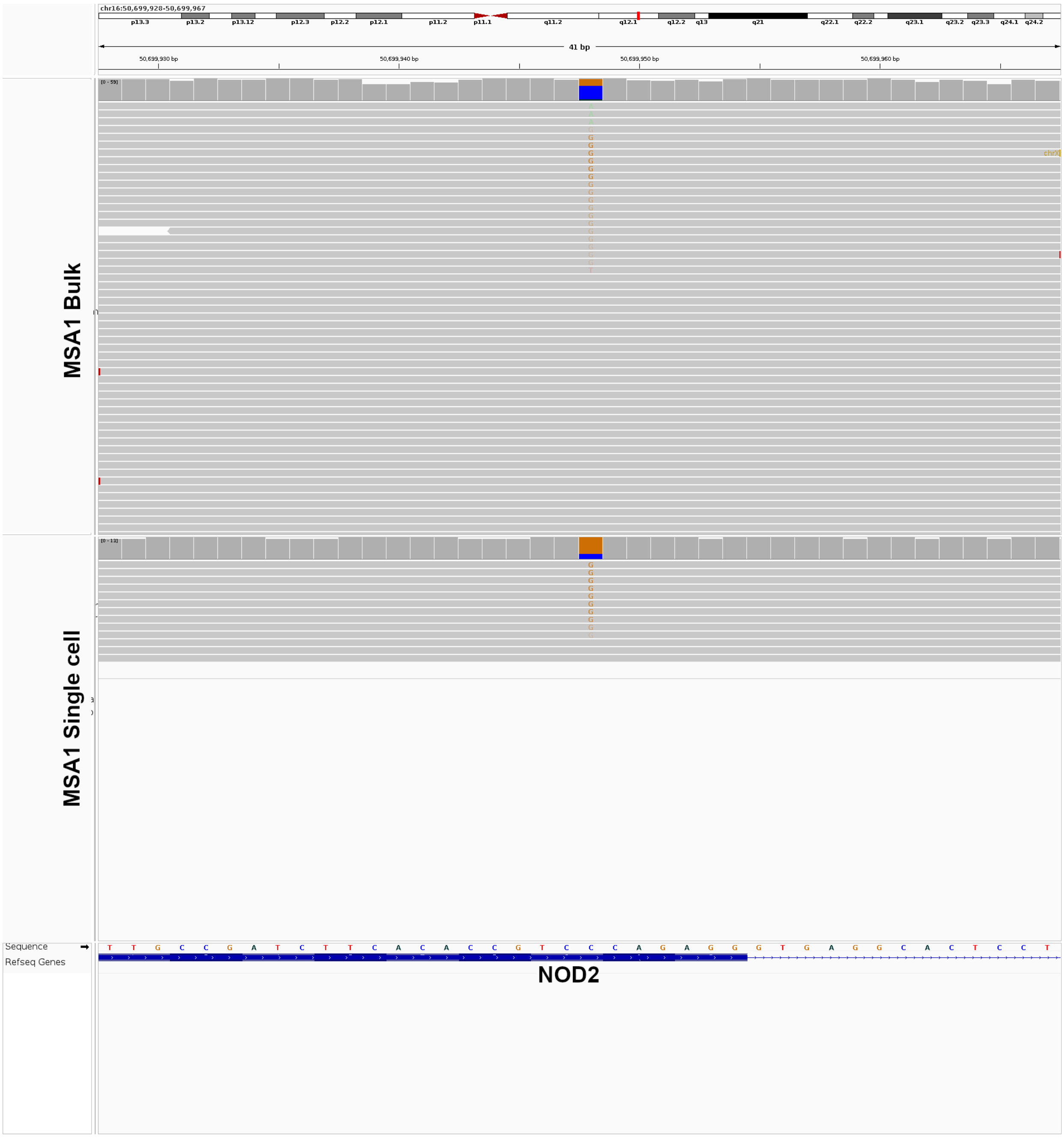

Supplementary Figure 8: Example of a mosaic SNV detected in MSA1 bulk brain and corresponding single cells. For both single-cell samples MAPQ=60. The single cell sample represent aggregated reads from 6 single cells sequenced with RBP.

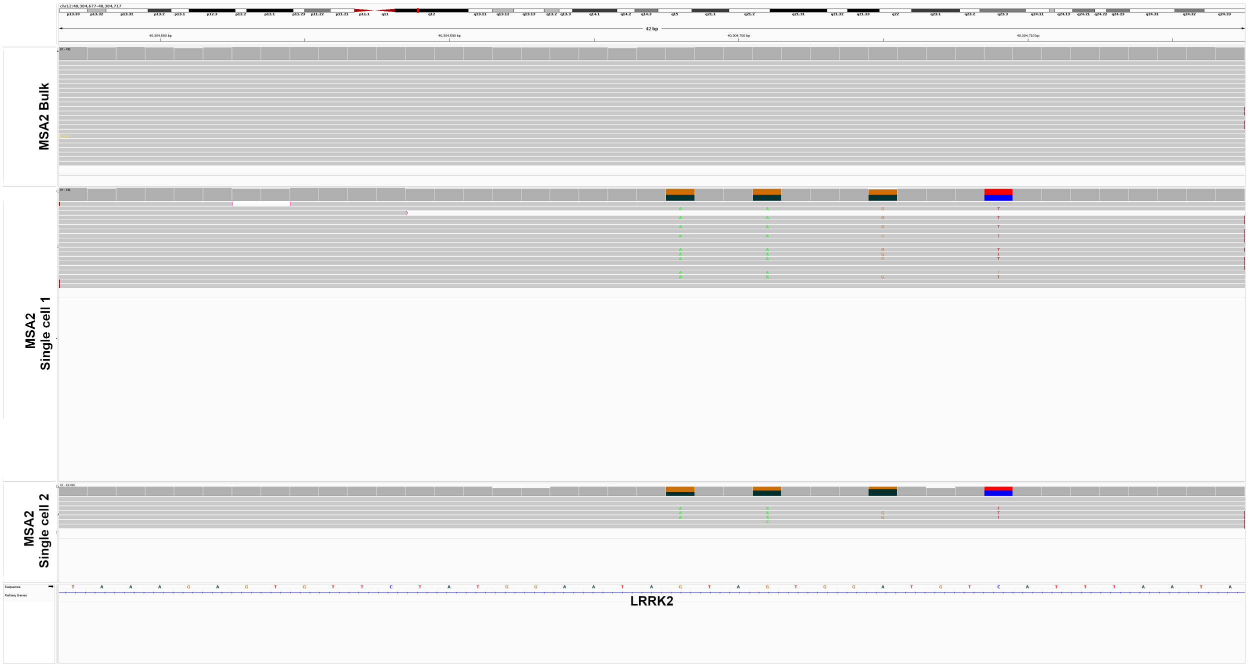

Supplementary Figure 9: Example of single-cell only SNV detected in MSA2 brain. MAPQ= 59.8889/60 [PromethION/MinION]. The first single cell sample represent aggregated reads from 6 single cells sequenced with T7 and the second 6 single cells sequenced with RBP.

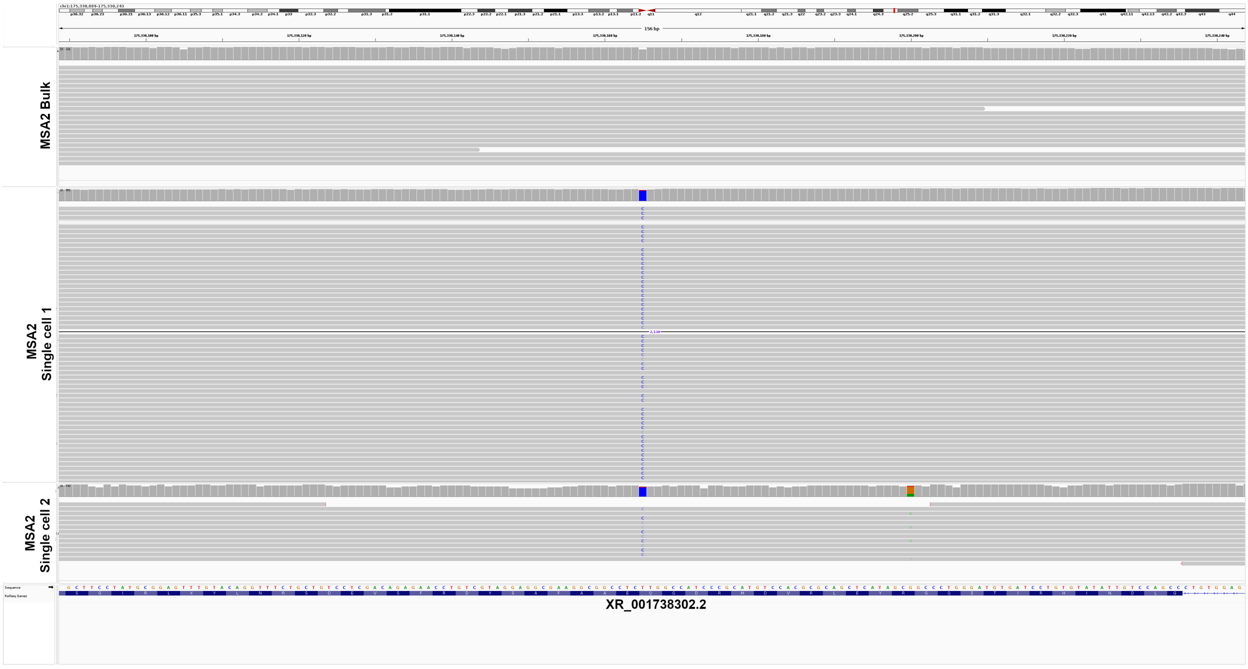

Supplementary Figure 10: Example of single-cell only SNV detected in MSA2 brain. MAPQ=59.4828/60 [PromethION/MinION]. The first single cell sample represent aggregated reads from 6 single cells sequenced with T7 and the second 6 single cells sequenced with RBP.

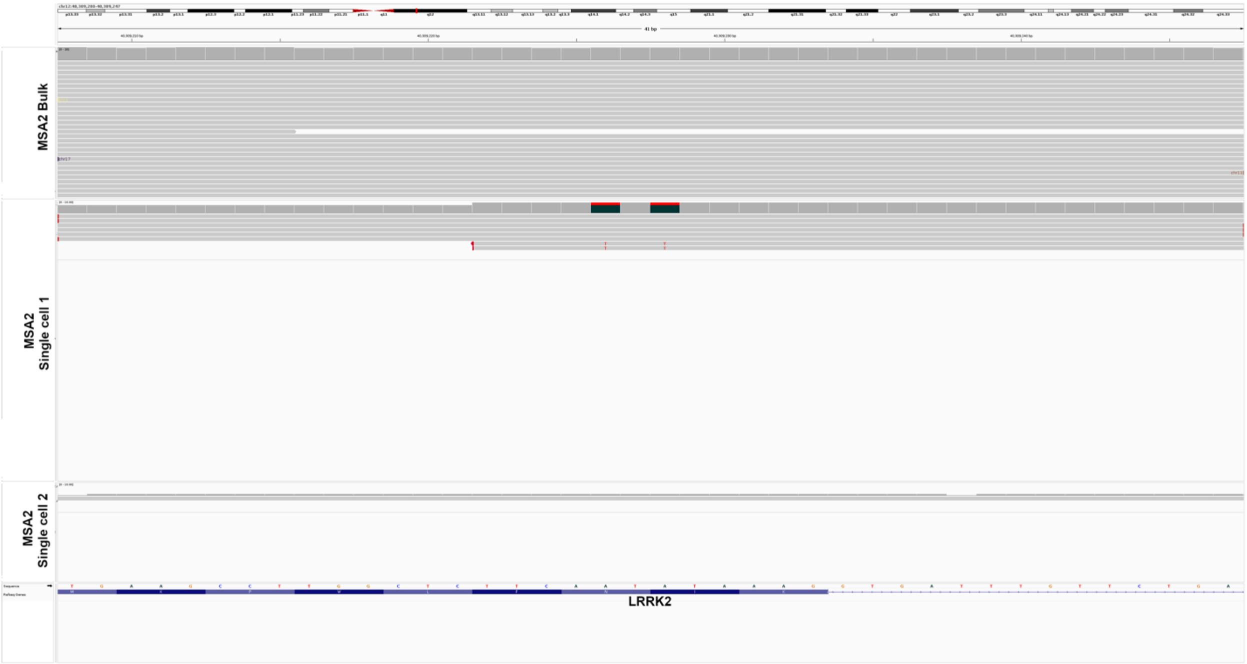

Supplementary Figure 11: Example of single-cell only SNV detected in MSA2 brain. For both single-cell samples MAPQ=60. The first single cell sample represent aggregated reads from 6 single cells sequenced with T7 and the second 6 single cells sequenced with RBP.

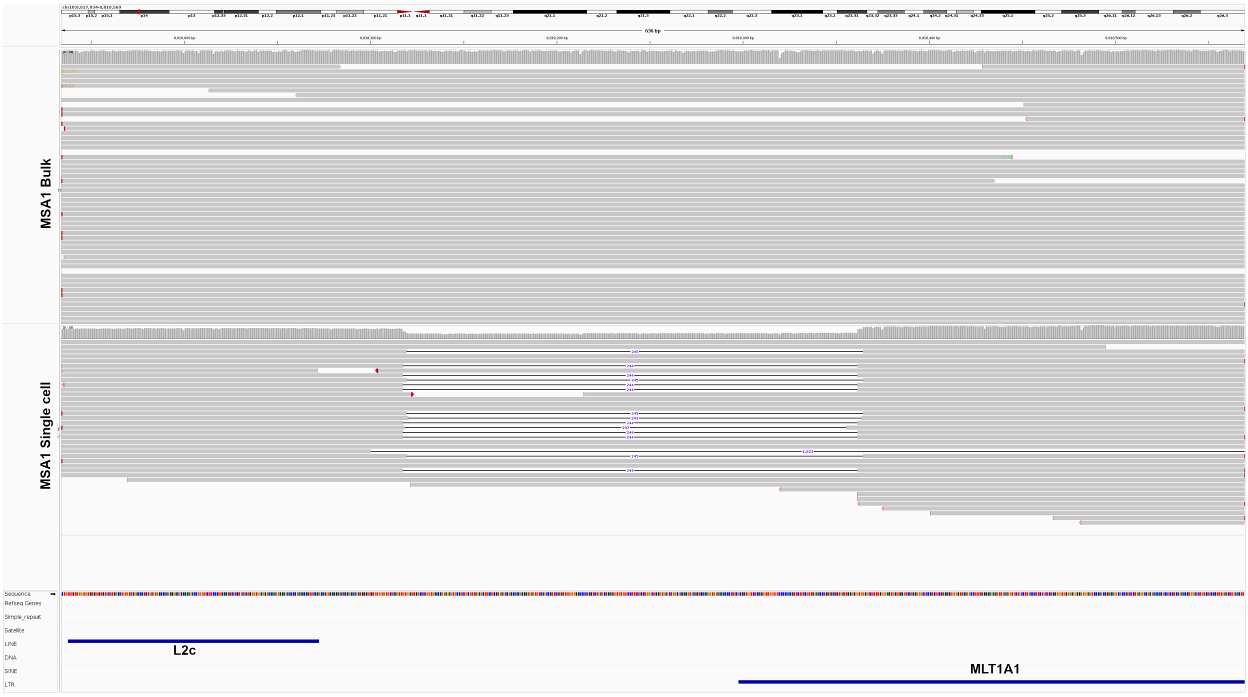

Supplementary Figure 12: Example of a single cell-specific deletion detected in MSA1 brain. For both single-cell samples MAPQ=60. The single cell sample represent aggregated reads from 6 single cells sequenced with RBP.

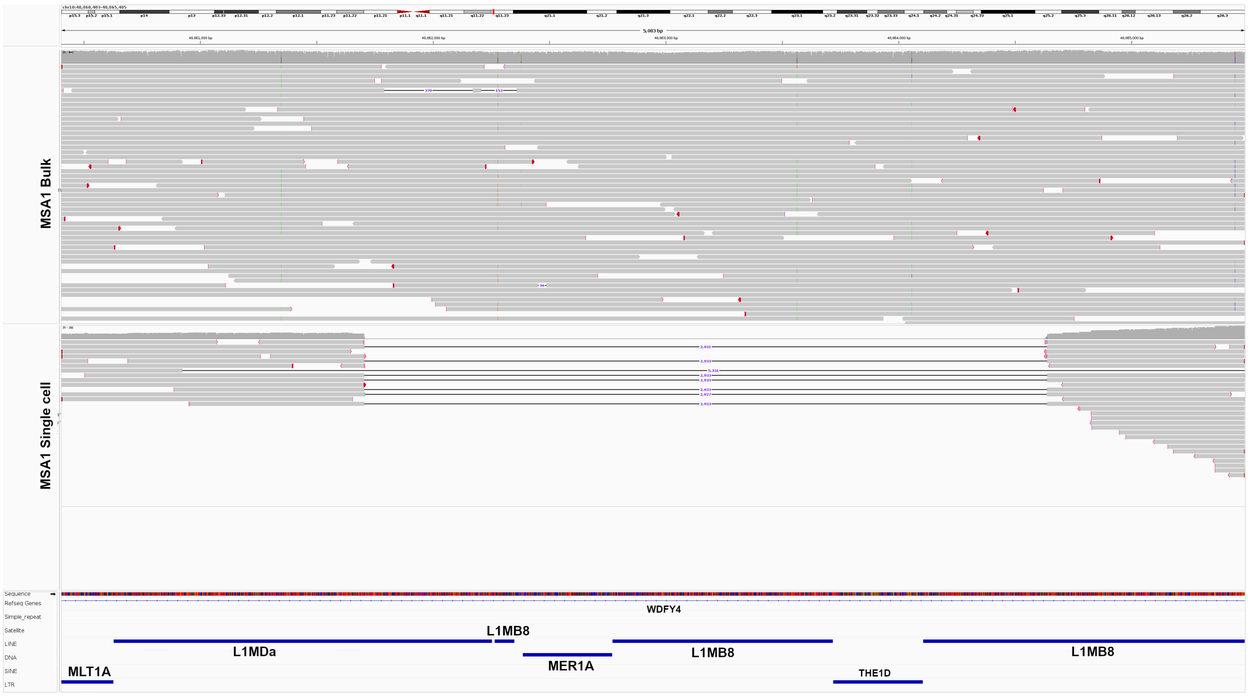

Supplementary Figure 13: Example of a single cell-specific deletion detected in MSA1 brain. MAPQ= 58.2857. The single cell sample represent aggregated reads from 6 single cells sequenced with RBP.

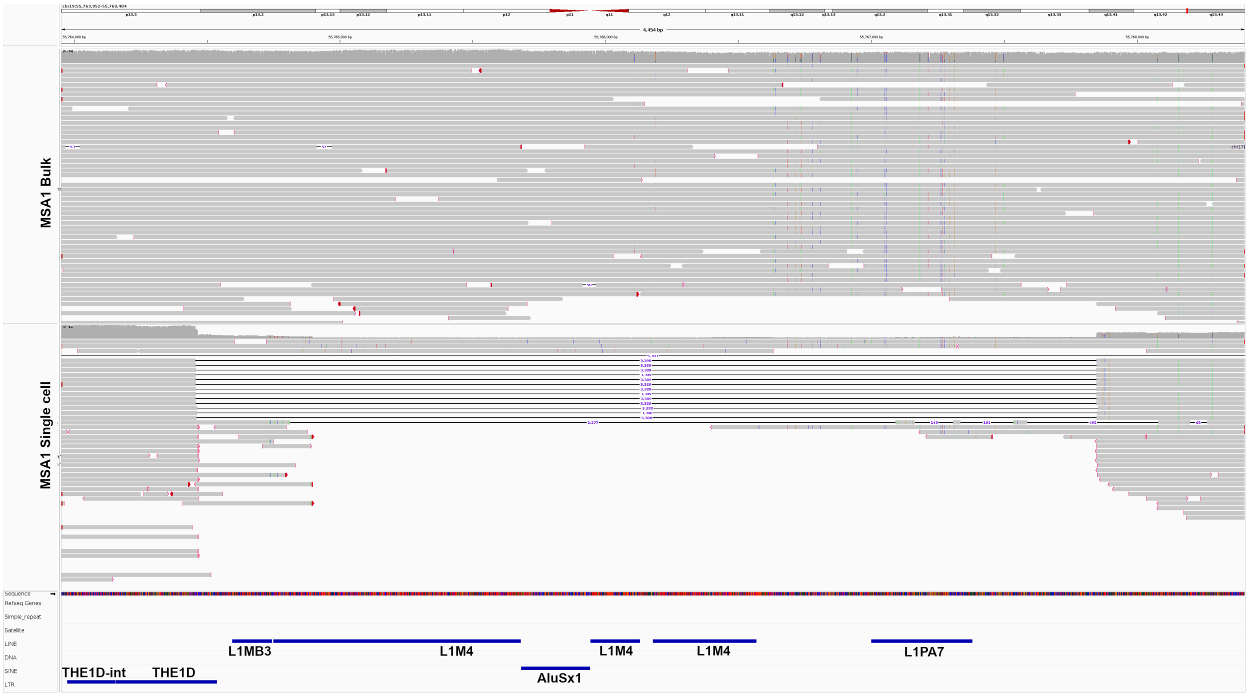

Supplementary Figure 14: Example of a single cell-specific deletion detected in MSA1 brain. MAPQ= 45.1915. The single cell sample represent aggregated reads from 6 single cells sequenced with RBP.

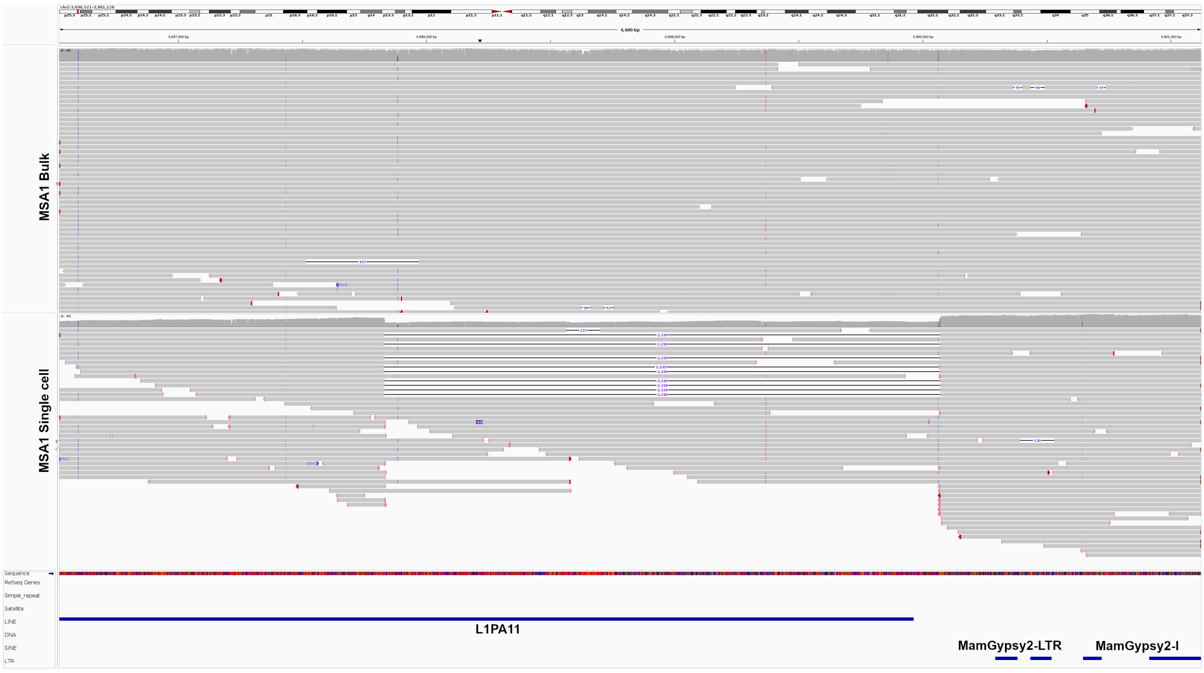

Supplementary Figure 15: Example of a single cell-specific deletion detected in MSA1 brain. MAPQ=60. The single cell sample represent aggregated reads from 6 single cells sequenced with RBP.

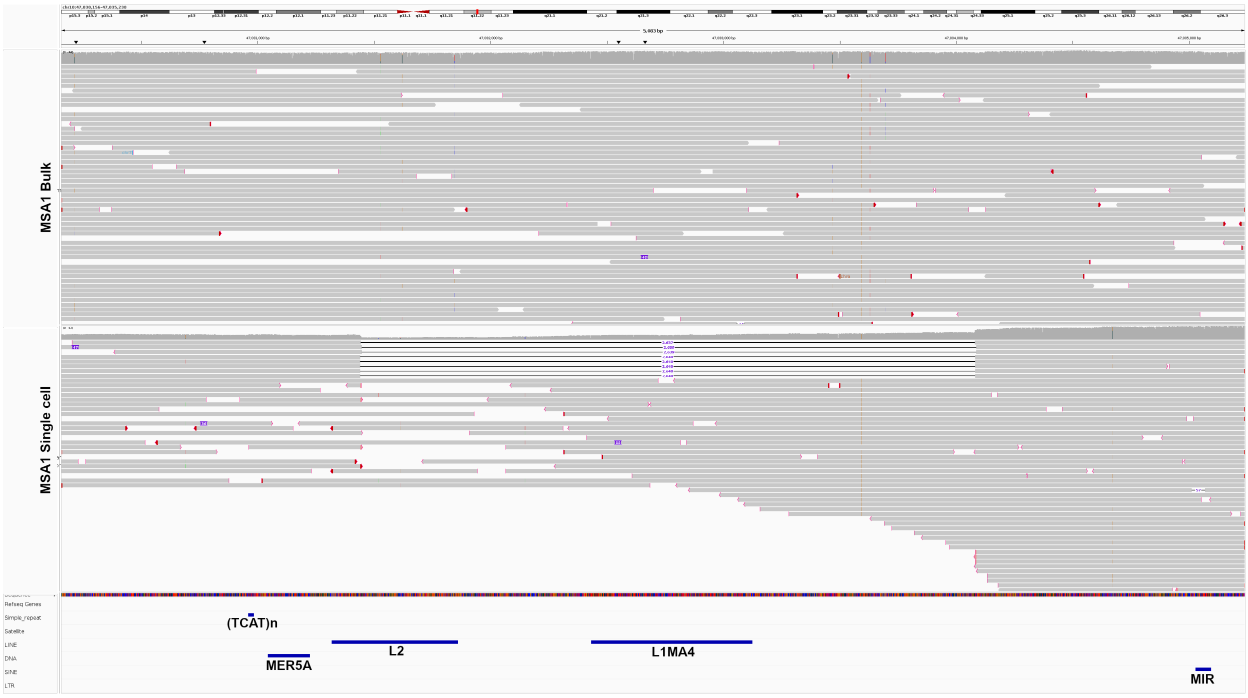

Supplementary Figure 16: Example of a single cell-specific deletion detected in MSA1 brain. MAPQ= 59.1111. The single cell sample represent aggregated reads from 6 single cells sequenced with RBP.

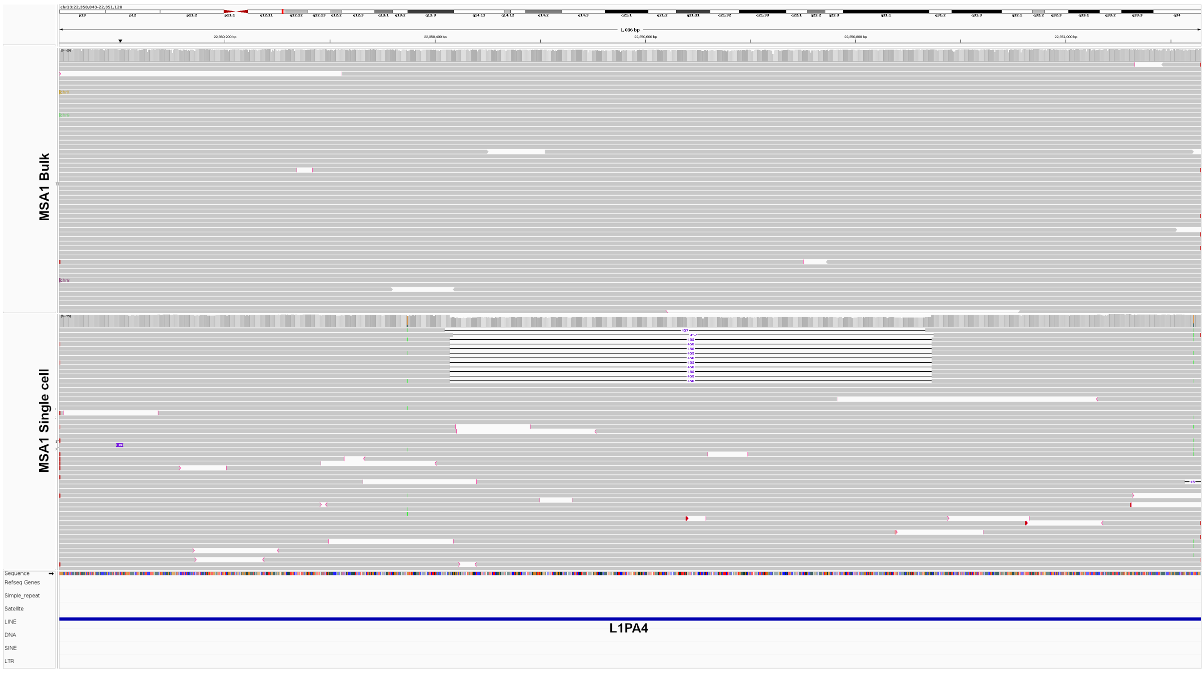

Supplementary Figure 17: Example of a single cell-specific deletion detected in MSA1 brain. MAPQ=57.25. The single cell sample represent aggregated reads from 6 single cells sequenced with RBP.

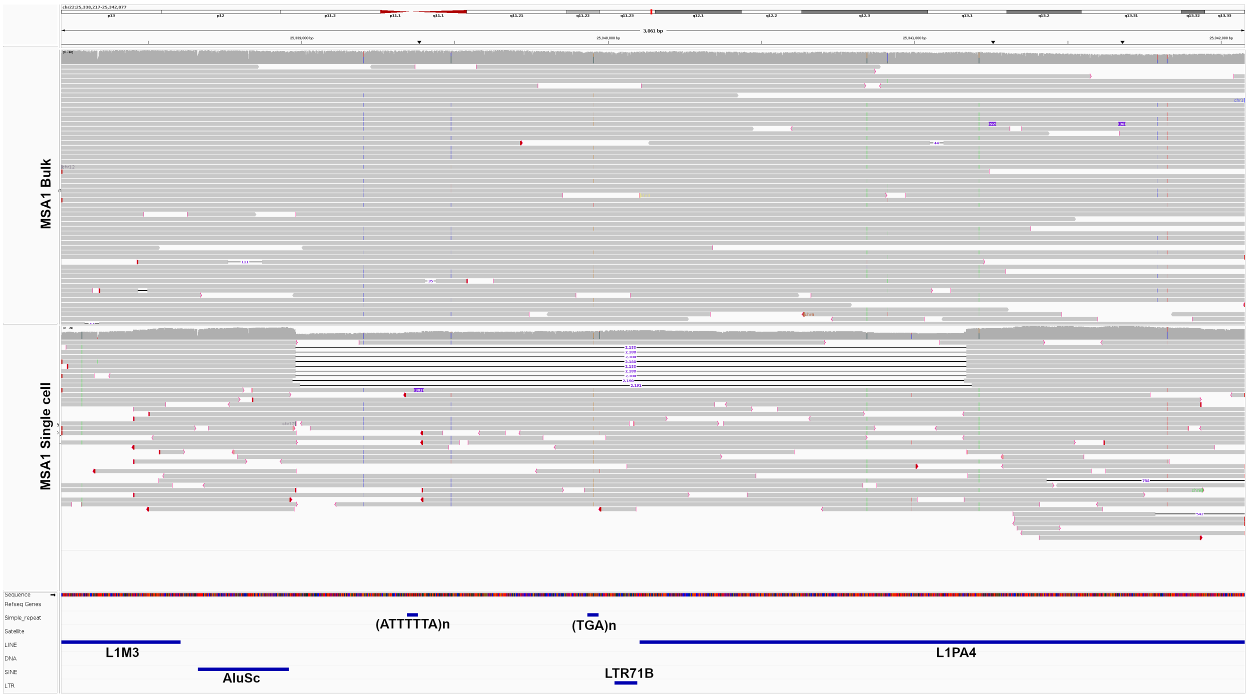

Supplementary Figure 18: Example of a single cell-specific deletion detected in MSA1 brain. MAPQ=57.8644. The single cell sample represent aggregated reads from 6 single cells sequenced with RBP.

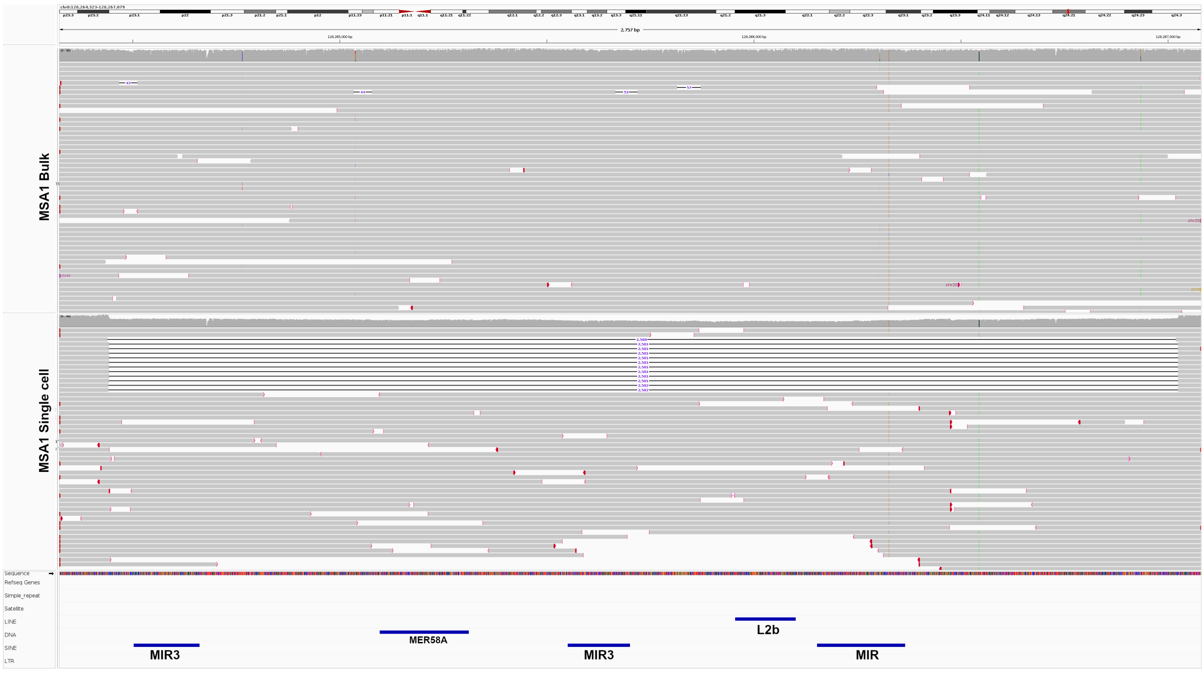

Supplementary Figure 19: Example of a single cell-specific deletion detected in MSA1 brain. MAPQ=60. The single cell sample represent aggregated reads from 6 single cells sequenced with RBP.

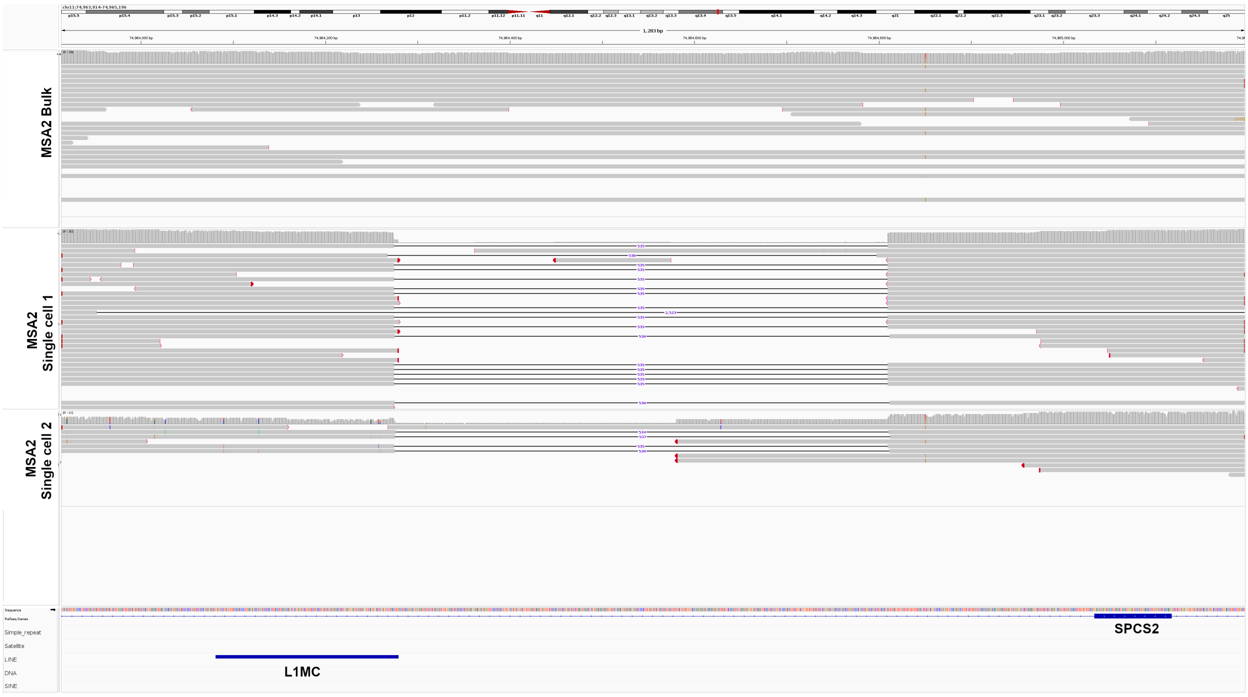

Supplementary Figure 20: Example of a single cell-specific deletion detected in MSA2 brain. For both single-cell samples MAPQ=60. The first single cell sample represent aggregated reads from 6 single cells sequenced with T7 and the second 6 single cells sequenced with RBP

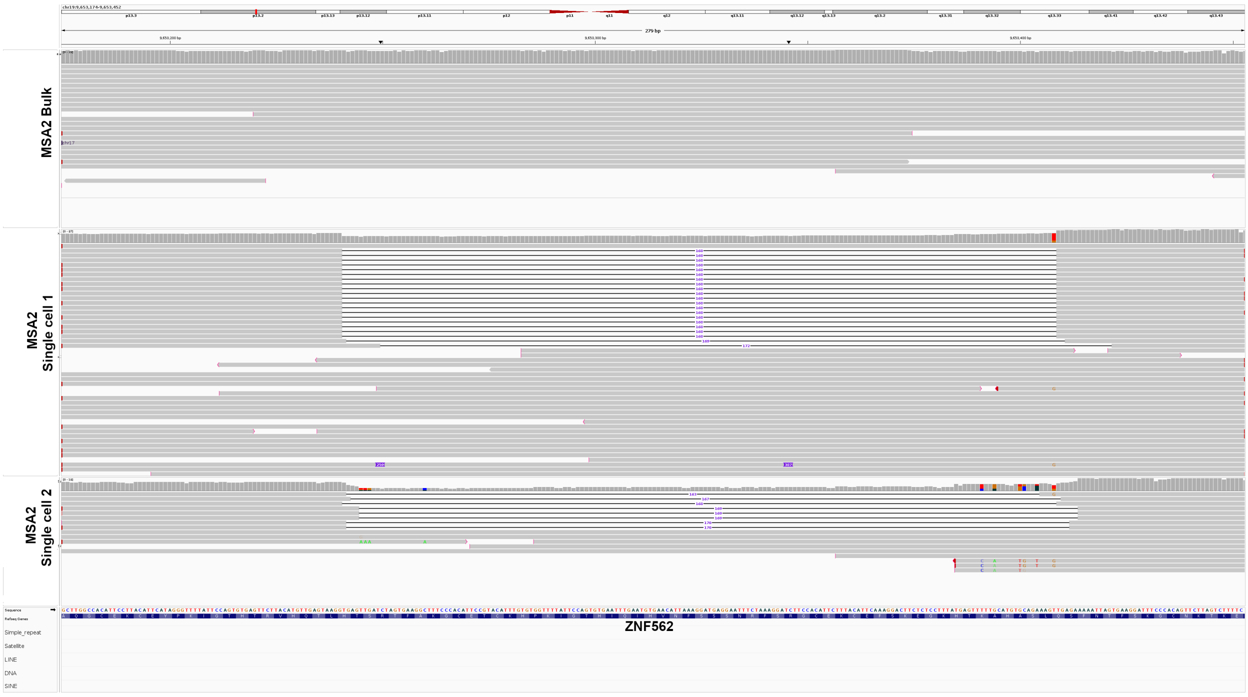

Supplementary Figure 21: Example of a single cell-specific deletion detected in MSA2 brain. MAPQ=58.7024/60 [PromethION/MinION]. The first single cell sample represent aggregated reads from 6 single cells sequenced with T7 and the second 6 single cells sequenced with RBP.

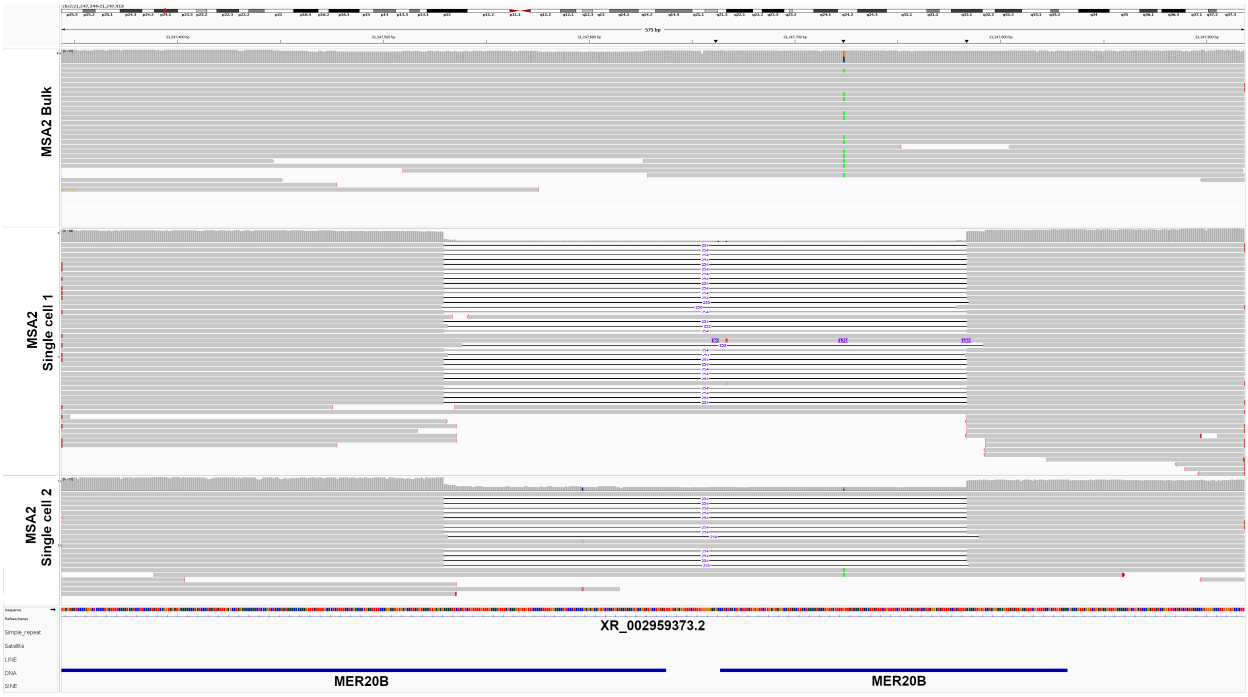

Supplementary Figure 22: Example of a single cell-specific deletion detected in MSA2 brain. For both single-cell samples MAPQ=60. The first single cell sample represent aggregated reads from 6 single cells sequenced with T7 and the second 6 single cells sequenced with RBP.

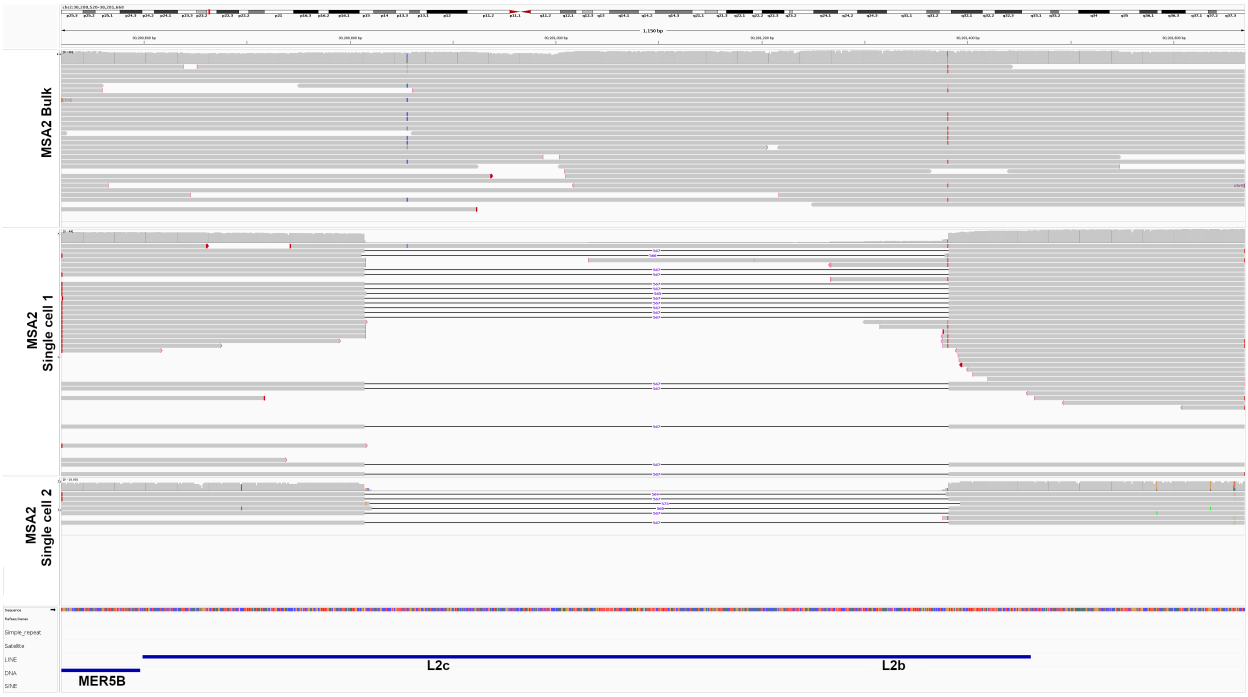

Supplementary Figure 23: Example of a single cell-specific deletion detected in MSA2 brain. For both single-cell samples MAPQ=60. The first single cell sample represent aggregated reads from 6 single cells sequenced with T7 and the second 6 single cells sequenced with RBP

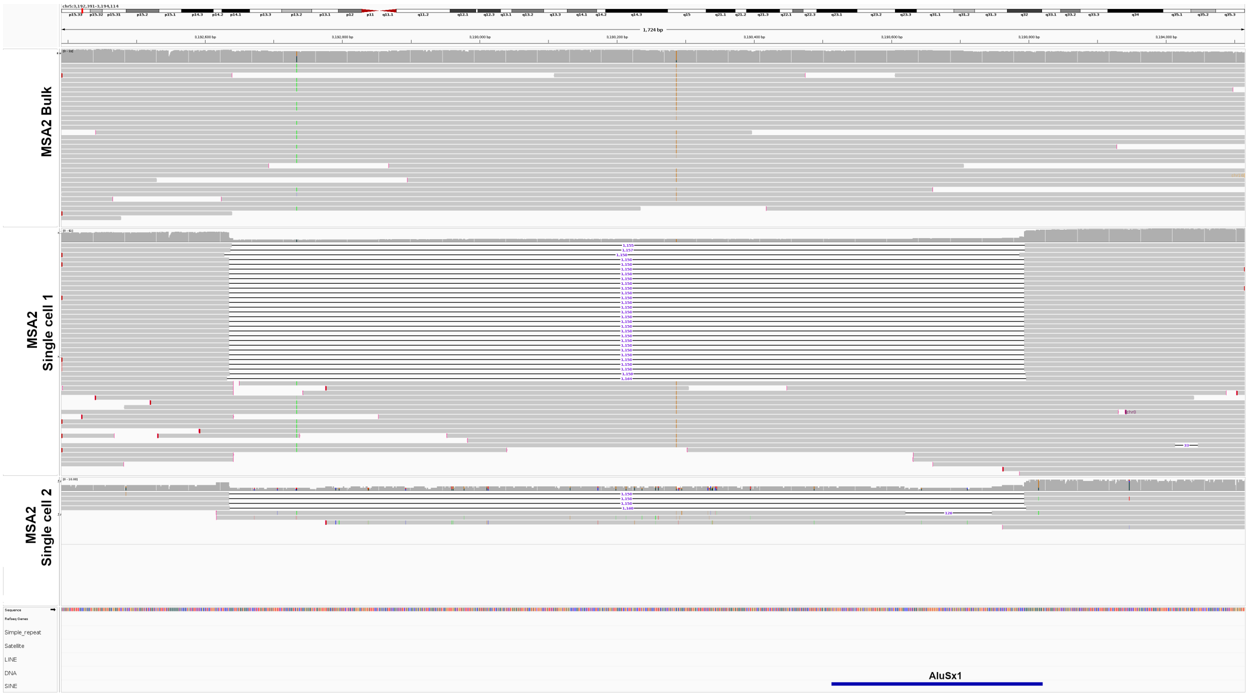

Supplementary Figure 24: Example of a single cell-specific deletion detected in MSA2 brain. For both single-cell samples MAPQ=60. The first single cell sample represent aggregated reads from 6 single cells sequenced with T7 and the second 6 single cells sequenced with RBP.

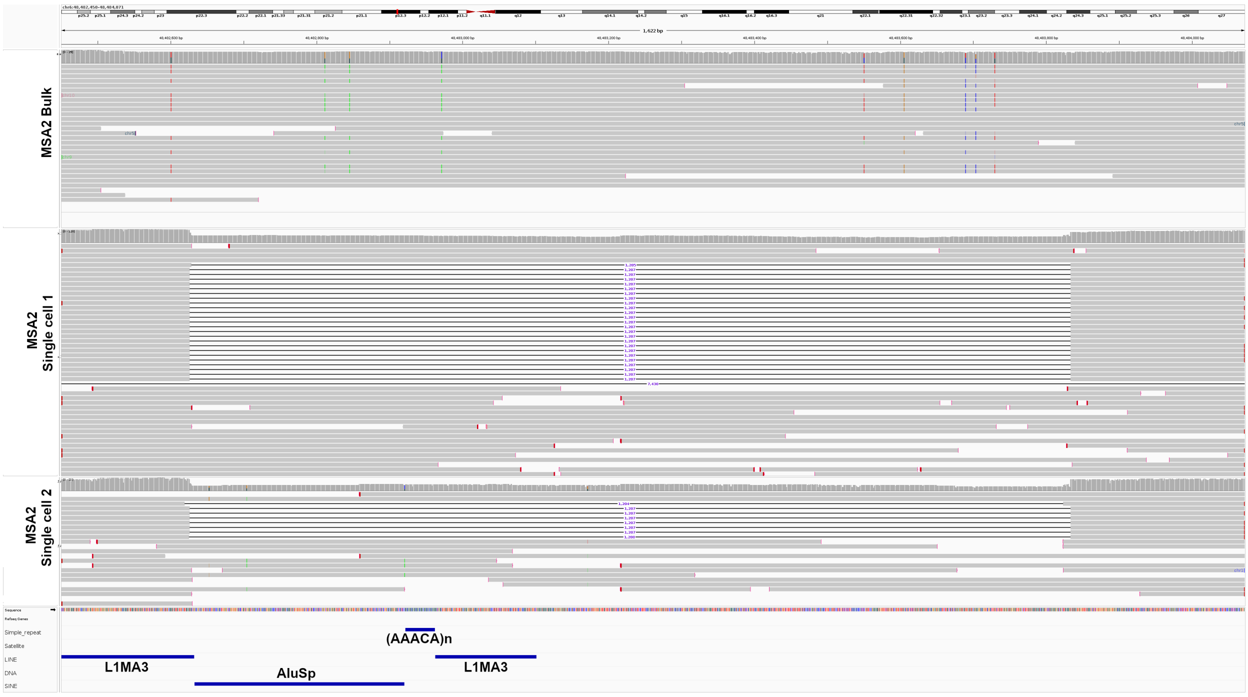

Supplementary Figure 25: Example of a single cell-specific deletion detected in MSA2 brain. MAPQ= 59.4346/60 [PromethION/MinION]. The first single cell sample represent aggregated reads from 6 single cells sequenced with T7 and the second 6 single cells sequenced with RBP.

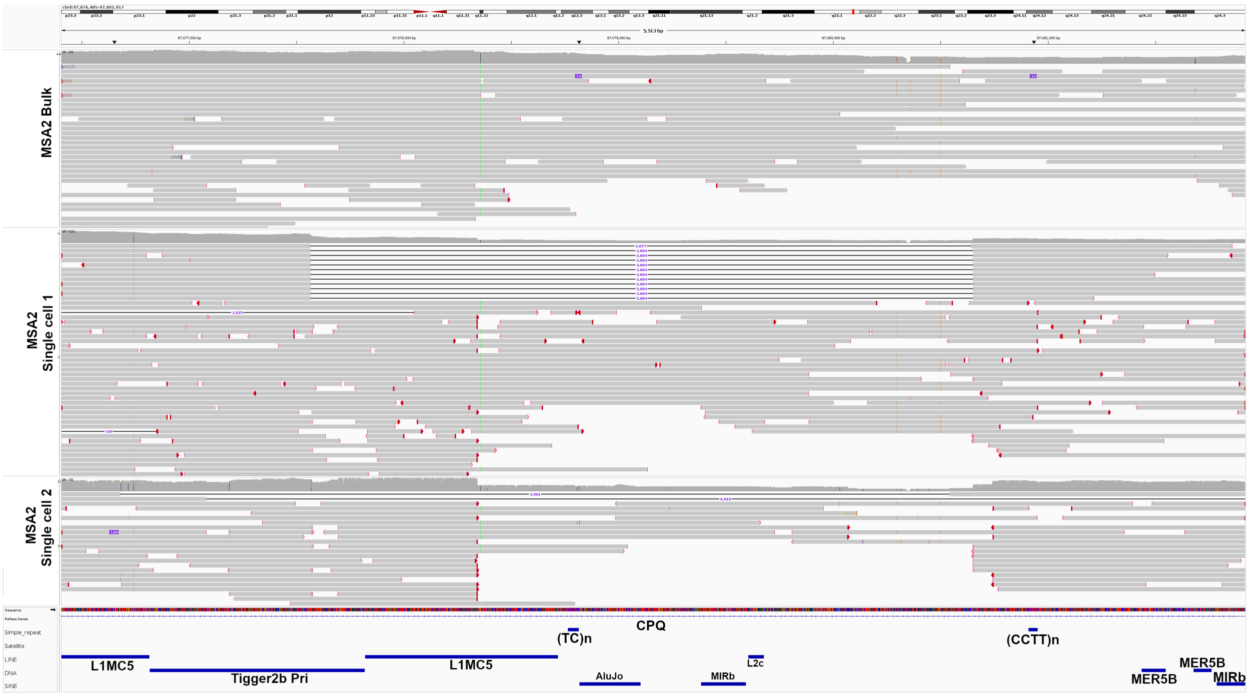

Supplementary Figure 26: Example of a single cell-specific deletion detected in MSA2 brain. MAPQ=59.5468/60 [PromethION/MinION]. The first single cell sample represent aggregated reads from 6 single cells sequenced with T7 and the second 6 single cells sequenced with RBP.

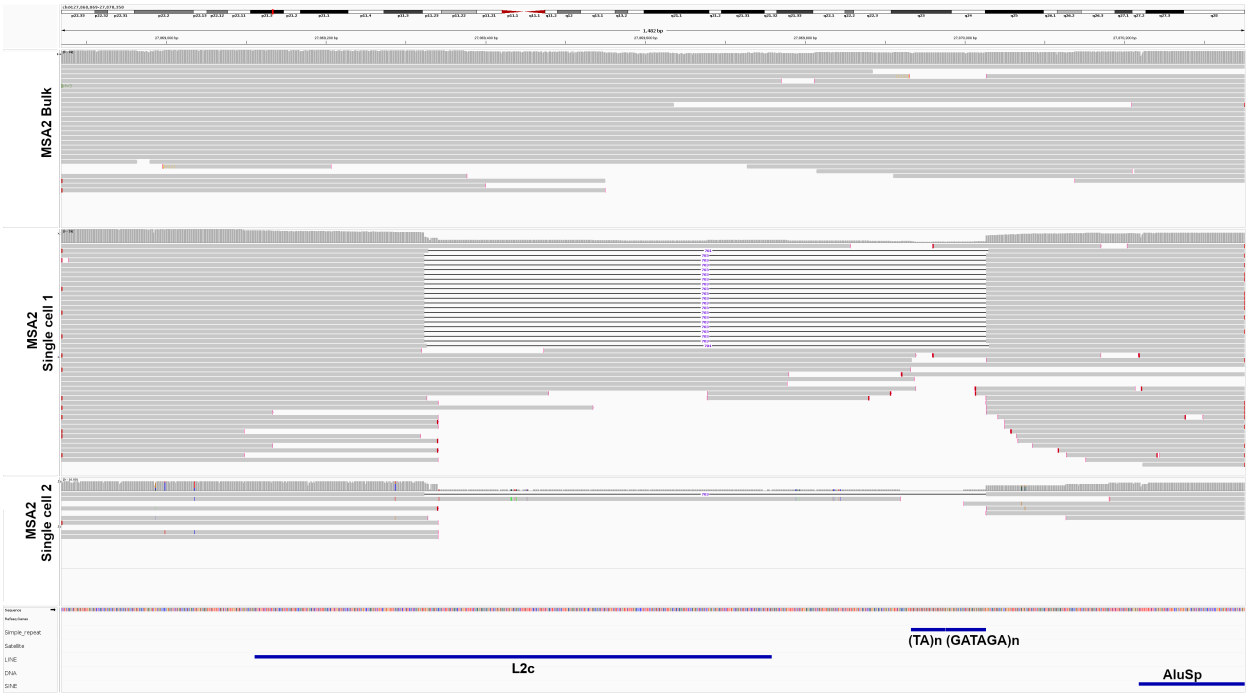

Supplementary Figure 27: Example of a single cell-specific deletion detected in MSA2 brain. For both single-cell samples MAPQ=60. The first single cell sample represent aggregated reads from 6 single cells sequenced with T7 and the second 6 single cells sequenced with RBP.

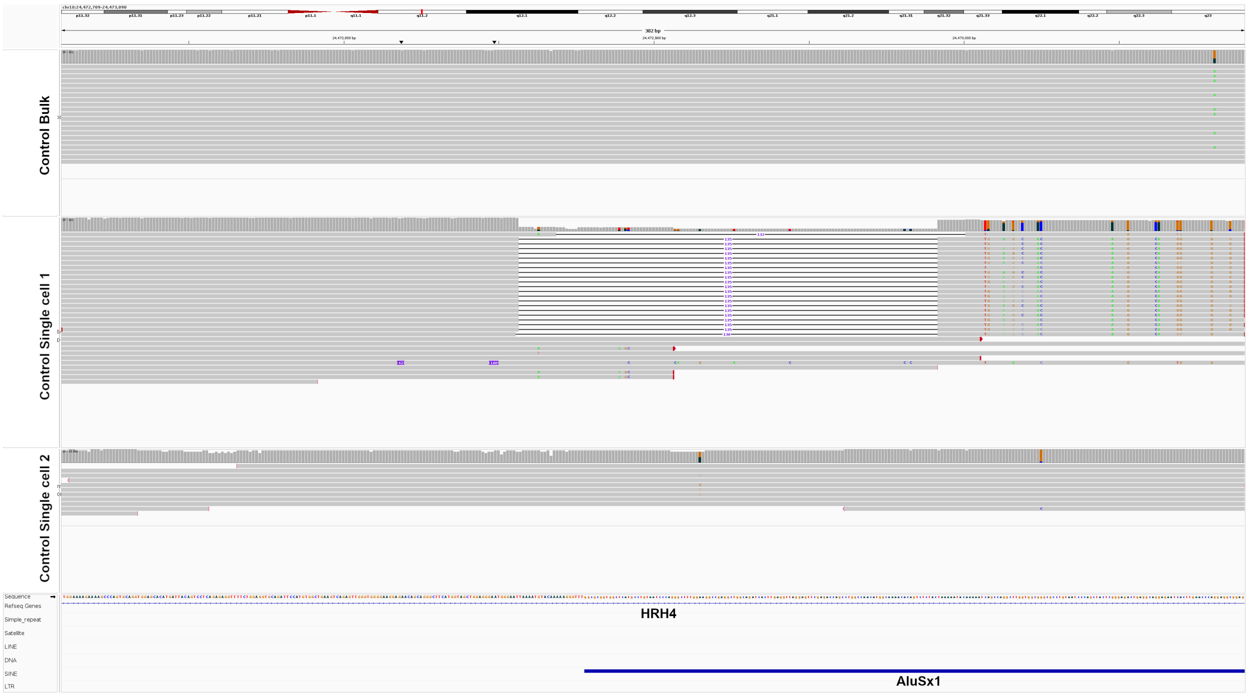

Supplementary Figure 28: Example of a single cell-specific deletion detected in control brain. For both single-cell samples MAPQ=60. The first single cell sample represent aggregated reads from 6 single cells sequenced with T7 and the second 6 single cells sequenced with RBP.

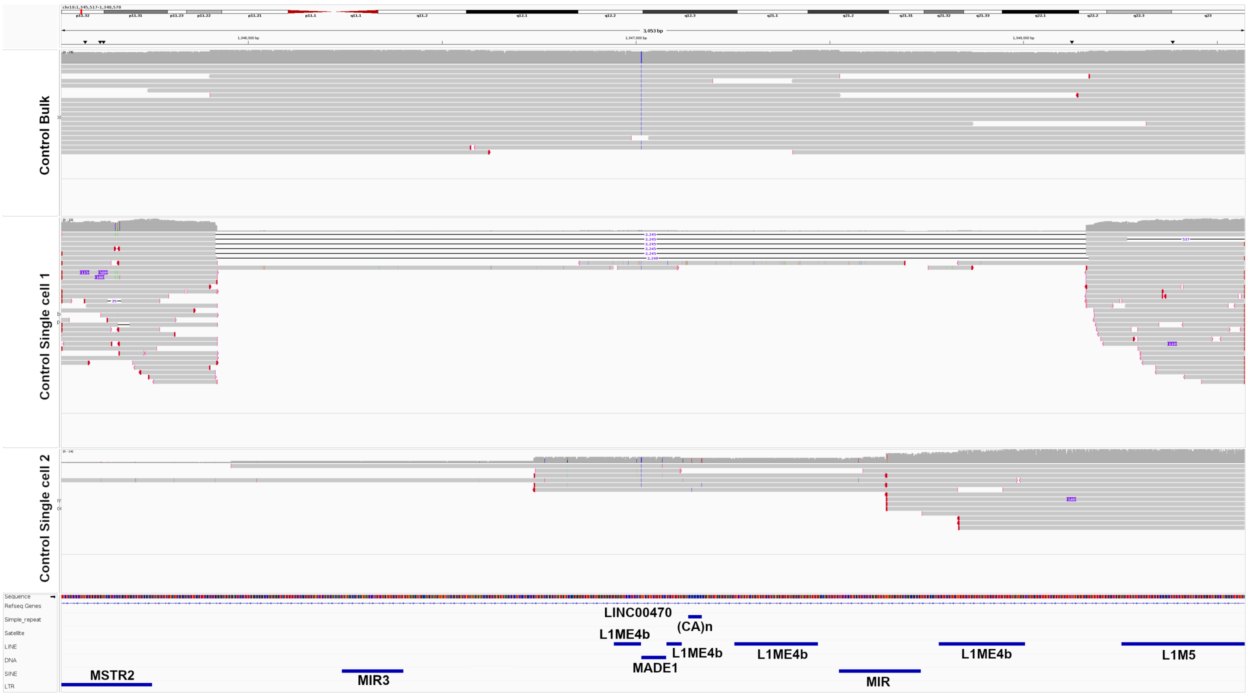

Supplementary Figure 29: Example of a single cell-specific deletion detected in control brain. MAPQ= 54.8182/ 60 [PromethION/MinION]. The first single cell sample represent aggregated reads from 6 single cells sequenced with T7 and the second 6 single cells sequenced with RBP.

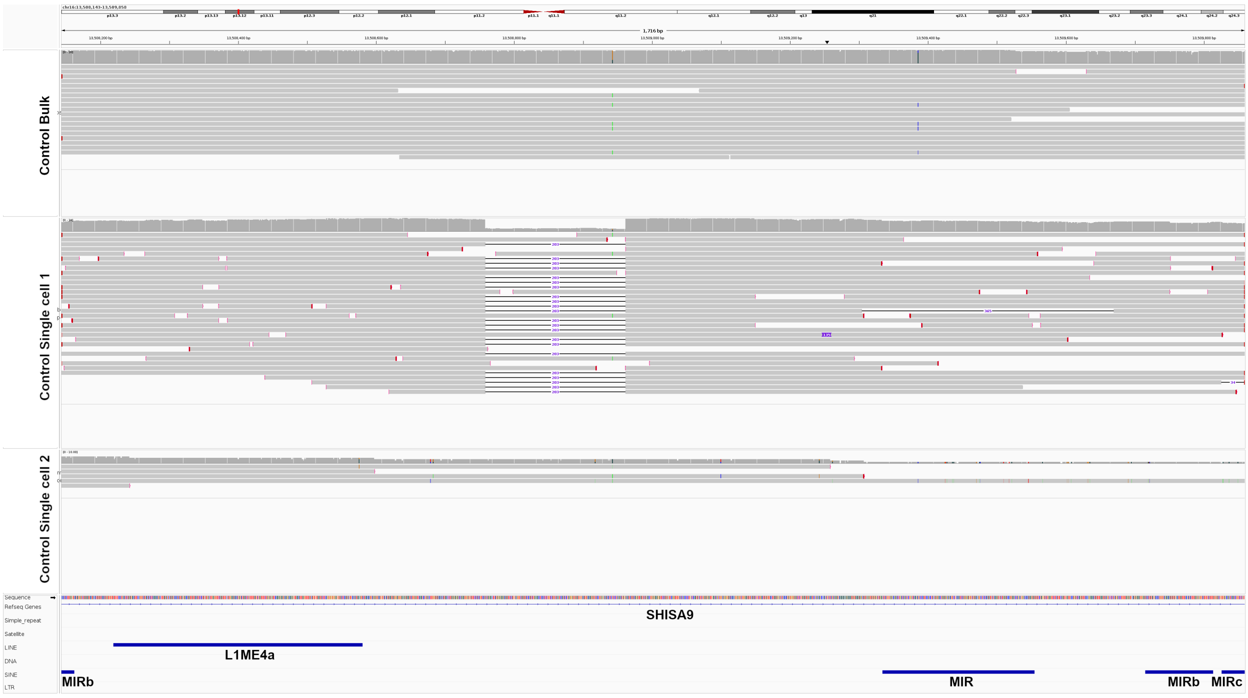

Supplementary Figure 30: Example of a single cell-specific deletion detected in control brain. For both single-cell samples MAPQ=60. The first single cell sample represent aggregated reads from 6 single cells sequenced with T7 and the second 6 single cells sequenced with RBP.

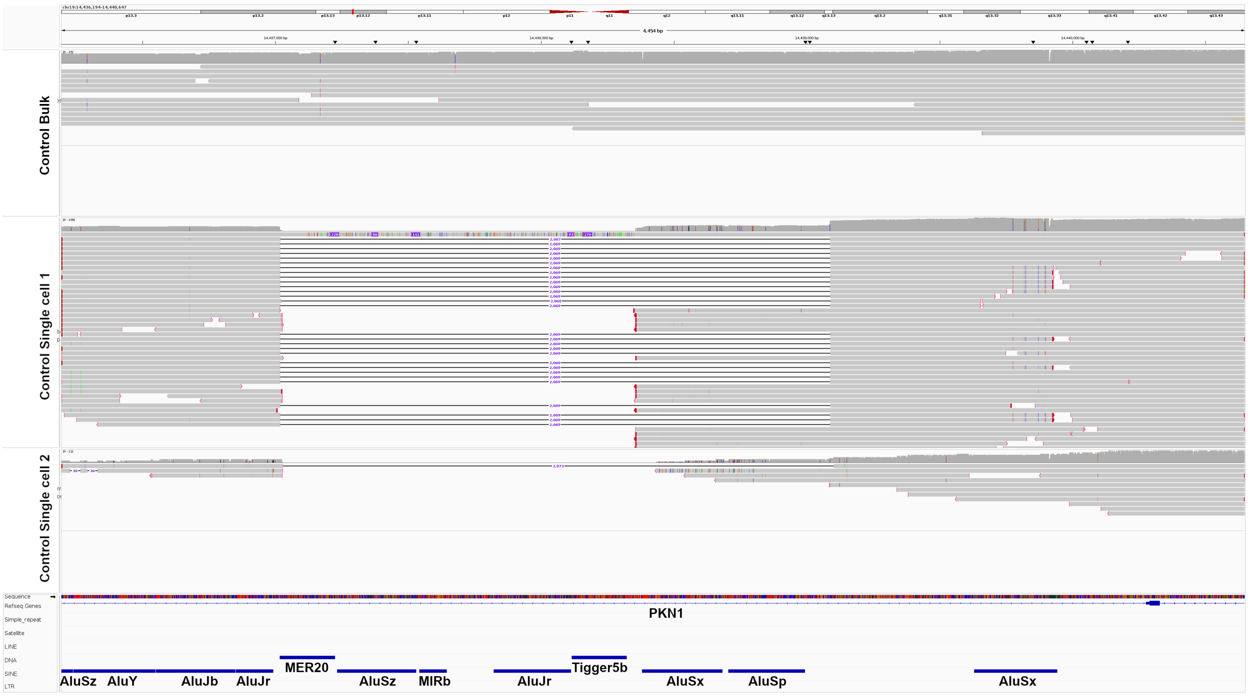

Supplementary Figure 31: Example of a single cell-specific deletion detected in control brain. MAPQ=58.7453/59.2857 [PromethION/MinION]. The first single cell sample represent aggregated reads from 6 single cells sequenced with T7 and the second 6 single cells sequenced with RBP.

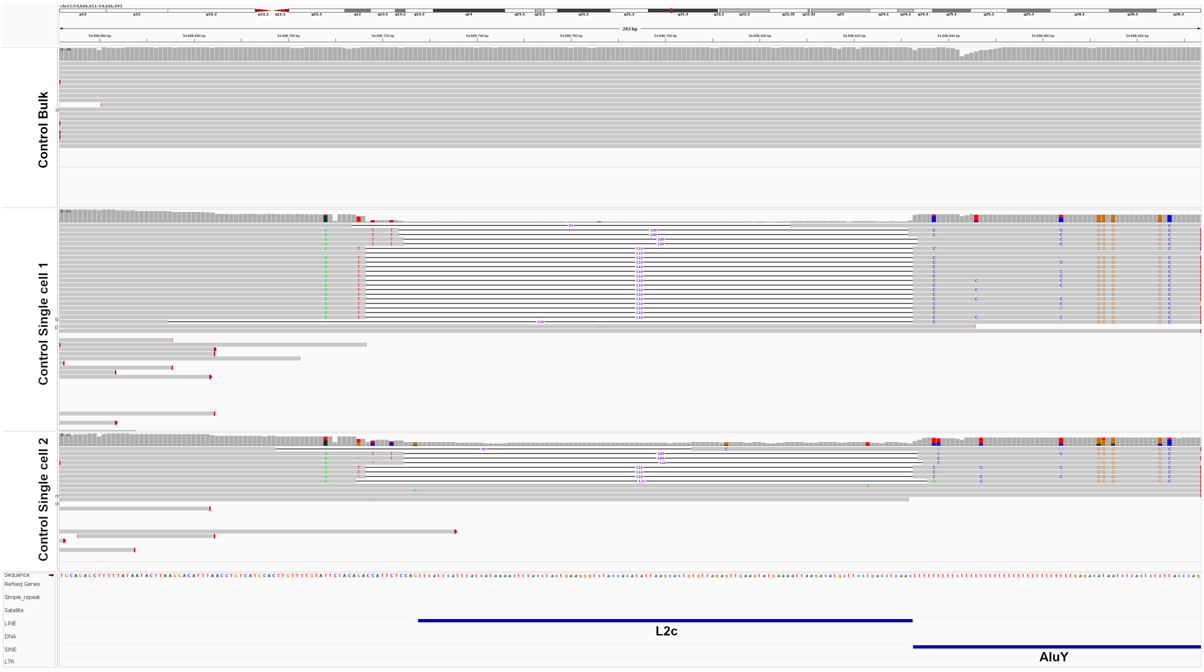

Supplementary Figure 32: Example of a single cell-specific deletion detected in control brain. For both single-cell samples MAPQ=60. The first single cell sample represent aggregated reads from 6 single cells sequenced with T7 and the second 6 single cells sequenced with RBP.

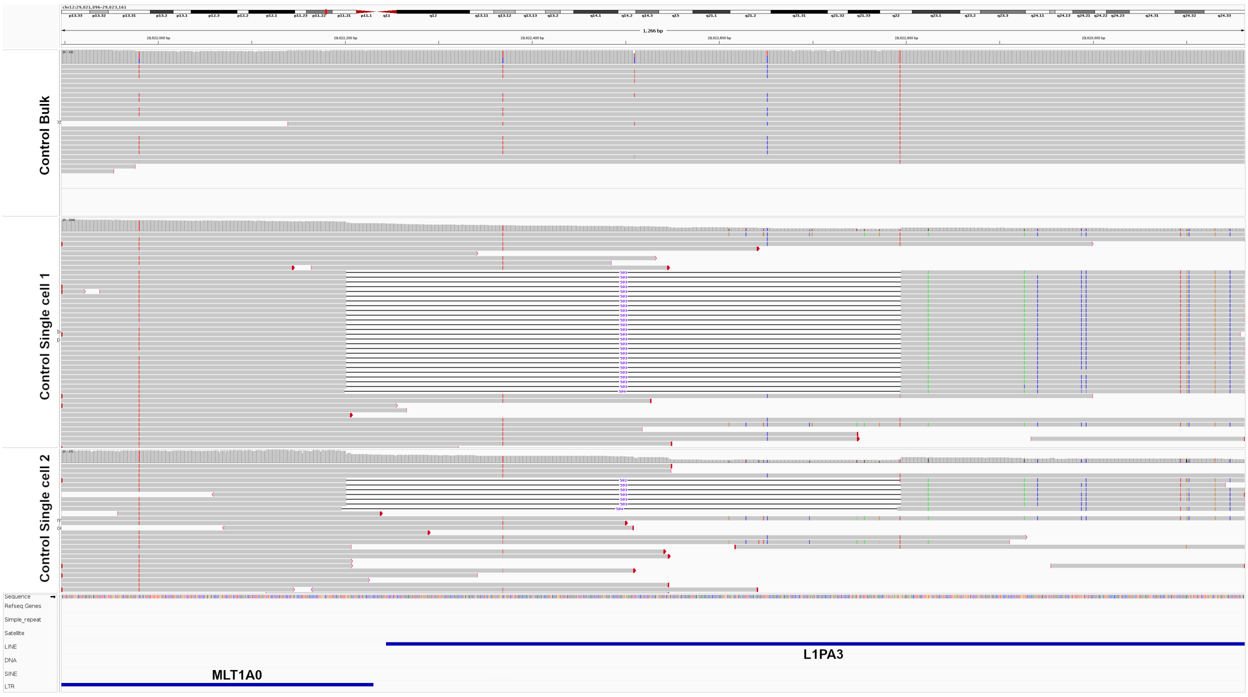

Supplementary Figure 33: Example of a single cell-specific deletion detected in control brain. MAPQ= 56.0086/ 59.1429 [PromethION/MinION]. The first single cell sample represent aggregated reads from 6 single cells sequenced with T7 and the second 6 single cells sequenced with RBP.

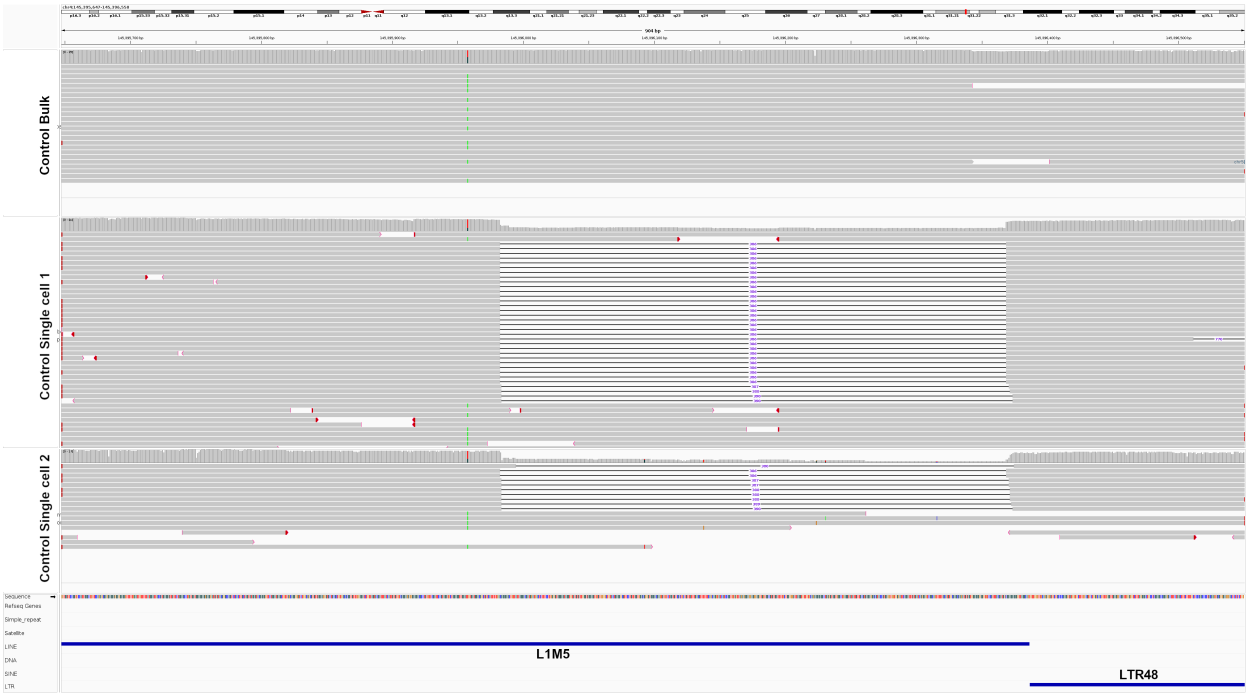

Supplementary Figure 34: Example of a single cell-specific deletion detected in control brain. MAPQ= 59.8235/ 60 [PromethION/MinION]. The first single cell sample represent aggregated reads from 6 single cells sequenced with T7 and the second 6 single cells sequenced with RBP.

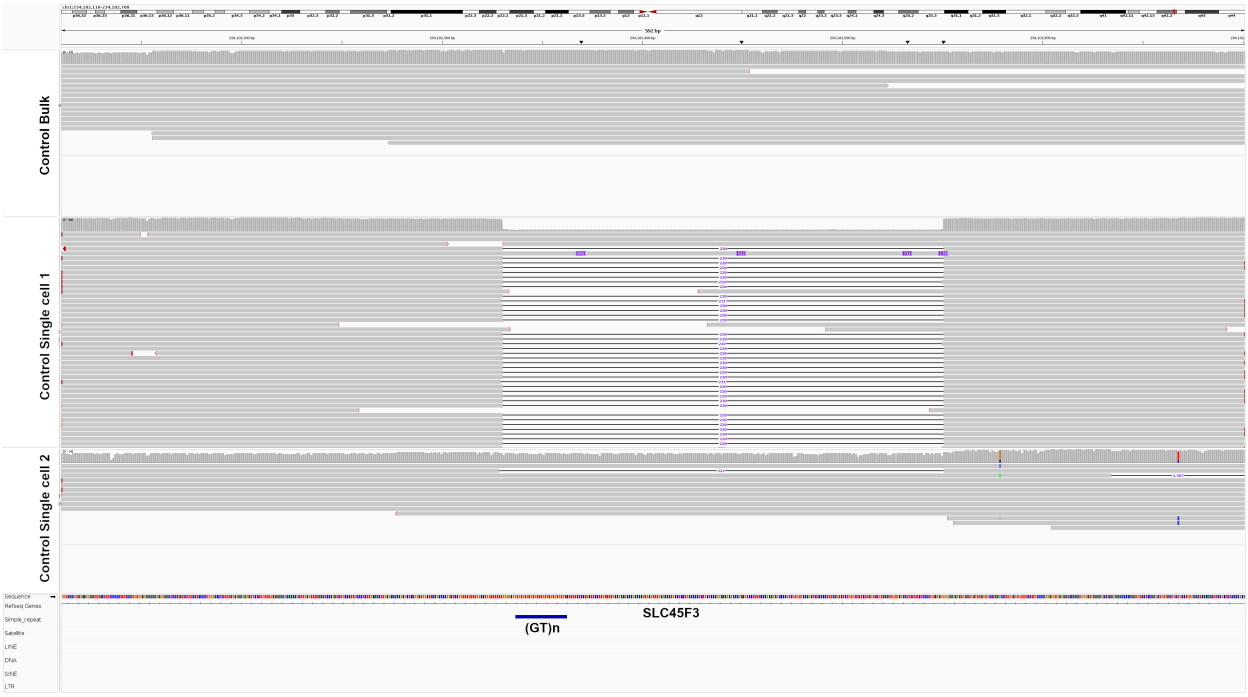

Supplementary Figure 35: Example of a single cell-specific deletion detected in control brain. For both single-cell samples MAPQ=60. The first single cell sample represent aggregated reads from 6 single cells sequenced with T7 and the second 6 single cells sequenced with RBP.

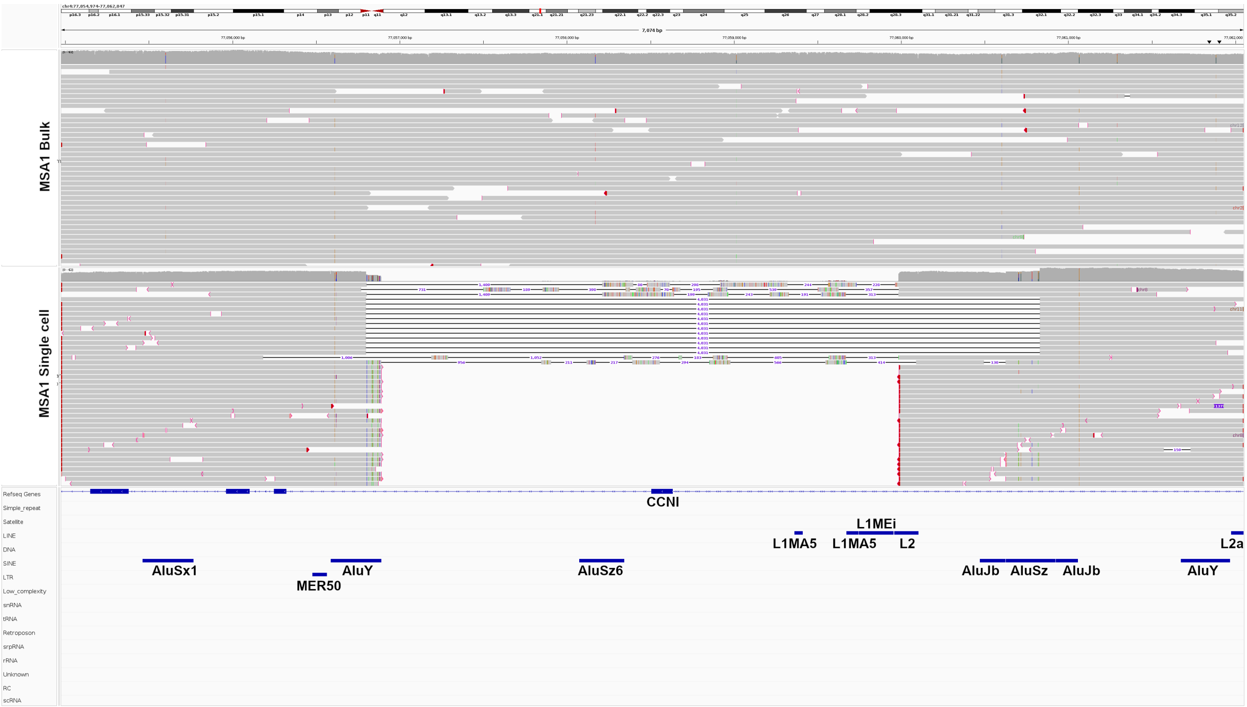

Supplementary Figure 36: Example of recombinational deletion between two SINE/Alu elements (AluY - AluJb) – MSA1. MAPQ= 58.3153. The single cell sample represent aggregated reads from 6 single cells sequenced with RBP.

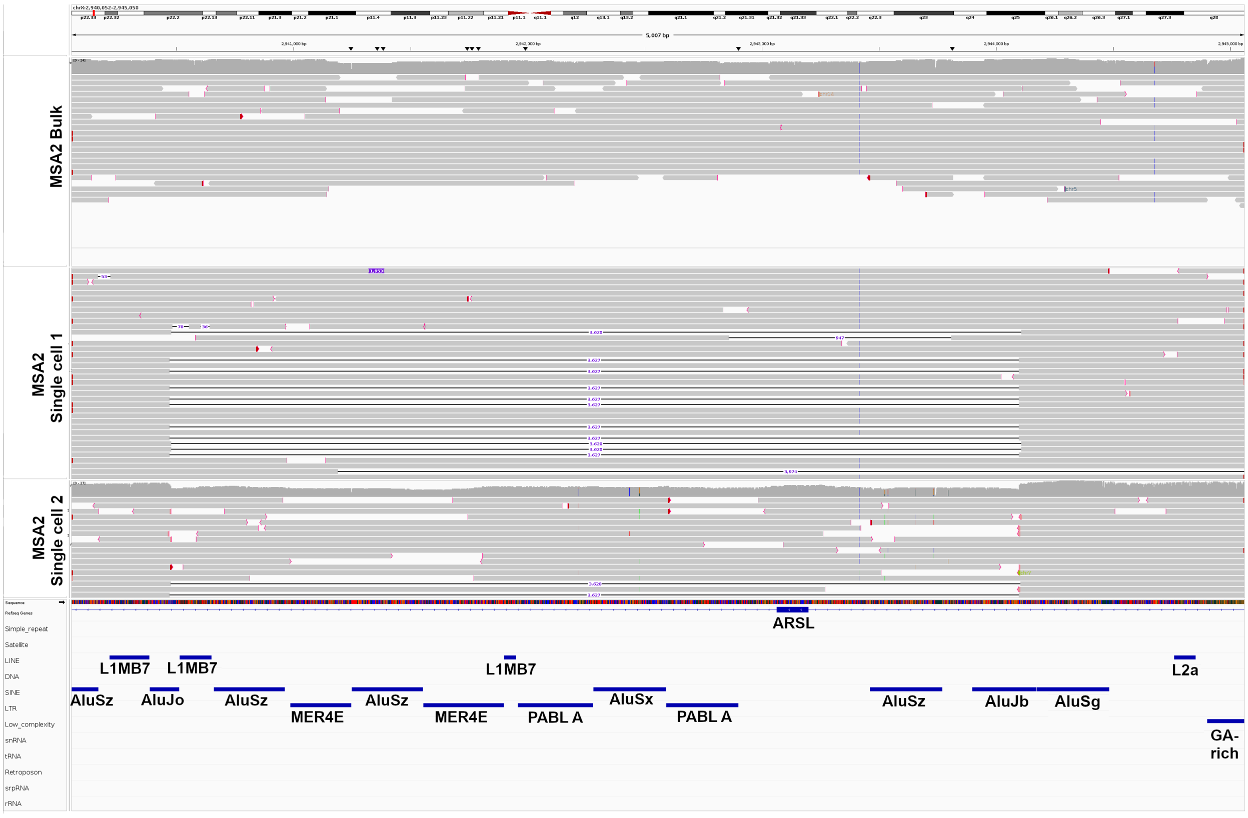

Supplementary Figure 37: Example of recombinational deletion between two SINE/Alu (AluJo – AluJb) elements – MSA2. MAPQ= 59.5098/ 58.0962 [PromethION/MinION]. The first single cell sample represent aggregated reads from 6 single cells sequenced with T7 and the second 6 single cells sequenced with RBP.

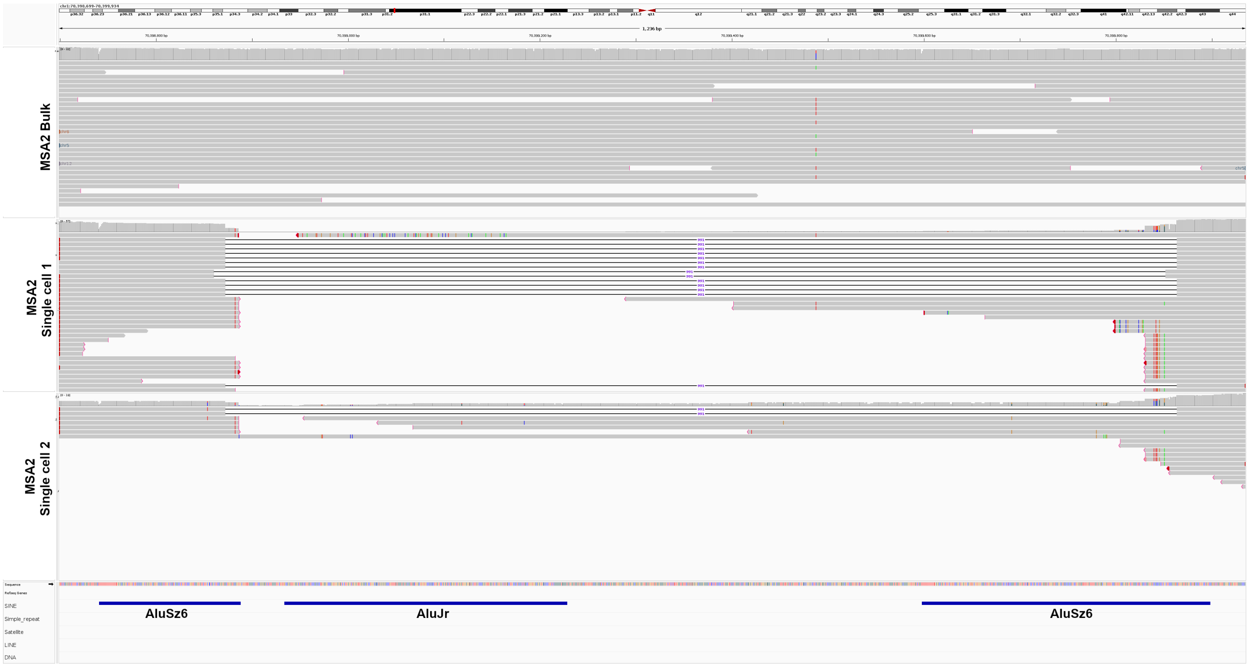

Supplementary Figure 38: Example of recombinational deletion between two SINE/Alu (AluSz6 – AluSz6) elements – MSA2. MAPQ=59.2917/60 [PromethION/MinION]. The first single cell sample represent aggregated reads from 6 single cells sequenced with T7 and the second 6 single cells sequenced with RBP.

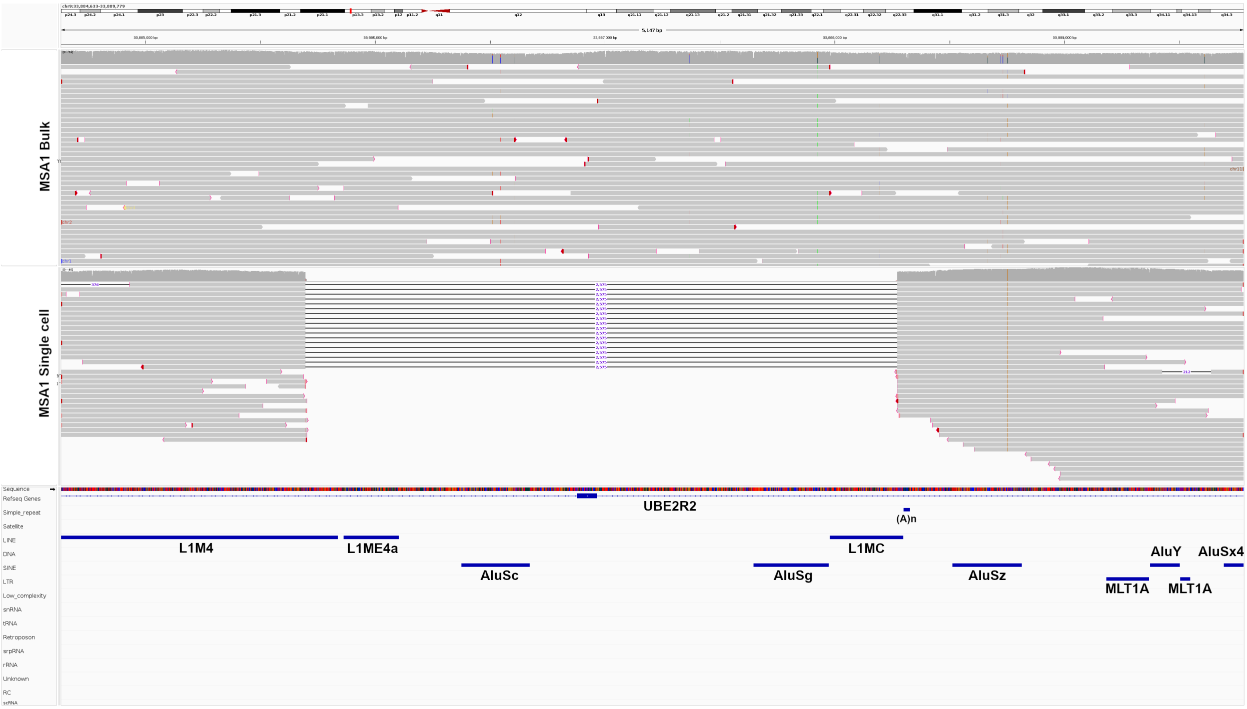

Supplementary Figure 39: Example of recombinational deletion between two LINE/L1 (L1M4 – L1MC) elements – MSA1. MAPQ= 58.9118. The single cell sample represent aggregated reads from 6 single cells sequenced with RBP.

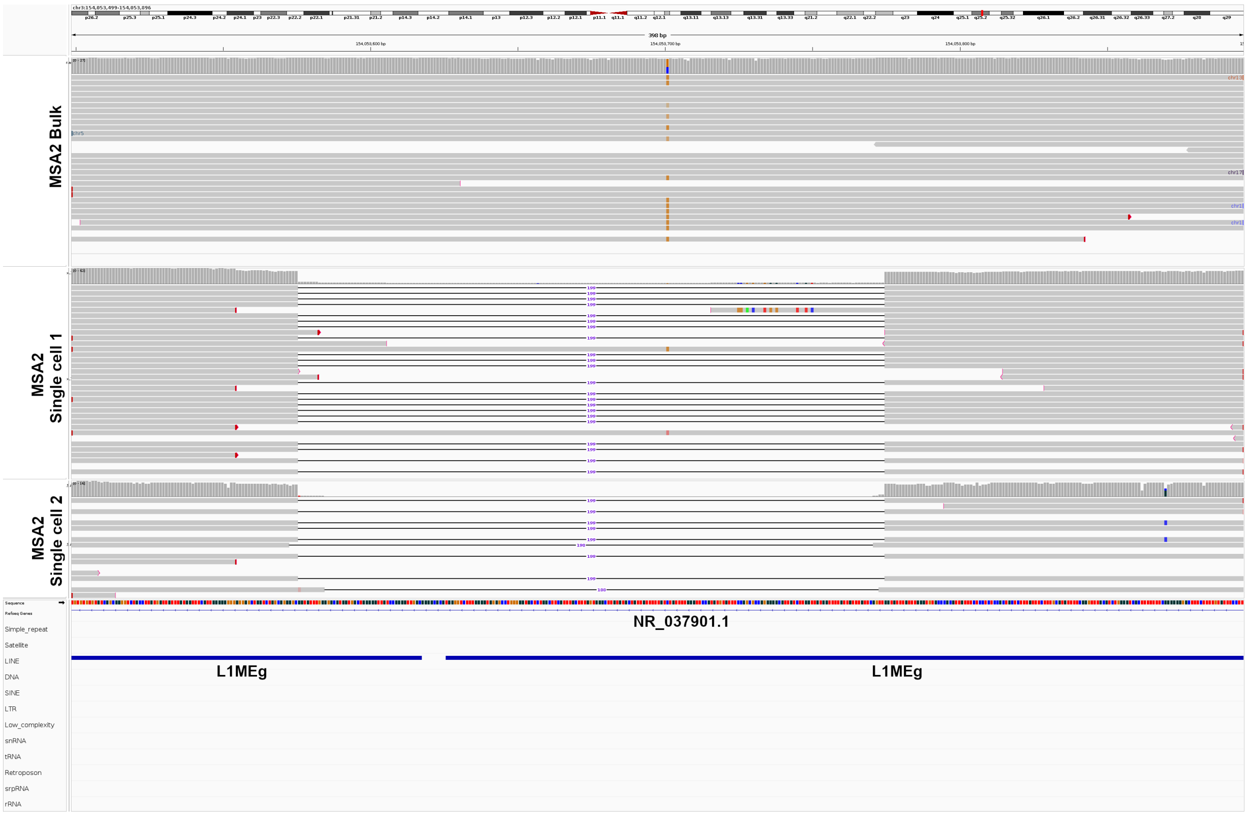

Supplementary Figure 40: Example of recombinational deletion between two LINE/L1 (L1MEg – L1MEg) elements – MSA2. For both single-cell samples MAPQ=60. The first single cell sample represent aggregated reads from 6 single cells sequenced with T7 and the second 6 single cells sequenced with RBP.

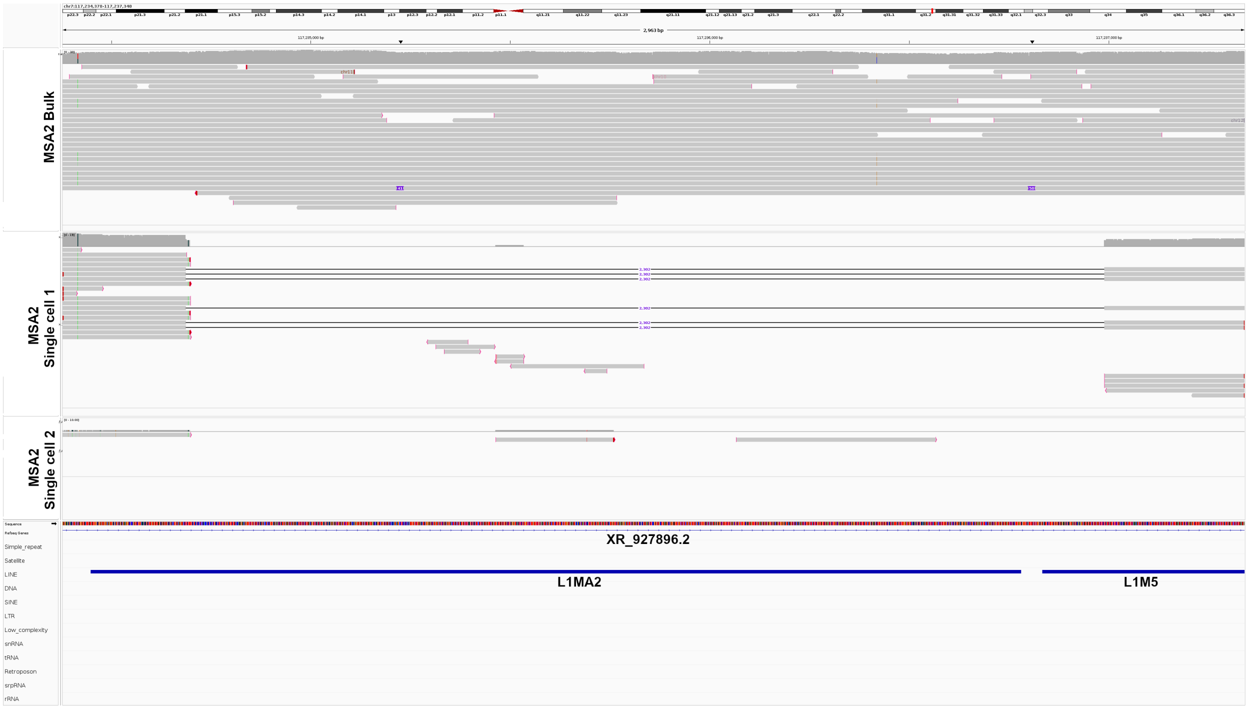

Supplementary Figure 41: Example of recombinational deletion between two LINE/L1 (L1MA2 – L1M5) elements – MSA2. For both single-cell samples MAPQ=60. The first single cell sample represent aggregated reads from 6 single cells sequenced with T7 and the second 6 single cells sequenced with RBP.

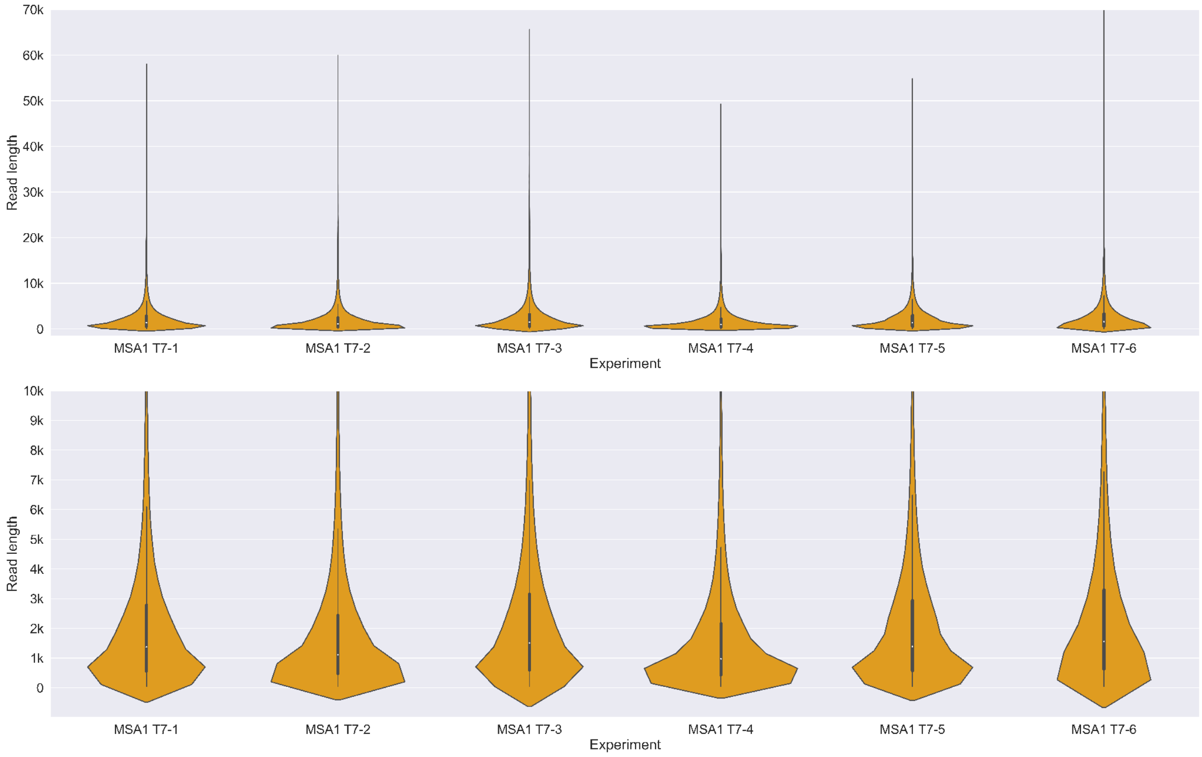

Supplementary Figure 42: Top panel, MSA1 T7 read length distribution, limited to 70,000bp; the MSA1 T7-6 sample peaks at ~90,000bp. Due to low yield, we omit these samples from the analysis; bottom panel, MSA1 T7 read length distribution, limited to 10,000bp.

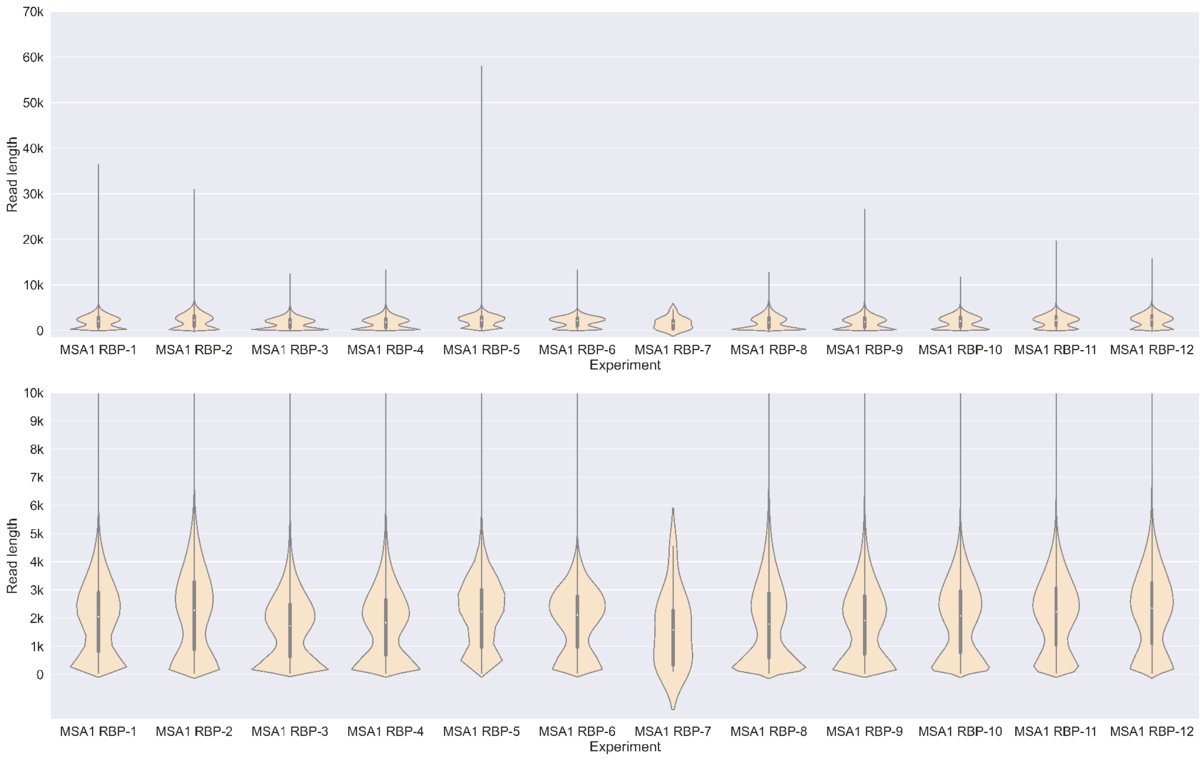

Supplementary Figure 43: Top panel, MSA1 RBP read length distribution, limited to 70,000bp. We sequenced 6 single cells twice with MinION device.; bottom panel, MSA1 RBP read length distribution, limited to 10,000bp.

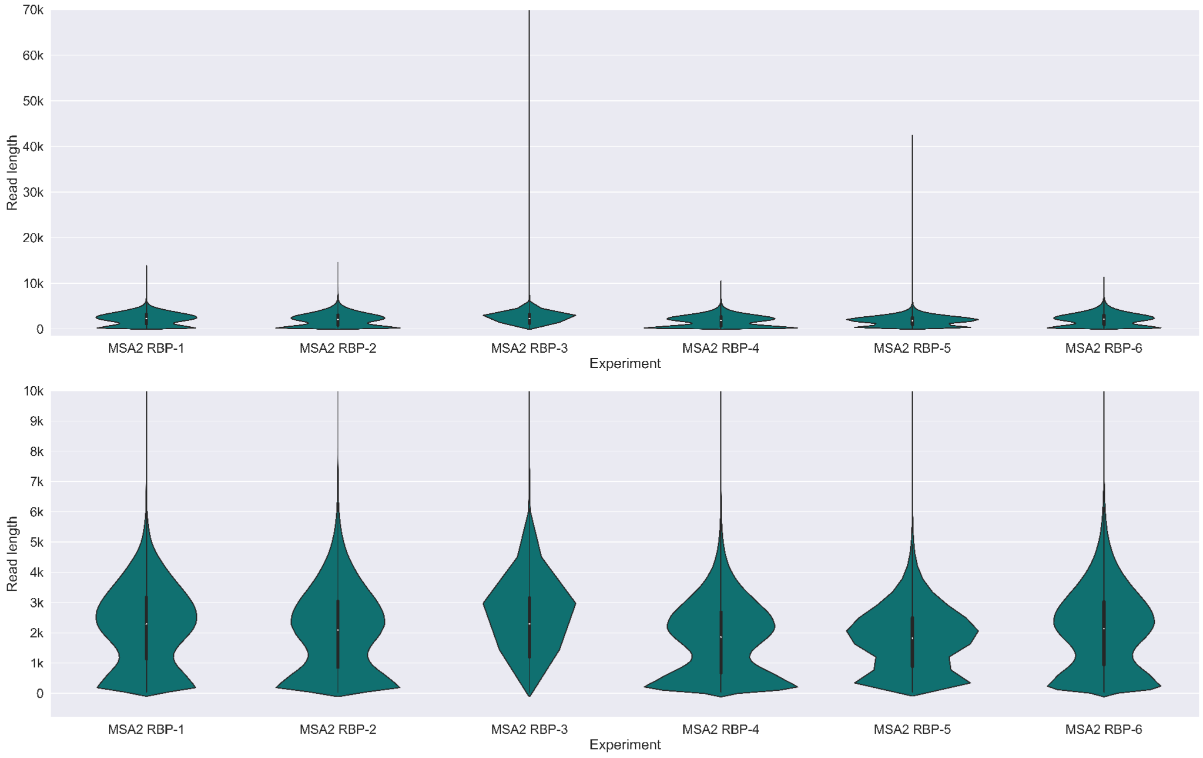

Supplementary Figure 44: Top panel, MSA2 RBP read length distribution, limited to 70,000bp; the MSA2 RBP-3 sample longest read is at ~1,500,000bp; Bottom panel, MSA2 RBP read length distribution, limited to 10,000bp.

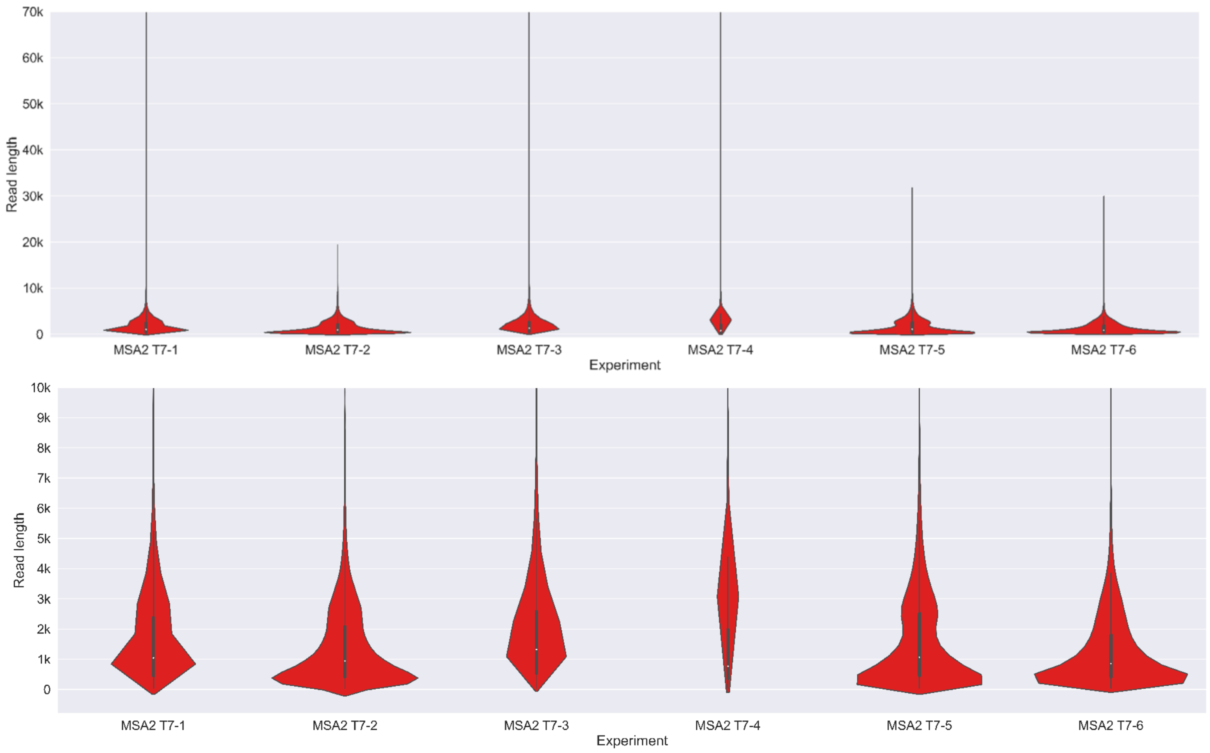

Supplementary Figure 45: Top panel, MSA2 T7 read length distribution, limited to 70,000bp; the MSA2 T7-1 sample peaks at ~100,000bp, MSA2 T7-3 at ~120,000bp and MSA2 T7-4 at ~120,000bp. Bottom panel, MSA2 T7 read length distribution, limited to 10,000bp.

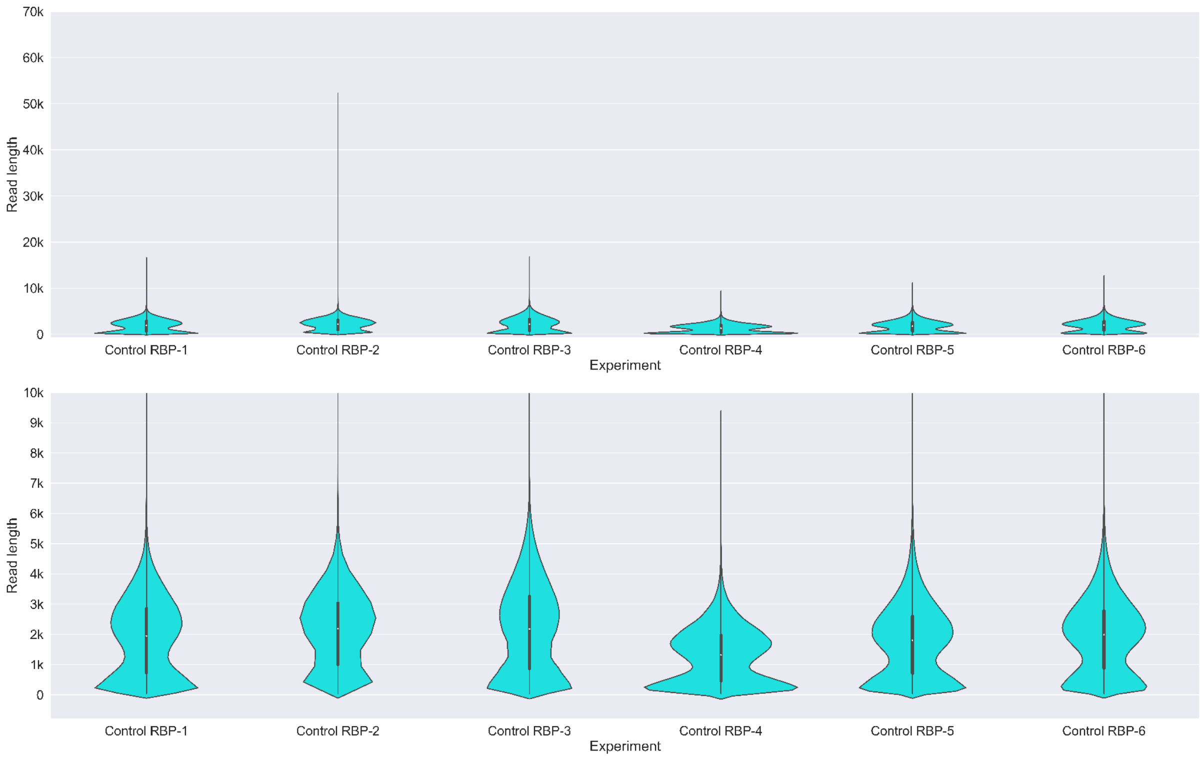

Supplementary Figure 46: Top panel, Control RBP read length distribution, limited to 70,000bp; Bottom panel, Control RBP read length distribution, limited to 10,000bp.

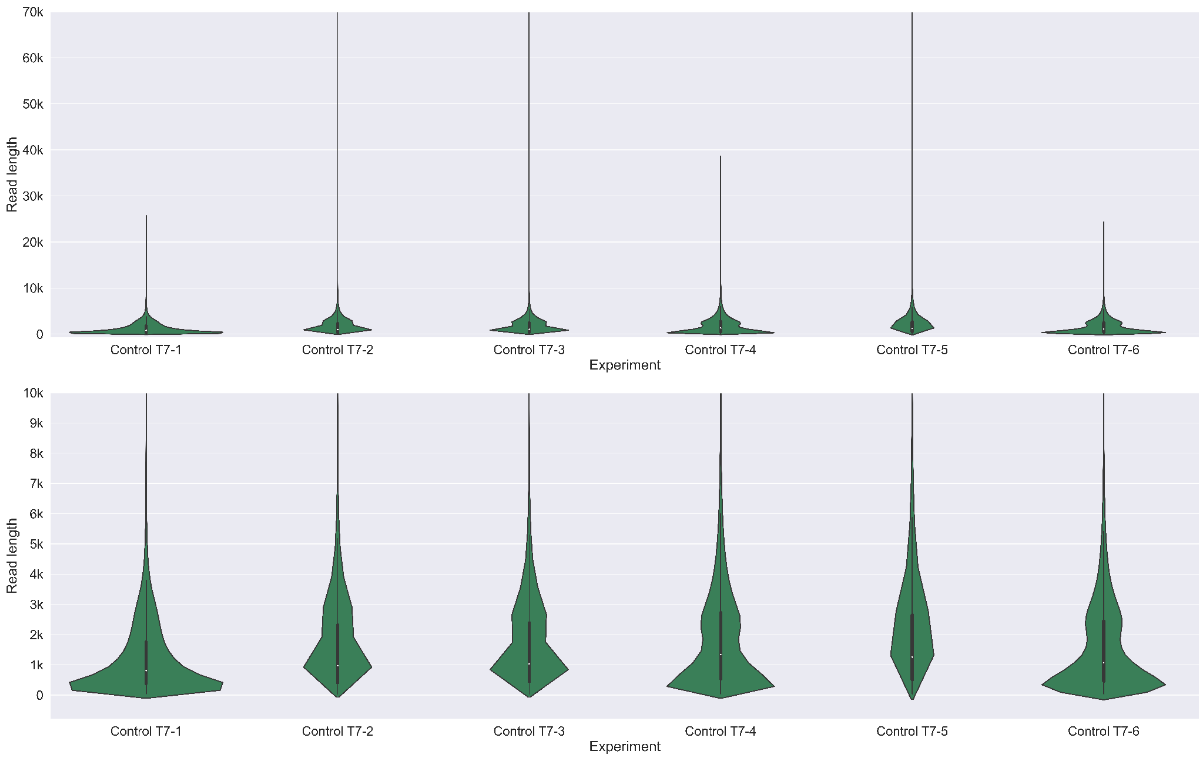

Supplementary Figure 47: Top panel, Control T7 read length distribution, limited to 70,000bp; the Control T7-2 sample peaks at ~100,000bp, Control T7-3 at ~90,000bp and Control T7-5 at ~140,000bp; Bottom panel, Control T7 read length distribution, limited to 10,000bp.

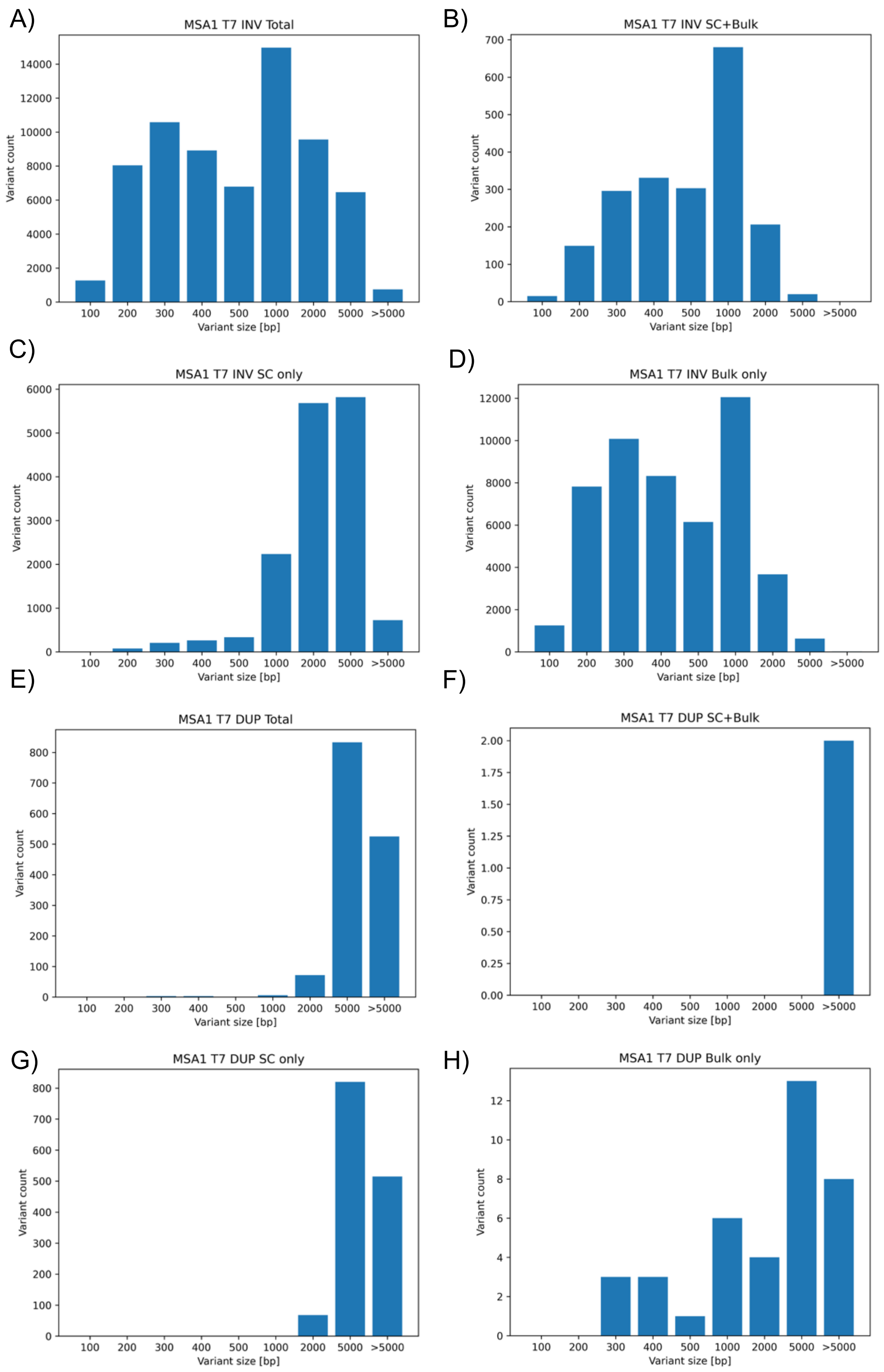

Supplementary Figure 48: Inversion and duplication sizes - unfiltered - MSA1 brain T7 library preparation A) Total number of reported inversions B) Inversions found in single cells and corresponding bulk C) Inversions found in single cells only D) Inversions found in bulk only E) Total number of reported duplications F) Duplications found in single cells and corresponding bulk G) Duplications found in single cells only H) Duplications found in bulk only.

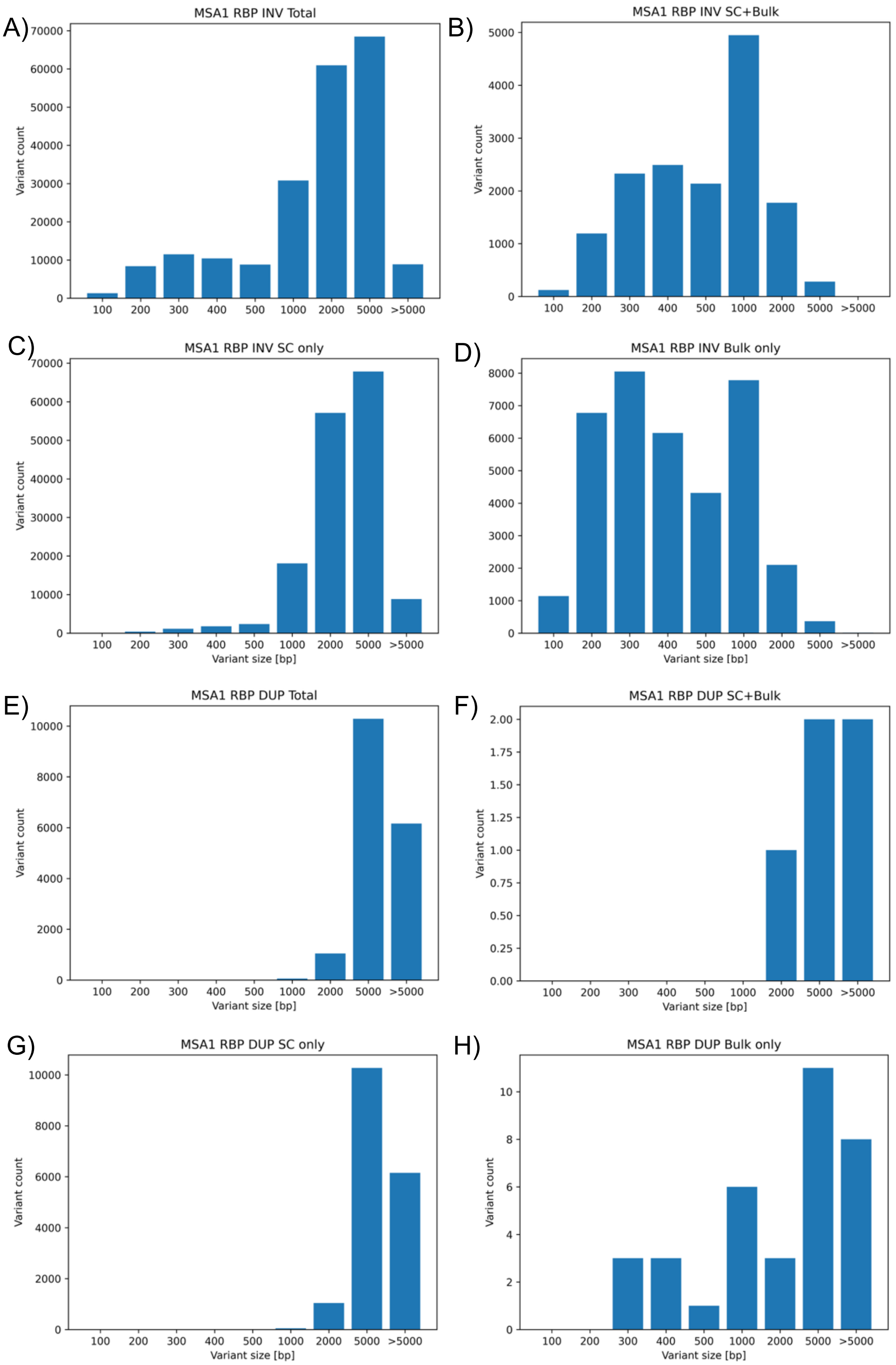

Supplementary Figure 49: Inversion and duplication sizes - unfiltered - MSA1 brain RBP library preparation A) Total number of reported inversions B) Inversions found in single cells and corresponding bulk C) Inversions found in single cells only D) Inversions found in bulk only E) Total number of reported duplications F) Duplications found in single cells and corresponding bulk G) Duplications found in single cells only H) Duplications found in bulk only.

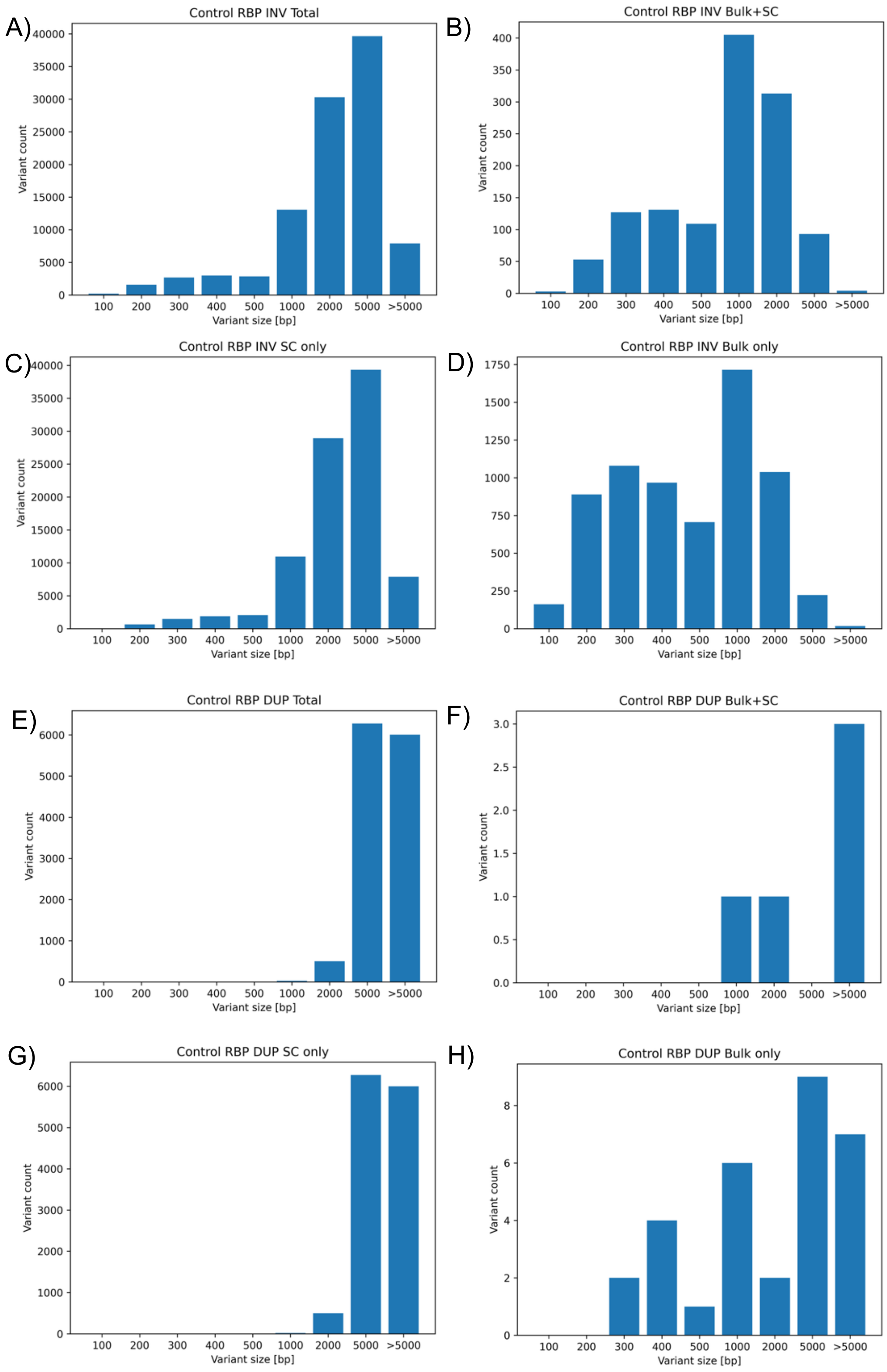

Supplementary Figure 50: Inversion and duplication sizes - unfiltered - Control brain RBP library preparation A) Total number of reported inversions B) Inversions found in single cells and corresponding bulk C) Inversions found in single cells only D) Inversions found in bulk only E) Total number of reported duplications F) Duplications found in single cells and corresponding bulk G) Duplications found in single cells only H) Duplications found in bulk only.

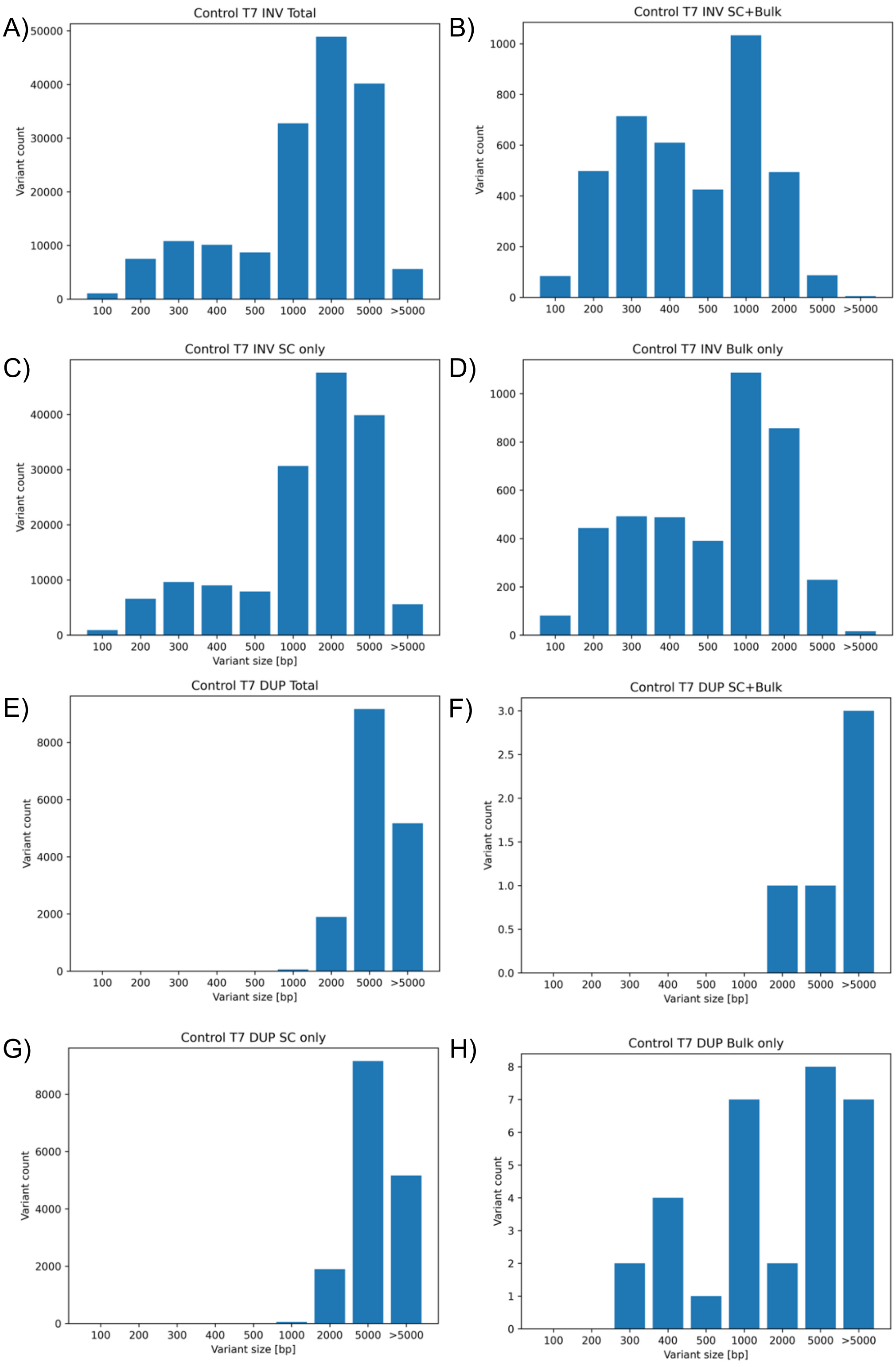

Supplementary Figure 51: Inversion and duplication sizes - unfiltered - Control brain T7 library preparation A) Total number of reported inversions B) Inversions found in single cells and corresponding bulk C) Inversions found in single cells only D) Inversions found in bulk only E) Total number of reported duplications F) Duplications found in single cells and corresponding bulk G) Duplications found in single cells only H) Duplications found in bulk only.

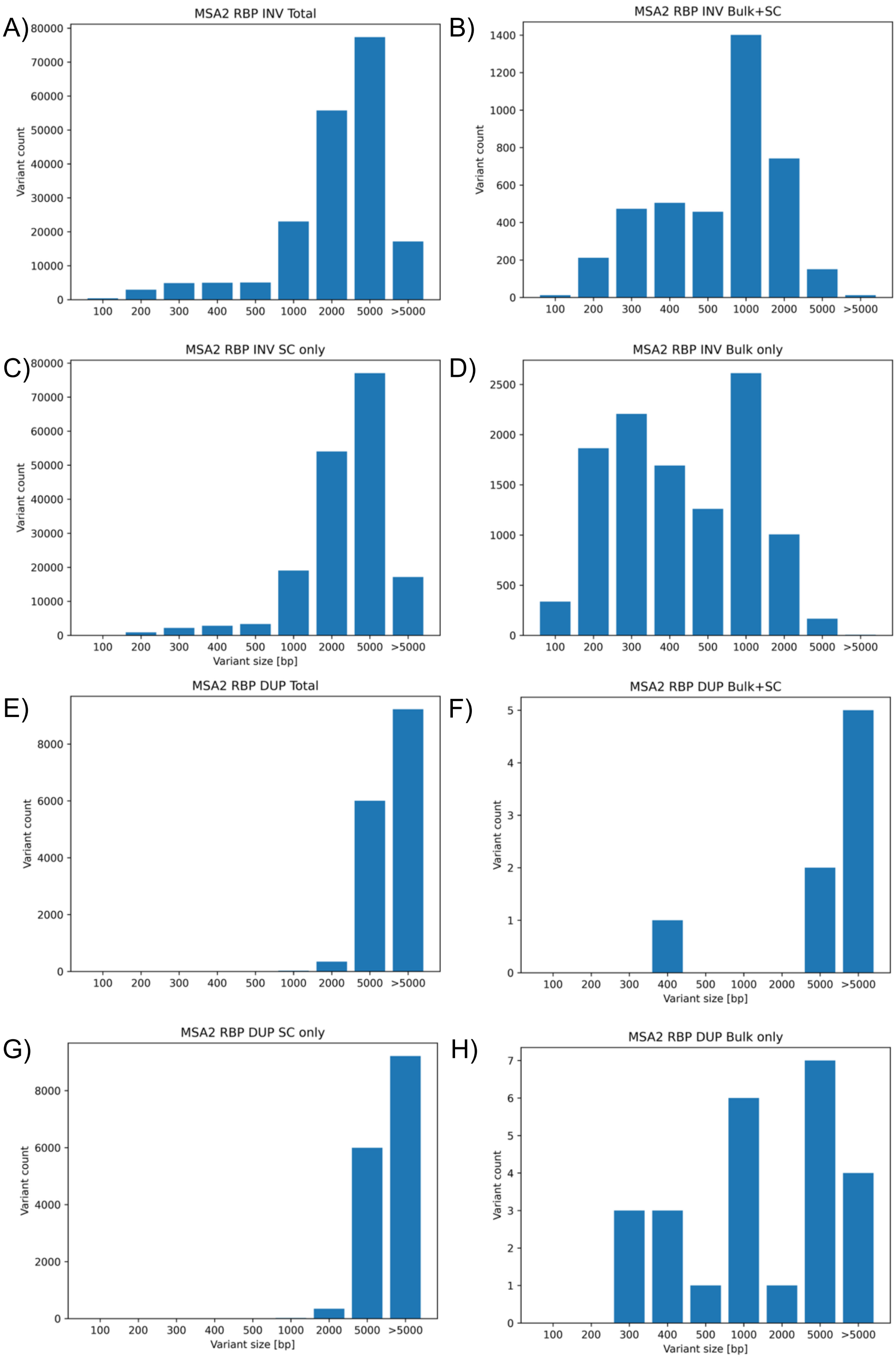

Supplementary Figure 52: Inversion and duplication sizes - unfiltered – MSA2 brain RBP library preparation A) Total number of reported inversions B) Inversions found in single cells and corresponding bulk C) Inversions found in single cells only D) Inversions found in bulk only E) Total number of reported duplications F) Duplications found in single cells and corresponding bulk G) Duplications found in single cells only H) Duplications found in bulk only.

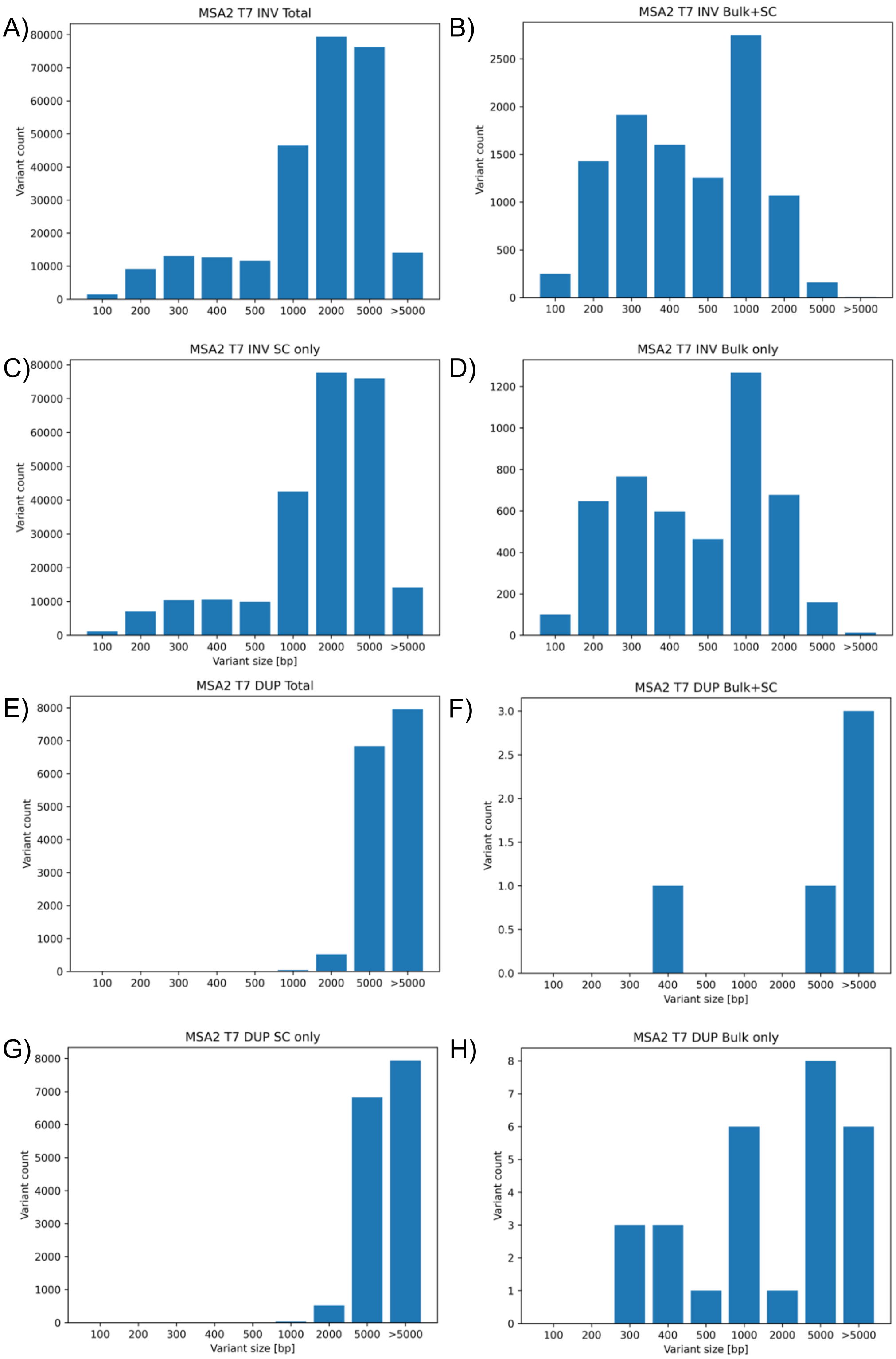

Supplementary Figure 53: Inversion and duplication sizes - unfiltered – MSA2 brain T7 library preparation A) Total number of reported inversions B) Inversions found in single cells and corresponding bulk C) Inversions found in single cells only D) Inversions found in bulk only E) Total number of reported duplications F) Duplications found in single cells and corresponding bulk G) Duplications found in single cells only H) Duplications found in bulk only.

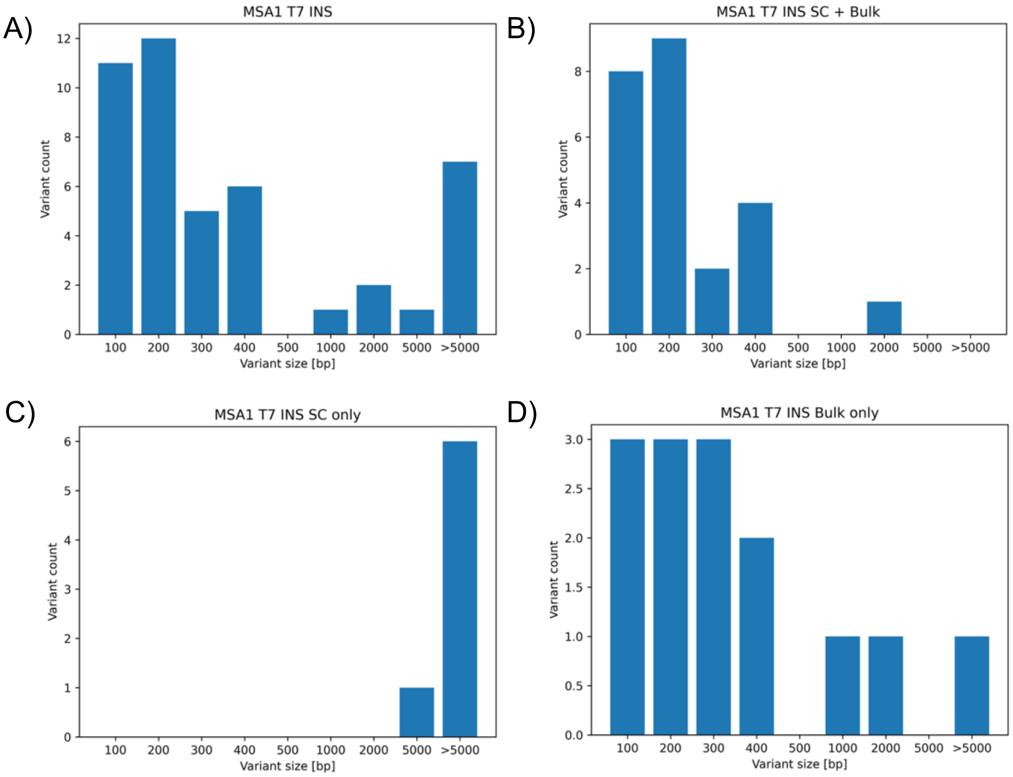

Supplementary figure 54: Insertion sizes in MSA1 brain T7 library preparation A) Total number of reported insertions B) Insertions found in single cells and corresponding bulk C) Insertions found in single cells only D) Insertions found in bulk only

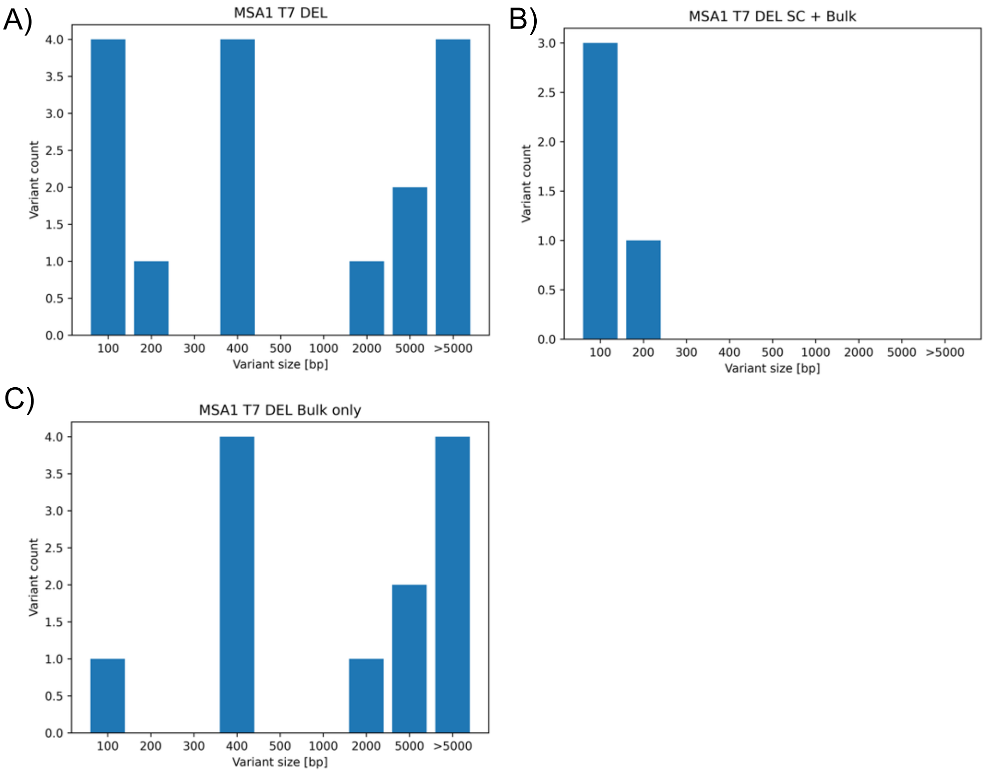

Supplementary figure 55: Deletion sizes in MSA1 brain T7 library preparation A) Total number of reported deletions B) Deletions found in single cells and corresponding bulk C) Deletions found in single cells only

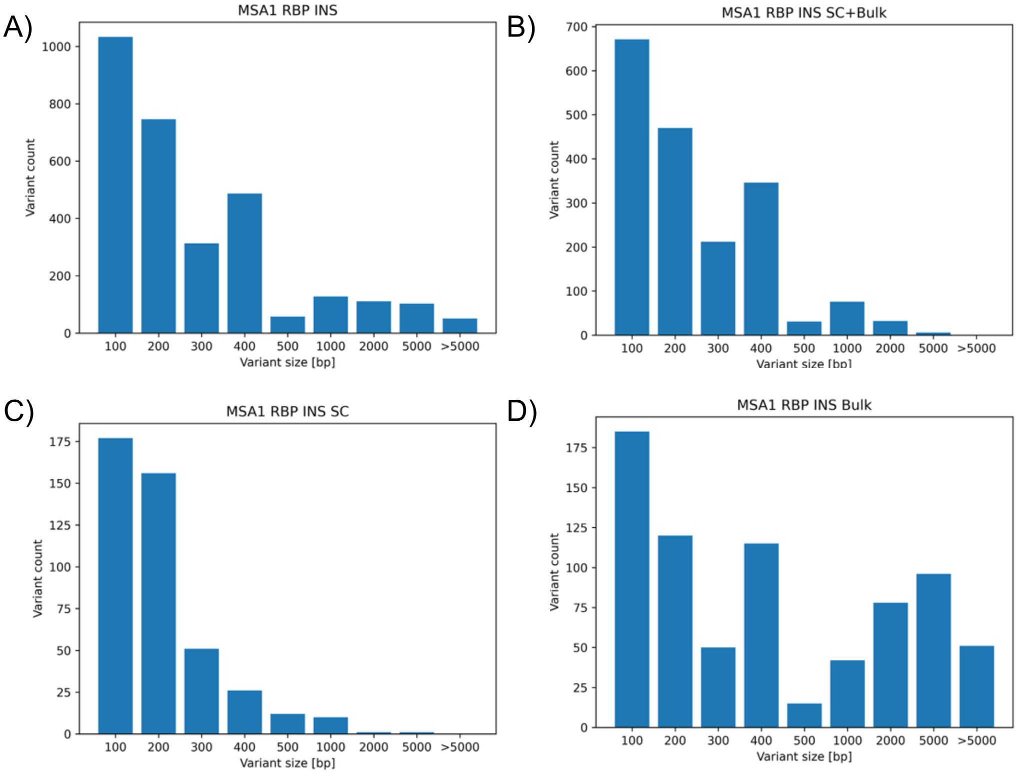

Supplementary figure 56: Insertion sizes in MSA1 brain RBP library preparation A) Total number of reported insertions B) Insertions found in single cells and corresponding bulk C) Insertions found in single cells only D) Insertions found in bulk only

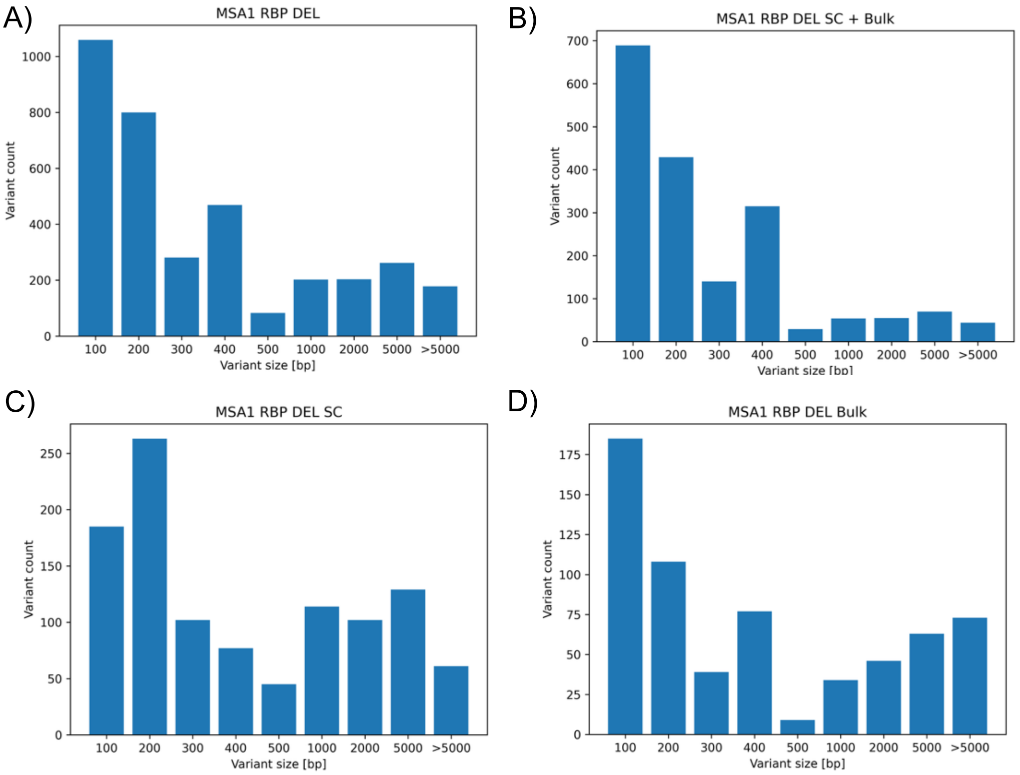

Supplementary figure 57: Deletion sizes in MSA1 brain RBP library preparation A) Total number of reported deletions B) Deletions found in single cells and corresponding bulk C) Deletions found in single cells only D) Deletions found in bulk only

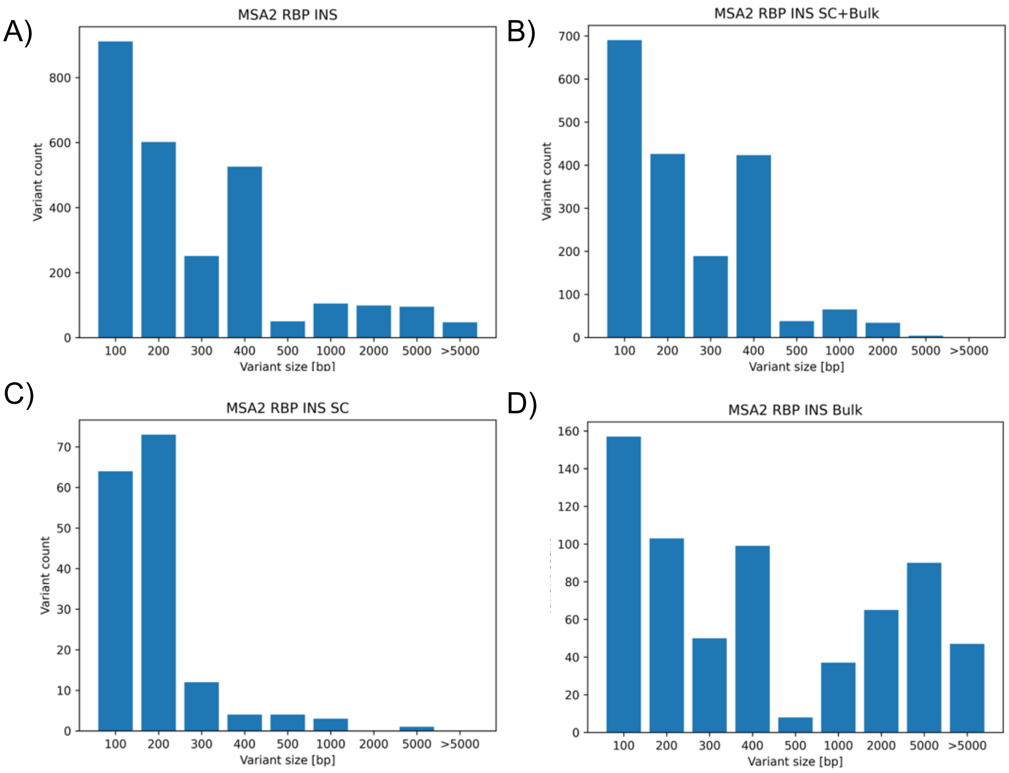

Supplementary figure 58: Insertion sizes in MSA2 brain RBP library preparation A) Total number of reported insertions B) Insertions found in single cells and corresponding bulk C) Insertions found in single cells only D) Insertions found in bulk only

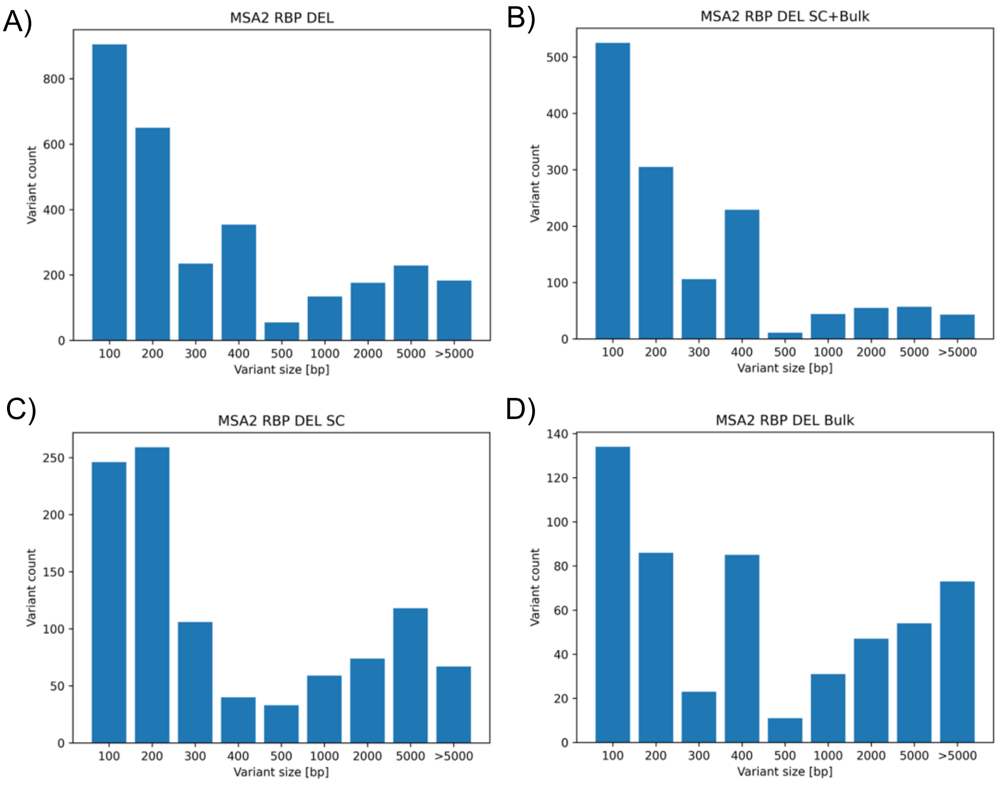

Supplementary figure 59: Deletion sizes in MSA2 brain RBP library preparation A) Total number of reported deletions B) Deletions found in single cells and corresponding bulk C) Deletions found in single cells only D) Deletions found in bulk only

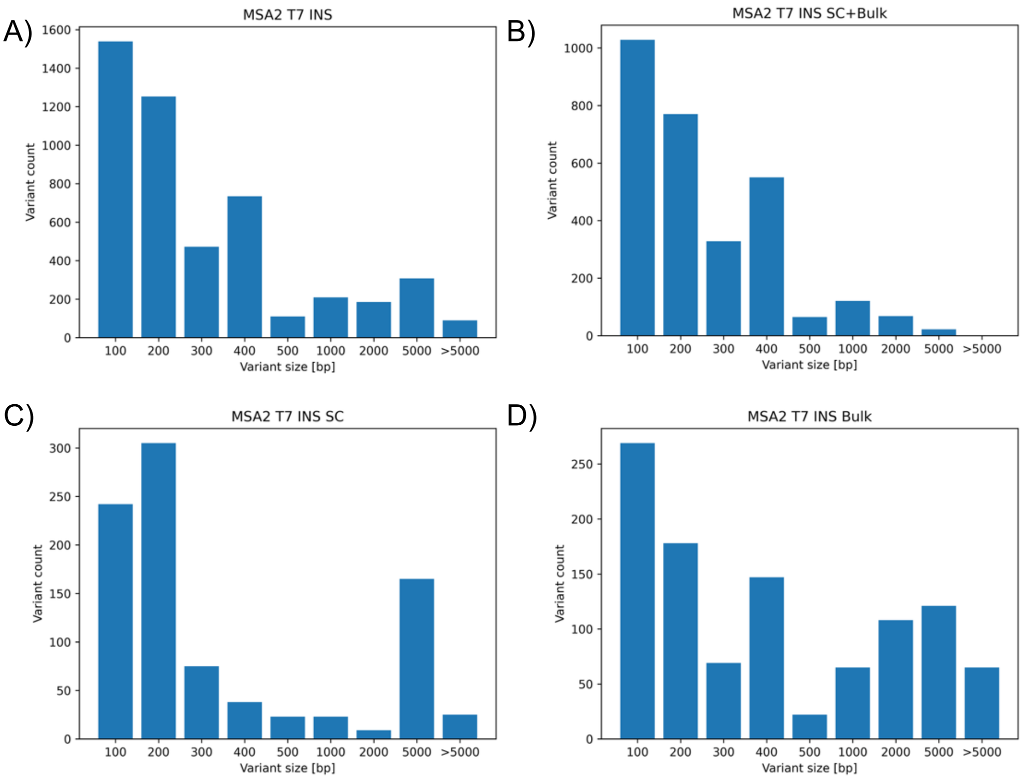

Supplementary figure 60: Insertion sizes in MSA2 brain T7 library preparation A) Total number of reported insertions B) Insertions found in single cells and corresponding bulk C) Insertions found in single cells only D) Insertions found in bulk only

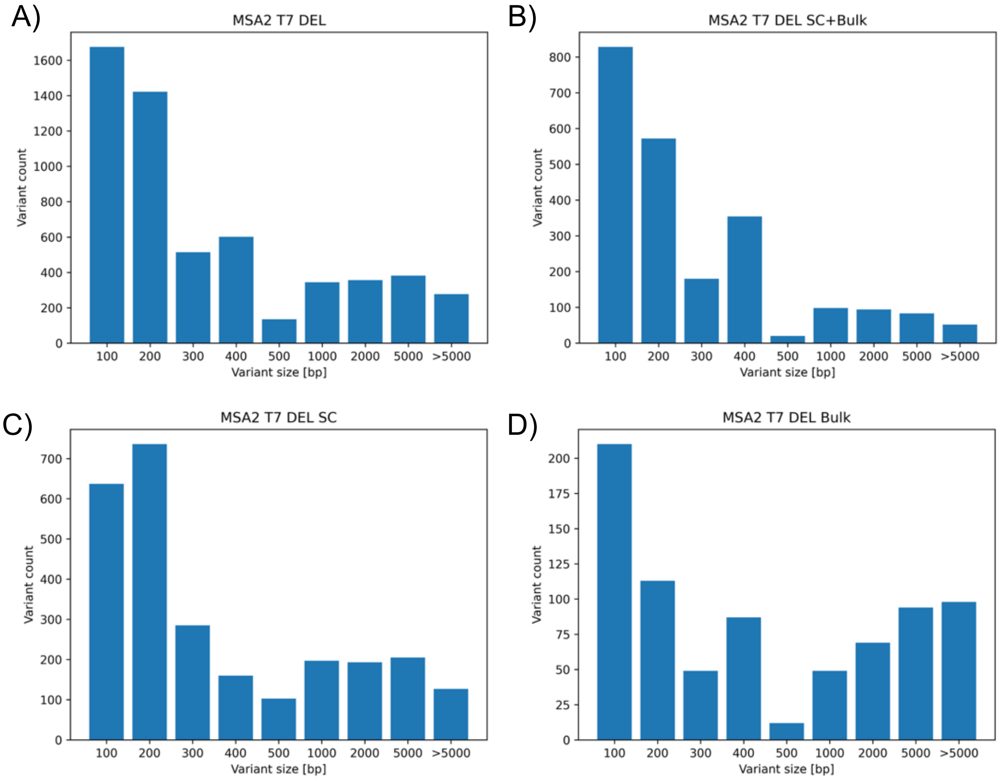

Supplementary figure 61: Deletion sizes in MSA2 brain T7 library preparation A) Total number of reported deletions B) Deletions found in single cells and corresponding bulk C) Deletions found in single cells only D) Deletions found in bulk only

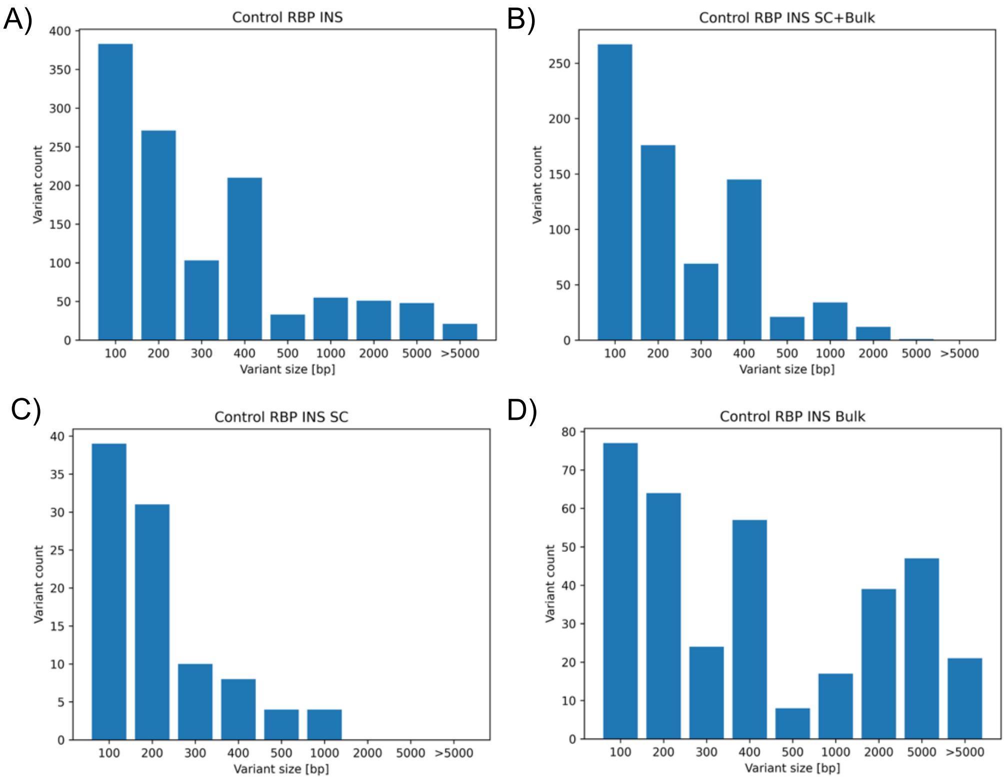

Supplementary figure 62: Insertion sizes in control brain RBP library preparation A) Total number of reported insertions B) Insertions found in single cells and corresponding bulk C) Insertions found in single cells only D) Insertions found in bulk only

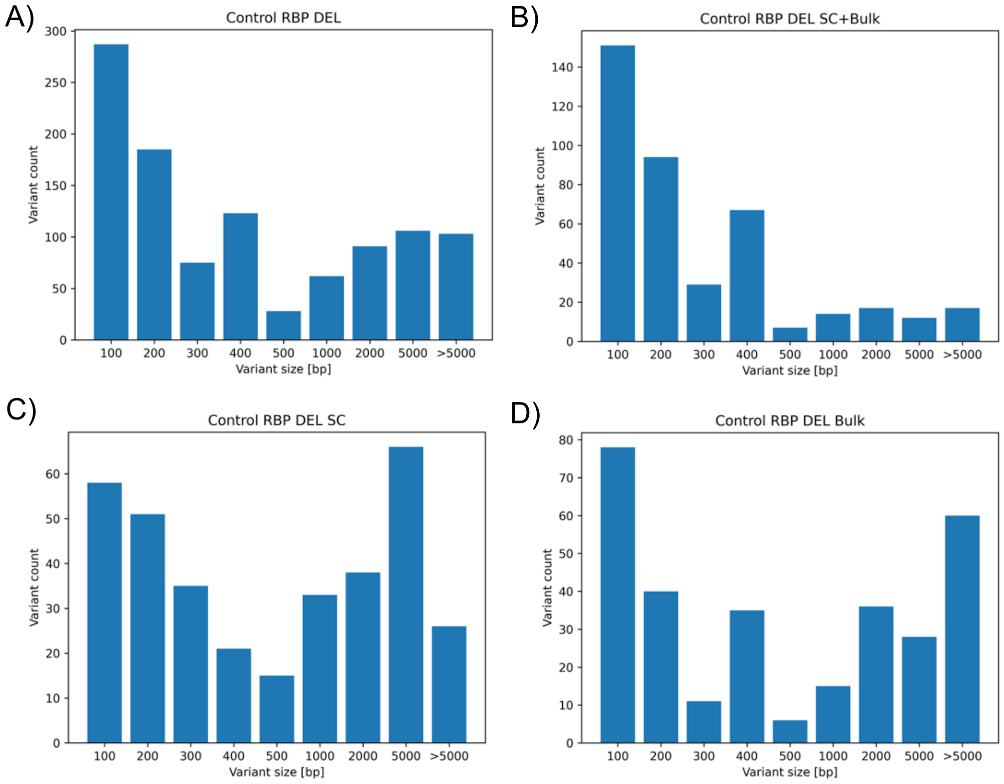

Supplementary figure 63: Deletion sizes in control brain RBP library preparation A) Total number of reported deletions B) Deletions found in single cells and corresponding bulk C) Deletions found in single cells only D) Deletions found in bulk only

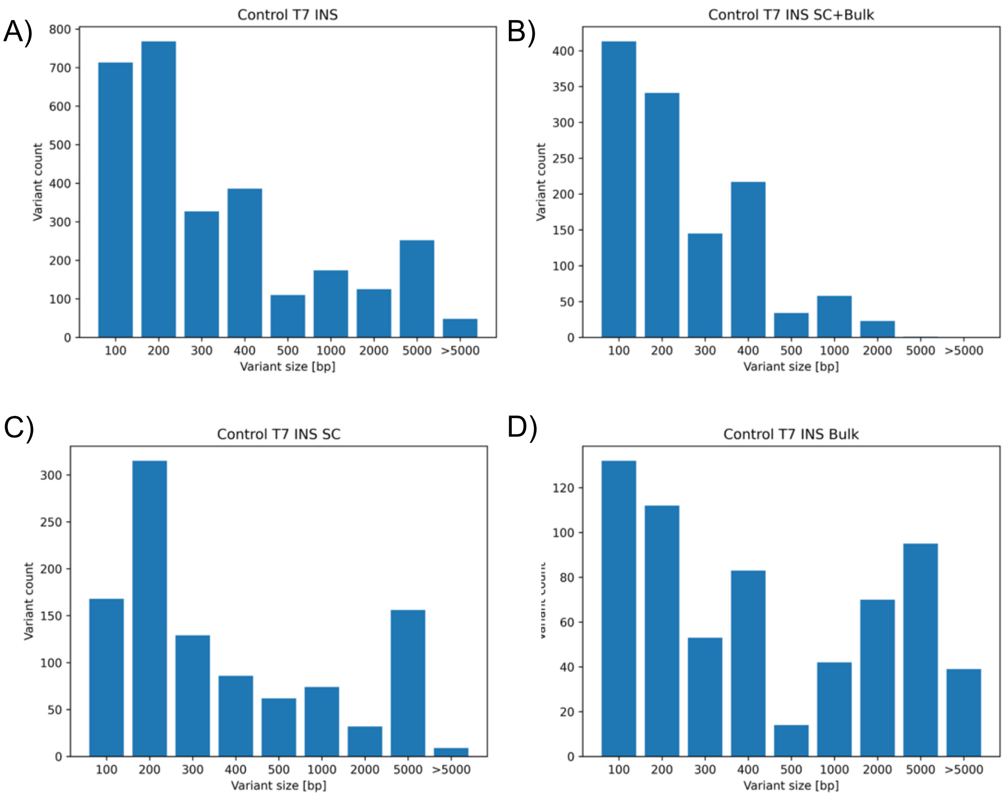

Supplementary figure 64: Insertion sizes in control brain T7 library preparation A) Total number of reported insertions B) Insertions found in single cells and corresponding bulk C) Insertions found in single cells only D) Insertions found in bulk only

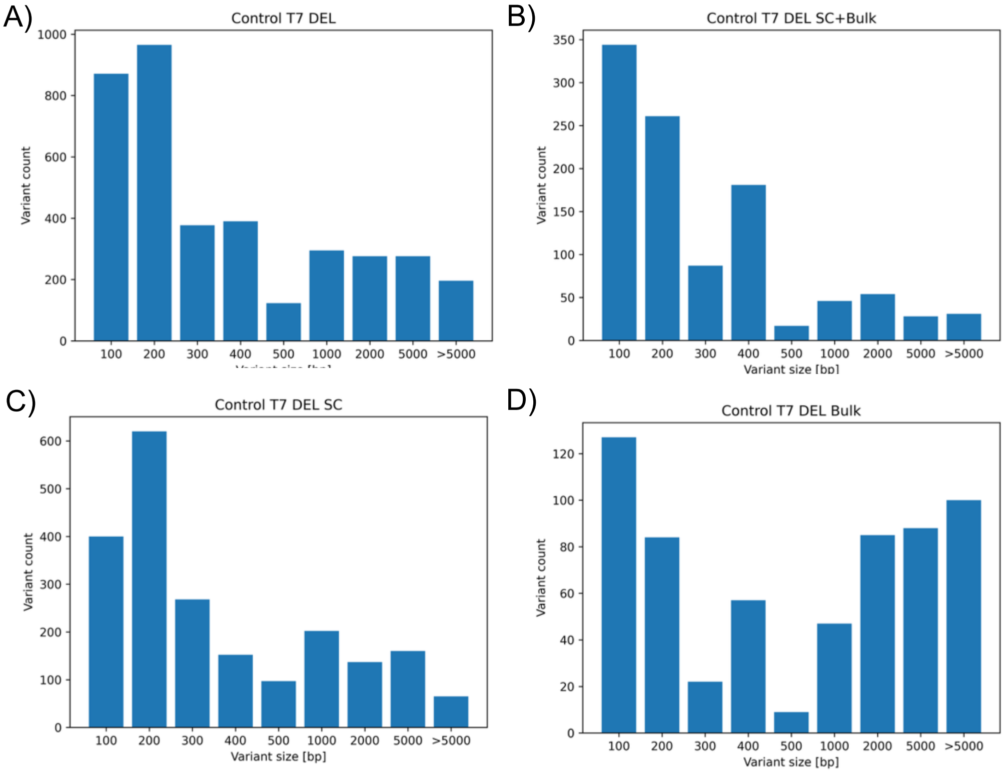

Supplementary figure 65: Deletion sizes in control brain T7 library preparation A) Total number of reported insertions B) Insertions found in single cells and corresponding bulk C) Insertions found in single cells only D) Insertions found in bulk only

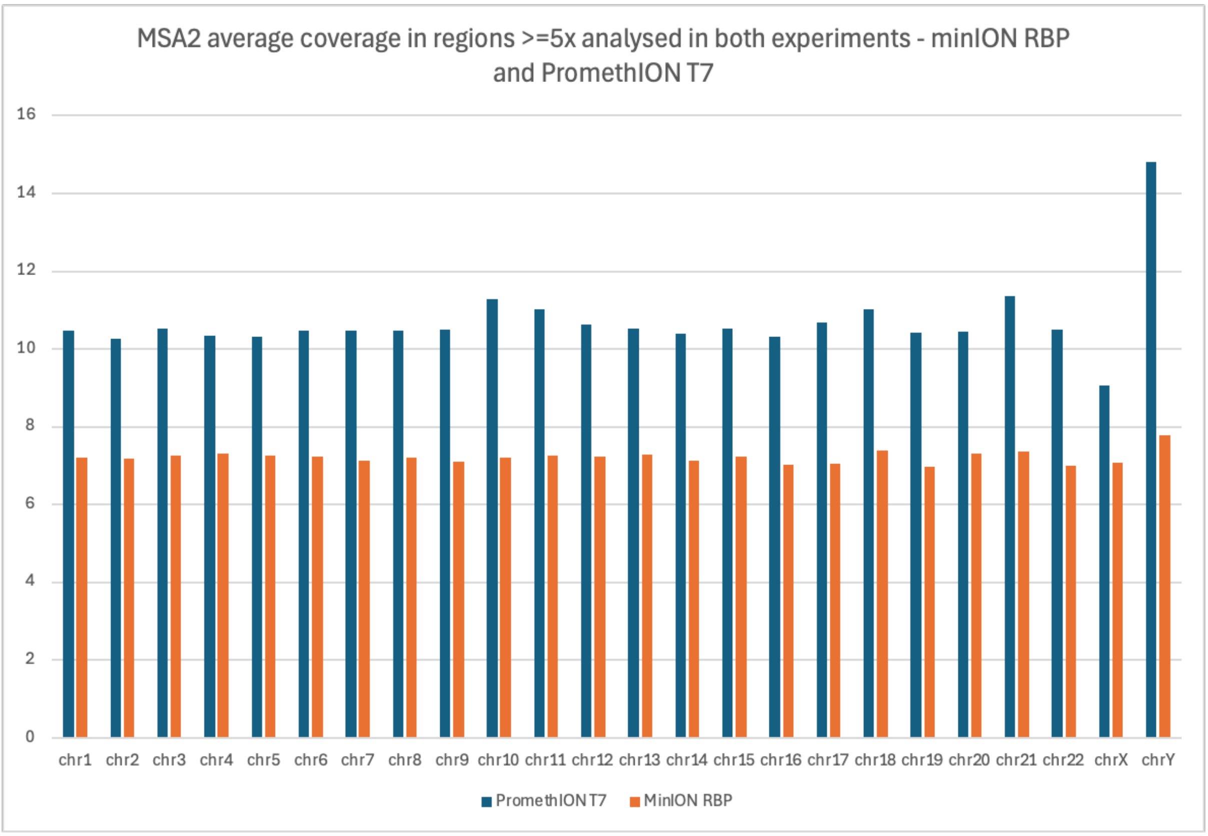

Supplementary Figure 66: Coverage comparison between MSA2 MinION RBP and PromethION T7 experiments.

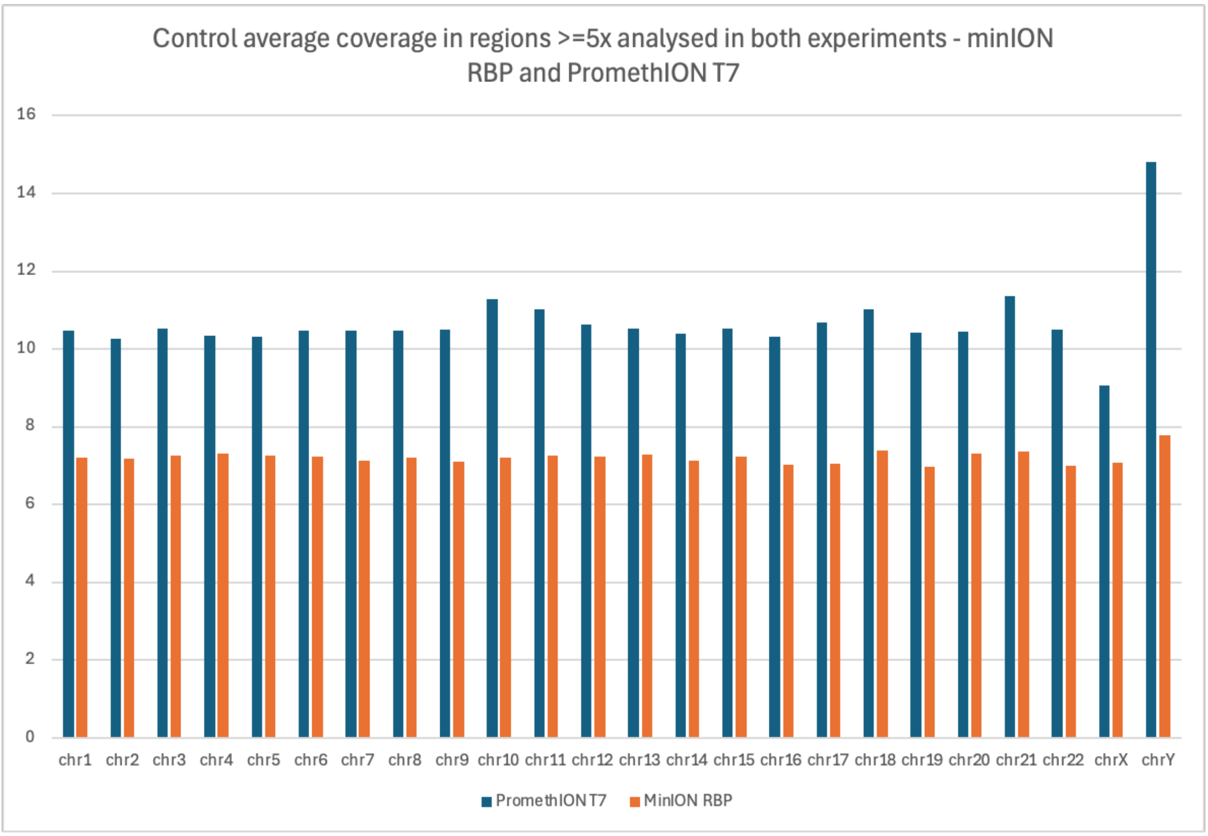

Supplementary Figure 67: Coverage comparison between control MinION RBP and PromethION T7 experiments.

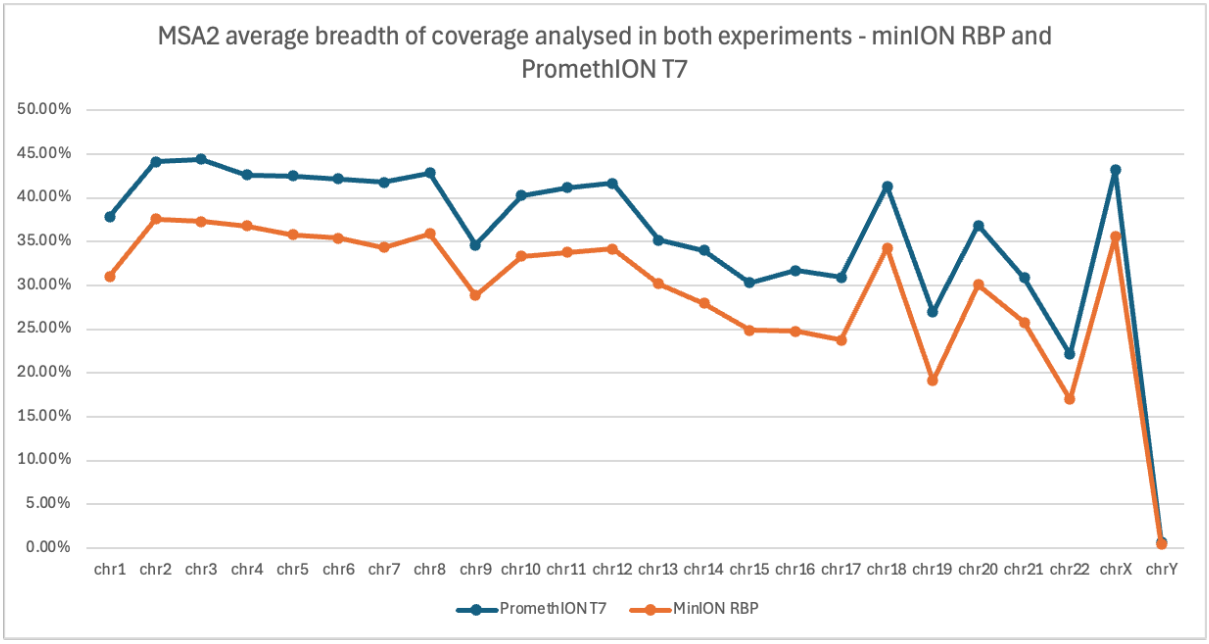

Supplementary Figure 68: Comparison of the breadth of coverage recovered in each experiment – MSA2.

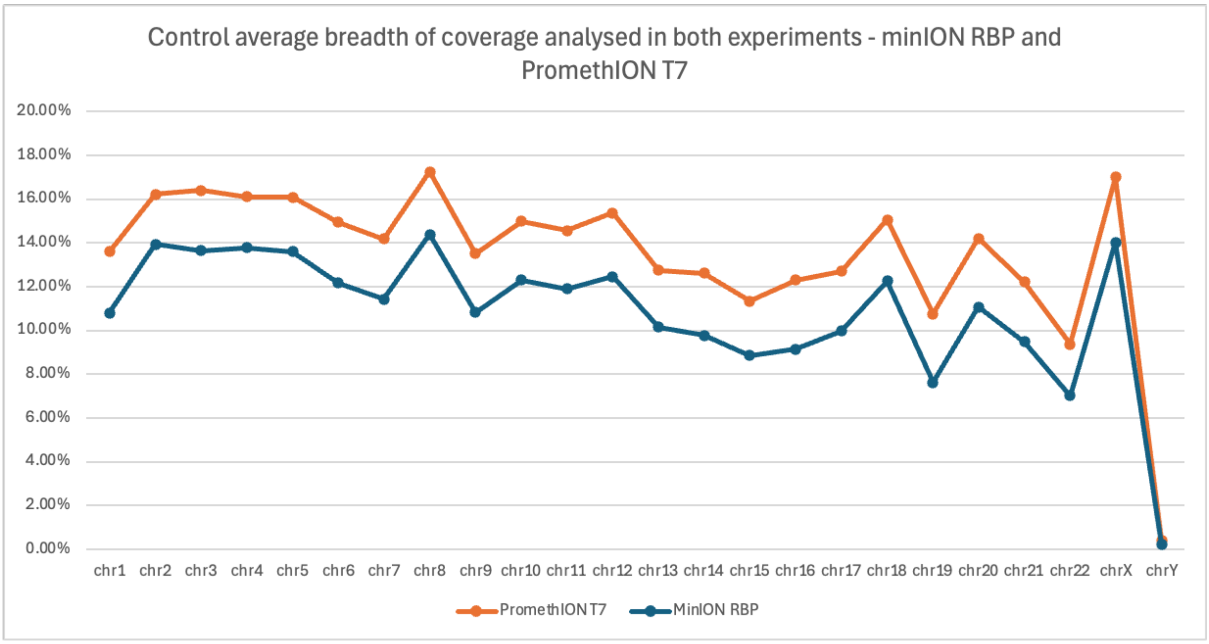

Supplementary Figure 69: Comparison of the breadth of coverage recovered in each experiment – Control.

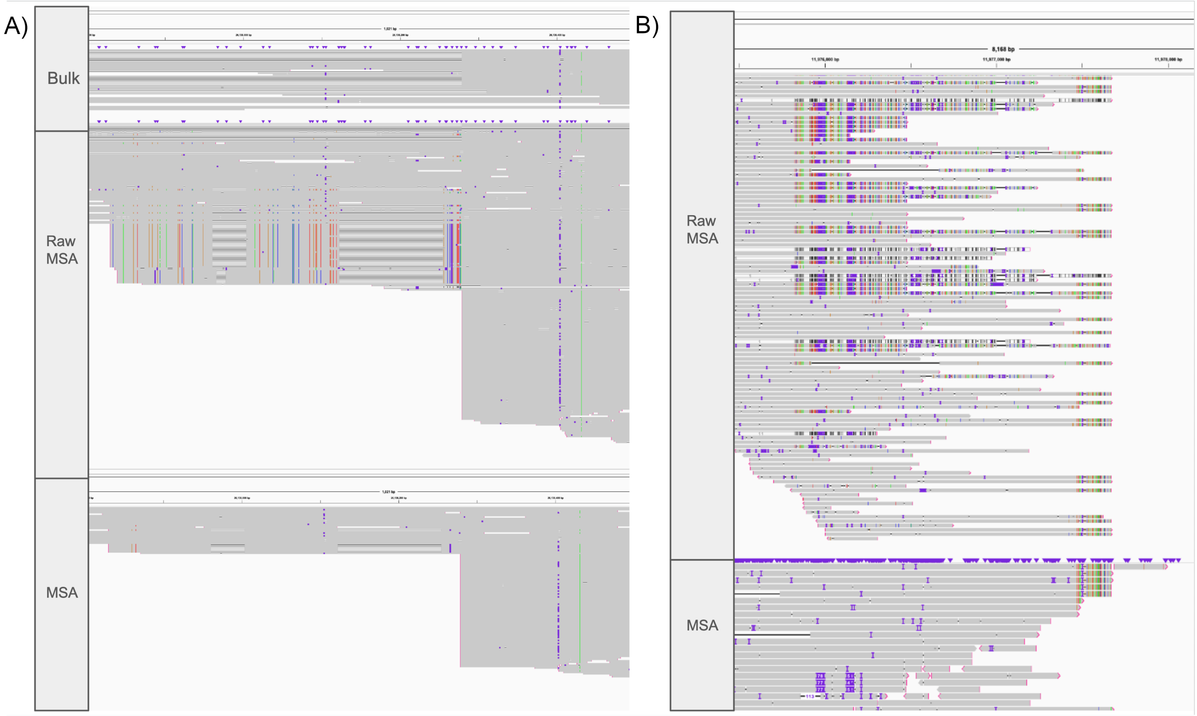

Supplementary Figure 70: Examples of chimeric reads observed in MSA brains. A) Chimeric reads, where fused part is misaligned to reference, creating apparent deletions and SNVs B) Multiple chimeric reads creating two major configurations of apparent SNVs and multiple small deletions. In both examples the “Raw MSA” sample is the original, unfiltered alignment and the “MSA” sample was filtered for chimeras; the observed variants are not reported after filtering.

Supplementary text

Supplementary text, Section 1: Detailed breakdown of transposable elements detected in brain samples.

On average, across the 2 MSA brains and 1 in control, we found 332 bulk insertions and 378 bulk deletions to contain LINE/L1 sequences (out of 2414 insertions and 1965 deletions, respectively). Collectively, in the corresponding single cell samples of these brains we captured 261 insertions and 686 deletions (out of 2249 and 2821, respectively) that similarly showed LINE/L1 fragments. Out of these variants, 94 insertions and 479 deletions were single-cell only, 168 insertions and 208 deletions were shared between bulk and single-cells and 165 insertions/170 deletions were bulk-only. Next, we performed a similar search for SINE/Alu elements. On average 689 insertions and 534 deletions detected in bulk contained SINE/Alu sequences, whereas for single-cell samples, 646 insertions and 775 deletions contained SINE/Alu. This breaks down into 164 insertions and 418 deletions containing SINE/Alu found only in single cells, with the remaining 482 insertions and 357 deletions shared between single cells and bulk and 207 insertions/177 deletions being bulk-specific.

Supplementary text, Section 2: Filter for chimeric SV caused by MDA amplification.

During our initial analysis of the Sniffles2 SV calls, we observed that some of them were overlapping regions of the reads that did not align to the reference genome correctly, showing a large proportion of misaligned bases and particularly, numerous deletions over a short span of the read, often close to its end (see **Supplementary figure 70**). Furthermore, some of the

reads had multiple splits, with the fragments clearly misaligned. Therefore, after testing multiple approaches, we determined to filter sequencing data, removing reads that had more than 1 split based on SAM “SA:” tag, as well as those, which more than 5% of their total bases differed from the reference genome, based on “MD:” tag. This filtering was applied to initial alignments of reads for each of the analyzed single-cell experiments, and the remaining reads were further re-evaluated for variants according to steps described in methods sections.
