# Supplementary material for: Single cell long read whole genome sequencing reveals somatic transposon activity in human brain
Source: Commun Biol. 2025 Nov 20;8:1627. doi: 10.1038/s42003-025-08805-2 (PMC12635067; doi:10.1038/s42003-025-08805-2)
Supplement: Supplementary file 2 — Description of additional supplementary file [file 42003_2025_8805_MOESM2_ESM.docx]

Description of additional supplementary file

File name: Supplementary Data 1

Description: This file contains numerical datasets organized into 32 tables. Each table includes the source information and all measured or calculated values generated during the analyses described in the manuscript. Data underlying all main and supplementary figures are provided. A key resources table is also included, detailing the assays, datasets, biological samples, software, and protocols used, with appropriate citations.
